# Supplementary material for: Pyruvate Dehydrogenase Kinase 1 inhibition mediated oxidative phosphorylation enhancement in cartilage promotes osteoarthritis progression
Source: BMC Musculoskelet Disord. 2023 Jul 20;24:597. doi: 10.1186/s12891-023-06585-6 (PMC10357736; doi:10.1186/s12891-023-06585-6)
Supplement: Supplementary file 1 — Additional file 1. [file 12891_2023_6585_MOESM1_ESM.zip › BP.Gsea.1653623667859/gsea_report_for_OA_1653623667859.html]

Report for OA 1653623667859 [GSEA]

| GS  follow link to MSigDB | GS DETAILS | SIZE | ES | NES | NOM p-val | FDR q-val | FWER p-val | RANK AT MAX | LEADING EDGE || 1 | GOBP\_ATP\_SYNTHESIS\_COUPLED\_ELECTRON\_TRANSPORT | Details ... | 67 | 0.67 | 2.95 | 0.000 | 0.000 | 0.000 | 2623 | tags=69%, list=20%, signal=86% |
| 2 | GOBP\_MITOCHONDRIAL\_ELECTRON\_TRANSPORT\_NADH\_TO\_UBIQUINONE | Details ... | 40 | 0.72 | 2.84 | 0.000 | 0.000 | 0.000 | 1800 | tags=75%, list=14%, signal=87% |
| 3 | GOBP\_OXIDATIVE\_PHOSPHORYLATION | Details ... | 102 | 0.59 | 2.75 | 0.000 | 0.000 | 0.000 | 2701 | tags=58%, list=21%, signal=73% |
| 4 | GOBP\_MITOCHONDRIAL\_RESPIRATORY\_CHAIN\_COMPLEX\_ASSEMBLY | Details ... | 58 | 0.65 | 2.75 | 0.000 | 0.000 | 0.000 | 2623 | tags=67%, list=20%, signal=84% |
| 5 | GOBP\_NADH\_DEHYDROGENASE\_COMPLEX\_ASSEMBLY | Details ... | 42 | 0.69 | 2.70 | 0.000 | 0.000 | 0.000 | 1229 | tags=57%, list=10%, signal=63% |
| 6 | GOBP\_AEROBIC\_RESPIRATION | Details ... | 146 | 0.54 | 2.68 | 0.000 | 0.000 | 0.000 | 2701 | tags=51%, list=21%, signal=64% |
| 7 | GOBP\_RESPIRATORY\_ELECTRON\_TRANSPORT\_CHAIN | Details ... | 83 | 0.58 | 2.65 | 0.000 | 0.000 | 0.000 | 2623 | tags=59%, list=20%, signal=74% |
| 8 | GOBP\_ELECTRON\_TRANSPORT\_CHAIN | Details ... | 133 | 0.52 | 2.53 | 0.000 | 0.000 | 0.000 | 2517 | tags=49%, list=20%, signal=60% |
| 9 | GOBP\_CELLULAR\_RESPIRATION | Details ... | 177 | 0.45 | 2.32 | 0.000 | 0.000 | 0.001 | 2517 | tags=43%, list=20%, signal=53% |
| 10 | GOBP\_INTRA\_GOLGI\_VESICLE\_MEDIATED\_TRANSPORT | Details ... | 24 | 0.62 | 2.16 | 0.000 | 0.004 | 0.059 | 1959 | tags=54%, list=15%, signal=64% |
| 11 | GOBP\_PROTON\_TRANSMEMBRANE\_TRANSPORT | Details ... | 109 | 0.45 | 2.14 | 0.000 | 0.005 | 0.077 | 2941 | tags=49%, list=23%, signal=62% |
| 12 | GOBP\_MITOCHONDRIAL\_TRANSLATION | Details ... | 50 | 0.53 | 2.12 | 0.000 | 0.006 | 0.098 | 2515 | tags=46%, list=19%, signal=57% |
| 13 | GOBP\_RETROGRADE\_VESICLE\_MEDIATED\_TRANSPORT\_GOLGI\_TO\_ENDOPLASMIC\_RETICULUM | Details ... | 45 | 0.53 | 2.10 | 0.000 | 0.006 | 0.118 | 2227 | tags=47%, list=17%, signal=56% |
| 14 | GOBP\_ATP\_METABOLIC\_PROCESS | Details ... | 220 | 0.40 | 2.08 | 0.000 | 0.008 | 0.159 | 2643 | tags=40%, list=20%, signal=49% |
| 15 | GOBP\_B\_CELL\_RECEPTOR\_SIGNALING\_PATHWAY | Details ... | 66 | 0.46 | 2.00 | 0.000 | 0.021 | 0.378 | 3449 | tags=56%, list=27%, signal=76% |
| 16 | GOBP\_ATP\_SYNTHESIS\_COUPLED\_PROTON\_TRANSPORT | Details ... | 20 | 0.63 | 1.98 | 0.000 | 0.024 | 0.442 | 3024 | tags=70%, list=23%, signal=91% |
| 17 | GOBP\_CYTOCHROME\_COMPLEX\_ASSEMBLY | Details ... | 18 | 0.63 | 1.96 | 0.002 | 0.029 | 0.526 | 2473 | tags=50%, list=19%, signal=62% |
| 18 | GOBP\_ENDOPLASMIC\_RETICULUM\_TO\_GOLGI\_VESICLE\_MEDIATED\_TRANSPORT | Details ... | 107 | 0.42 | 1.96 | 0.000 | 0.029 | 0.540 | 2600 | tags=35%, list=20%, signal=43% |
| 19 | GOBP\_GPI\_ANCHOR\_METABOLIC\_PROCESS | Details ... | 26 | 0.57 | 1.95 | 0.002 | 0.029 | 0.565 | 2801 | tags=58%, list=22%, signal=74% |
| 20 | GOBP\_ENERGY\_DERIVATION\_BY\_OXIDATION\_OF\_ORGANIC\_COMPOUNDS | Details ... | 252 | 0.36 | 1.93 | 0.000 | 0.036 | 0.660 | 2460 | tags=35%, list=19%, signal=43% |
| 21 | GOBP\_LATE\_ENDOSOME\_TO\_VACUOLE\_TRANSPORT | Details ... | 18 | 0.61 | 1.91 | 0.002 | 0.042 | 0.721 | 3037 | tags=67%, list=24%, signal=87% |
| 22 | GOBP\_GENERATION\_OF\_PRECURSOR\_METABOLITES\_AND\_ENERGY | Details ... | 406 | 0.34 | 1.90 | 0.000 | 0.044 | 0.752 | 2701 | tags=35%, list=21%, signal=43% |
| 23 | GOBP\_PROTEIN\_INSERTION\_INTO\_ER\_MEMBRANE | Details ... | 19 | 0.60 | 1.89 | 0.000 | 0.048 | 0.796 | 1624 | tags=53%, list=13%, signal=60% |
| 24 | GOBP\_GLYCOSYL\_COMPOUND\_METABOLIC\_PROCESS | Details ... | 69 | 0.44 | 1.89 | 0.000 | 0.046 | 0.796 | 3603 | tags=57%, list=28%, signal=78% |
| 25 | GOBP\_I\_KAPPAB\_PHOSPHORYLATION | Details ... | 18 | 0.59 | 1.88 | 0.002 | 0.048 | 0.819 | 2098 | tags=44%, list=16%, signal=53% |
| 26 | GOBP\_NEGATIVE\_REGULATION\_OF\_MITOCHONDRION\_ORGANIZATION | Details ... | 40 | 0.48 | 1.88 | 0.000 | 0.047 | 0.820 | 3005 | tags=40%, list=23%, signal=52% |
| 27 | GOBP\_PEPTIDYL\_ASPARAGINE\_MODIFICATION | Details ... | 18 | 0.60 | 1.88 | 0.005 | 0.046 | 0.825 | 1565 | tags=39%, list=12%, signal=44% |
| 28 | GOBP\_MISMATCH\_REPAIR | Details ... | 29 | 0.51 | 1.84 | 0.000 | 0.066 | 0.935 | 1557 | tags=34%, list=12%, signal=39% |
| 29 | GOBP\_PEROXISOME\_ORGANIZATION | Details ... | 31 | 0.50 | 1.83 | 0.000 | 0.071 | 0.949 | 1253 | tags=32%, list=10%, signal=36% |
| 30 | GOBP\_PROTEIN\_N\_LINKED\_GLYCOSYLATION | Details ... | 54 | 0.44 | 1.82 | 0.000 | 0.069 | 0.950 | 1565 | tags=30%, list=12%, signal=34% |
| 31 | GOBP\_GLYCOSIDE\_METABOLIC\_PROCESS | Details ... | 17 | 0.59 | 1.81 | 0.004 | 0.074 | 0.973 | 3327 | tags=65%, list=26%, signal=87% |
| 32 | GOBP\_NEGATIVE\_REGULATION\_OF\_RELEASE\_OF\_CYTOCHROME\_C\_FROM\_MITOCHONDRIA | Details ... | 18 | 0.58 | 1.79 | 0.002 | 0.085 | 0.986 | 2547 | tags=50%, list=20%, signal=62% |
| 33 | GOBP\_PROTEIN\_LOCALIZATION\_TO\_ENDOPLASMIC\_RETICULUM | Details ... | 54 | 0.44 | 1.79 | 0.000 | 0.089 | 0.989 | 1766 | tags=31%, list=14%, signal=36% |
| 34 | GOBP\_ENDOSOME\_TRANSPORT\_VIA\_MULTIVESICULAR\_BODY\_SORTING\_PATHWAY | Details ... | 22 | 0.53 | 1.79 | 0.009 | 0.087 | 0.990 | 3518 | tags=64%, list=27%, signal=87% |
| 35 | GOBP\_MEMBRANE\_INVAGINATION | Details ... | 79 | 0.40 | 1.77 | 0.002 | 0.094 | 0.993 | 3562 | tags=53%, list=28%, signal=73% |
| 36 | GOBP\_GOLGI\_VESICLE\_TRANSPORT | Details ... | 237 | 0.33 | 1.76 | 0.000 | 0.103 | 0.998 | 2780 | tags=32%, list=22%, signal=40% |
| 37 | GOBP\_VACUOLAR\_ACIDIFICATION | Details ... | 19 | 0.55 | 1.74 | 0.007 | 0.122 | 0.998 | 1304 | tags=32%, list=10%, signal=35% |
| 38 | GOBP\_VIRION\_ASSEMBLY | Details ... | 26 | 0.50 | 1.73 | 0.000 | 0.131 | 0.999 | 3037 | tags=62%, list=24%, signal=80% |
| 39 | GOBP\_HUMORAL\_IMMUNE\_RESPONSE\_MEDIATED\_BY\_CIRCULATING\_IMMUNOGLOBULIN | Details ... | 65 | 0.39 | 1.69 | 0.003 | 0.171 | 1.000 | 3449 | tags=51%, list=27%, signal=69% |
| 40 | GOBP\_SOMATIC\_DIVERSIFICATION\_OF\_IMMUNE\_RECEPTORS\_VIA\_SOMATIC\_MUTATION | Details ... | 15 | 0.57 | 1.69 | 0.014 | 0.168 | 1.000 | 1825 | tags=33%, list=14%, signal=39% |
| 41 | GOBP\_MITOCHONDRIAL\_FUSION | Details ... | 23 | 0.51 | 1.69 | 0.005 | 0.167 | 1.000 | 2279 | tags=39%, list=18%, signal=47% |
| 42 | GOBP\_LIPOSACCHARIDE\_METABOLIC\_PROCESS | Details ... | 85 | 0.37 | 1.65 | 0.000 | 0.218 | 1.000 | 3407 | tags=48%, list=26%, signal=65% |
| 43 | GOBP\_PROTEIN\_INSERTION\_INTO\_MEMBRANE | Details ... | 47 | 0.41 | 1.65 | 0.008 | 0.214 | 1.000 | 1273 | tags=28%, list=10%, signal=31% |
| 44 | GOBP\_TETRAPYRROLE\_BIOSYNTHETIC\_PROCESS | Details ... | 28 | 0.47 | 1.65 | 0.005 | 0.220 | 1.000 | 905 | tags=25%, list=7%, signal=27% |
| 45 | GOBP\_REGULATION\_OF\_RELEASE\_OF\_CYTOCHROME\_C\_FROM\_MITOCHONDRIA | Details ... | 43 | 0.42 | 1.65 | 0.007 | 0.216 | 1.000 | 2286 | tags=35%, list=18%, signal=42% |
| 46 | GOBP\_NUCLEOSIDE\_TRIPHOSPHATE\_BIOSYNTHETIC\_PROCESS | Details ... | 66 | 0.38 | 1.64 | 0.005 | 0.226 | 1.000 | 3024 | tags=42%, list=23%, signal=55% |
| 47 | GOBP\_GLUTATHIONE\_METABOLIC\_PROCESS | Details ... | 52 | 0.39 | 1.63 | 0.010 | 0.227 | 1.000 | 2583 | tags=37%, list=20%, signal=46% |
| 48 | GOBP\_N\_GLYCAN\_PROCESSING | Details ... | 17 | 0.53 | 1.63 | 0.021 | 0.222 | 1.000 | 1850 | tags=47%, list=14%, signal=55% |
| 49 | GOBP\_MITOCHONDRIAL\_GENE\_EXPRESSION | Details ... | 75 | 0.37 | 1.63 | 0.006 | 0.220 | 1.000 | 2515 | tags=36%, list=19%, signal=44% |
| 50 | GOBP\_MEMBRANE\_LIPID\_CATABOLIC\_PROCESS | Details ... | 28 | 0.46 | 1.62 | 0.005 | 0.230 | 1.000 | 3462 | tags=61%, list=27%, signal=83% |
| 51 | GOBP\_IMMUNOGLOBULIN\_PRODUCTION | Details ... | 120 | 0.34 | 1.61 | 0.000 | 0.247 | 1.000 | 2540 | tags=34%, list=20%, signal=42% |
| 52 | GOBP\_DEOXYRIBOSE\_PHOSPHATE\_METABOLIC\_PROCESS | Details ... | 35 | 0.42 | 1.60 | 0.017 | 0.274 | 1.000 | 1852 | tags=31%, list=14%, signal=37% |
| 53 | GOBP\_PURINE\_NUCLEOSIDE\_METABOLIC\_PROCESS | Details ... | 20 | 0.50 | 1.59 | 0.019 | 0.271 | 1.000 | 1955 | tags=40%, list=15%, signal=47% |
| 54 | GOBP\_MITOCHONDRION\_ORGANIZATION | Details ... | 394 | 0.28 | 1.59 | 0.000 | 0.272 | 1.000 | 2580 | tags=29%, list=20%, signal=35% |
| 55 | GOBP\_POSITIVE\_REGULATION\_OF\_MITOCHONDRIAL\_FISSION | Details ... | 16 | 0.52 | 1.59 | 0.025 | 0.270 | 1.000 | 2150 | tags=56%, list=17%, signal=67% |
| 56 | GOBP\_PURINE\_RIBONUCLEOSIDE\_METABOLIC\_PROCESS | Details ... | 17 | 0.51 | 1.58 | 0.019 | 0.282 | 1.000 | 1955 | tags=41%, list=15%, signal=48% |
| 57 | GOBP\_DICARBOXYLIC\_ACID\_METABOLIC\_PROCESS | Details ... | 74 | 0.36 | 1.58 | 0.010 | 0.289 | 1.000 | 2534 | tags=42%, list=20%, signal=52% |
| 58 | GOBP\_ACETYL\_COA\_BIOSYNTHETIC\_PROCESS | Details ... | 16 | 0.51 | 1.57 | 0.018 | 0.294 | 1.000 | 2433 | tags=50%, list=19%, signal=62% |
| 59 | GOBP\_GLYCOSYLATION | Details ... | 168 | 0.31 | 1.57 | 0.000 | 0.294 | 1.000 | 1691 | tags=23%, list=13%, signal=26% |
| 60 | GOBP\_ENDOPLASMIC\_RETICULUM\_ORGANIZATION | Details ... | 65 | 0.36 | 1.57 | 0.008 | 0.289 | 1.000 | 1161 | tags=25%, list=9%, signal=27% |
| 61 | GOBP\_NUCLEOSIDE\_BISPHOSPHATE\_BIOSYNTHETIC\_PROCESS | Details ... | 51 | 0.38 | 1.57 | 0.015 | 0.290 | 1.000 | 2520 | tags=37%, list=20%, signal=46% |
| 62 | GOBP\_MAINTENANCE\_OF\_PROTEIN\_LOCALIZATION\_IN\_ORGANELLE | Details ... | 37 | 0.42 | 1.56 | 0.015 | 0.288 | 1.000 | 1486 | tags=30%, list=12%, signal=34% |
| 63 | GOBP\_RIBONUCLEOSIDE\_TRIPHOSPHATE\_BIOSYNTHETIC\_PROCESS | Details ... | 59 | 0.37 | 1.56 | 0.005 | 0.287 | 1.000 | 3024 | tags=44%, list=23%, signal=57% |
| 64 | GOBP\_NUCLEOSIDE\_METABOLIC\_PROCESS | Details ... | 50 | 0.38 | 1.56 | 0.008 | 0.283 | 1.000 | 3830 | tags=56%, list=30%, signal=79% |
| 65 | GOBP\_REGULATION\_OF\_ANTIGEN\_PROCESSING\_AND\_PRESENTATION | Details ... | 19 | 0.50 | 1.56 | 0.037 | 0.282 | 1.000 | 2147 | tags=37%, list=17%, signal=44% |
| 66 | GOBP\_AROMATIC\_AMINO\_ACID\_FAMILY\_METABOLIC\_PROCESS | Details ... | 21 | 0.47 | 1.54 | 0.033 | 0.316 | 1.000 | 2502 | tags=48%, list=19%, signal=59% |
| 67 | GOBP\_TRICARBOXYLIC\_ACID\_CYCLE | Details ... | 28 | 0.43 | 1.54 | 0.015 | 0.313 | 1.000 | 3228 | tags=54%, list=25%, signal=71% |
| 68 | GOBP\_ESTABLISHMENT\_OF\_PROTEIN\_LOCALIZATION\_TO\_ENDOPLASMIC\_RETICULUM | Details ... | 31 | 0.42 | 1.54 | 0.025 | 0.311 | 1.000 | 1766 | tags=29%, list=14%, signal=34% |
| 69 | GOBP\_CELLULAR\_RESPONSE\_TO\_TOXIC\_SUBSTANCE | Details ... | 93 | 0.34 | 1.54 | 0.008 | 0.316 | 1.000 | 2161 | tags=32%, list=17%, signal=38% |
| 70 | GOBP\_PROTEIN\_TARGETING\_TO\_PEROXISOME | Details ... | 17 | 0.48 | 1.52 | 0.052 | 0.355 | 1.000 | 1253 | tags=29%, list=10%, signal=33% |
| 71 | GOBP\_ACTIN\_MYOSIN\_FILAMENT\_SLIDING | Details ... | 15 | 0.51 | 1.52 | 0.050 | 0.350 | 1.000 | 3161 | tags=60%, list=25%, signal=79% |
| 72 | GOBP\_REGULATION\_OF\_SUPEROXIDE\_METABOLIC\_PROCESS | Details ... | 28 | 0.42 | 1.51 | 0.036 | 0.360 | 1.000 | 2490 | tags=36%, list=19%, signal=44% |
| 73 | GOBP\_CARBOHYDRATE\_DERIVATIVE\_BIOSYNTHETIC\_PROCESS | Details ... | 464 | 0.27 | 1.51 | 0.000 | 0.360 | 1.000 | 2663 | tags=28%, list=21%, signal=35% |
| 74 | GOBP\_POST\_TRANSLATIONAL\_PROTEIN\_MODIFICATION | Details ... | 27 | 0.43 | 1.51 | 0.029 | 0.359 | 1.000 | 3372 | tags=48%, list=26%, signal=65% |
| 75 | GOBP\_BILE\_ACID\_BIOSYNTHETIC\_PROCESS | Details ... | 29 | 0.41 | 1.50 | 0.040 | 0.377 | 1.000 | 1564 | tags=21%, list=12%, signal=23% |
| 76 | GOBP\_TRNA\_PROCESSING | Details ... | 85 | 0.33 | 1.49 | 0.013 | 0.391 | 1.000 | 1686 | tags=25%, list=13%, signal=28% |
| 77 | GOBP\_DEOXYRIBONUCLEOSIDE\_MONOPHOSPHATE\_METABOLIC\_PROCESS | Details ... | 20 | 0.47 | 1.49 | 0.060 | 0.387 | 1.000 | 1107 | tags=30%, list=9%, signal=33% |
| 78 | GOBP\_NEGATIVE\_REGULATION\_OF\_B\_CELL\_PROLIFERATION | Details ... | 16 | 0.50 | 1.49 | 0.069 | 0.392 | 1.000 | 3202 | tags=56%, list=25%, signal=75% |
| 79 | GOBP\_NUCLEOTIDE\_EXCISION\_REPAIR | Details ... | 53 | 0.36 | 1.49 | 0.034 | 0.390 | 1.000 | 1696 | tags=28%, list=13%, signal=32% |
| 80 | GOBP\_ATP\_BIOSYNTHETIC\_PROCESS | Details ... | 45 | 0.37 | 1.48 | 0.034 | 0.391 | 1.000 | 3518 | tags=51%, list=27%, signal=70% |
| 81 | GOBP\_RIBOSE\_PHOSPHATE\_BIOSYNTHETIC\_PROCESS | Details ... | 161 | 0.30 | 1.48 | 0.000 | 0.390 | 1.000 | 3024 | tags=35%, list=23%, signal=46% |
| 82 | GOBP\_CELLULAR\_METABOLIC\_COMPOUND\_SALVAGE | Details ... | 23 | 0.44 | 1.48 | 0.039 | 0.387 | 1.000 | 2004 | tags=35%, list=16%, signal=41% |
| 83 | GOBP\_SULFUR\_COMPOUND\_METABOLIC\_PROCESS | Details ... | 267 | 0.28 | 1.48 | 0.000 | 0.383 | 1.000 | 3407 | tags=39%, list=26%, signal=51% |
| 84 | GOBP\_GLYCOSYL\_COMPOUND\_CATABOLIC\_PROCESS | Details ... | 28 | 0.42 | 1.48 | 0.036 | 0.379 | 1.000 | 1955 | tags=43%, list=15%, signal=50% |
| 85 | GOBP\_ESTABLISHMENT\_OF\_PIGMENT\_GRANULE\_LOCALIZATION | Details ... | 18 | 0.48 | 1.48 | 0.043 | 0.376 | 1.000 | 3330 | tags=50%, list=26%, signal=67% |
| 86 | GOBP\_MITOTIC\_CYTOKINETIC\_PROCESS | Details ... | 18 | 0.47 | 1.48 | 0.054 | 0.372 | 1.000 | 2783 | tags=44%, list=22%, signal=57% |
| 87 | GOBP\_RELEASE\_OF\_CYTOCHROME\_C\_FROM\_MITOCHONDRIA | Details ... | 52 | 0.37 | 1.47 | 0.026 | 0.386 | 1.000 | 2286 | tags=31%, list=18%, signal=37% |
| 88 | GOBP\_MITOTIC\_CYTOKINESIS | Details ... | 57 | 0.35 | 1.46 | 0.023 | 0.417 | 1.000 | 3556 | tags=46%, list=28%, signal=63% |
| 89 | GOBP\_AUTOPHAGOSOME\_MATURATION | Details ... | 42 | 0.38 | 1.46 | 0.034 | 0.421 | 1.000 | 1710 | tags=31%, list=13%, signal=36% |
| 90 | GOBP\_BASE\_EXCISION\_REPAIR | Details ... | 40 | 0.38 | 1.46 | 0.039 | 0.418 | 1.000 | 1662 | tags=30%, list=13%, signal=34% |
| 91 | GOBP\_PIGMENT\_GRANULE\_LOCALIZATION | Details ... | 20 | 0.45 | 1.45 | 0.054 | 0.425 | 1.000 | 3330 | tags=45%, list=26%, signal=61% |
| 92 | GOBP\_PURINE\_CONTAINING\_COMPOUND\_BIOSYNTHETIC\_PROCESS | Details ... | 169 | 0.29 | 1.45 | 0.007 | 0.420 | 1.000 | 3024 | tags=36%, list=23%, signal=47% |
| 93 | GOBP\_MULTIVESICULAR\_BODY\_SORTING\_PATHWAY | Details ... | 27 | 0.41 | 1.44 | 0.046 | 0.435 | 1.000 | 3518 | tags=56%, list=27%, signal=76% |
| 94 | GOBP\_B\_CELL\_MEDIATED\_IMMUNITY | Details ... | 148 | 0.29 | 1.44 | 0.009 | 0.444 | 1.000 | 3449 | tags=41%, list=27%, signal=55% |
| 95 | GOBP\_POSITIVE\_REGULATION\_OF\_TOR\_SIGNALING | Details ... | 34 | 0.38 | 1.44 | 0.055 | 0.450 | 1.000 | 2674 | tags=44%, list=21%, signal=56% |
| 96 | GOBP\_CYTOSKELETON\_DEPENDENT\_CYTOKINESIS | Details ... | 76 | 0.33 | 1.43 | 0.018 | 0.451 | 1.000 | 2806 | tags=34%, list=22%, signal=43% |
| 97 | GOBP\_NUCLEOSIDE\_TRIPHOSPHATE\_METABOLIC\_PROCESS | Details ... | 86 | 0.31 | 1.43 | 0.018 | 0.446 | 1.000 | 3024 | tags=36%, list=23%, signal=47% |
| 98 | GOBP\_POSITIVE\_REGULATION\_OF\_PROTEIN\_DEPOLYMERIZATION | Details ... | 15 | 0.48 | 1.43 | 0.086 | 0.451 | 1.000 | 2532 | tags=40%, list=20%, signal=50% |
| 99 | GOBP\_NUCLEOSIDE\_BISPHOSPHATE\_METABOLIC\_PROCESS | Details ... | 92 | 0.31 | 1.42 | 0.024 | 0.463 | 1.000 | 2567 | tags=34%, list=20%, signal=42% |
| 100 | GOBP\_QUINONE\_METABOLIC\_PROCESS | Details ... | 30 | 0.40 | 1.42 | 0.055 | 0.472 | 1.000 | 2690 | tags=47%, list=21%, signal=59% |
| 101 | GOBP\_THIOESTER\_BIOSYNTHETIC\_PROCESS |  | 39 | 0.37 | 1.42 | 0.062 | 0.477 | 1.000 | 2520 | tags=36%, list=20%, signal=44% |
| 102 | GOBP\_TETRAPYRROLE\_METABOLIC\_PROCESS |  | 53 | 0.35 | 1.41 | 0.033 | 0.480 | 1.000 | 2215 | tags=32%, list=17%, signal=39% |
| 103 | GOBP\_PYRIMIDINE\_NUCLEOSIDE\_TRIPHOSPHATE\_BIOSYNTHETIC\_PROCESS |  | 15 | 0.49 | 1.41 | 0.068 | 0.481 | 1.000 | 1428 | tags=33%, list=11%, signal=37% |
| 104 | GOBP\_THIOESTER\_METABOLIC\_PROCESS |  | 68 | 0.32 | 1.41 | 0.023 | 0.478 | 1.000 | 2124 | tags=31%, list=16%, signal=37% |
| 105 | GOBP\_LIPID\_CATABOLIC\_PROCESS |  | 247 | 0.26 | 1.40 | 0.000 | 0.496 | 1.000 | 2299 | tags=25%, list=18%, signal=30% |
| 106 | GOBP\_PRIMARY\_ALCOHOL\_METABOLIC\_PROCESS |  | 86 | 0.31 | 1.40 | 0.023 | 0.501 | 1.000 | 2895 | tags=34%, list=22%, signal=43% |
| 107 | GOBP\_GLUCOSE\_6\_PHOSPHATE\_METABOLIC\_PROCESS |  | 23 | 0.43 | 1.40 | 0.062 | 0.498 | 1.000 | 976 | tags=26%, list=8%, signal=28% |
| 108 | GOBP\_MITOCHONDRIAL\_TRANSPORT |  | 156 | 0.28 | 1.40 | 0.014 | 0.496 | 1.000 | 2390 | tags=26%, list=19%, signal=32% |
| 109 | GOBP\_DETOXIFICATION |  | 106 | 0.30 | 1.40 | 0.019 | 0.492 | 1.000 | 2776 | tags=32%, list=22%, signal=41% |
| 110 | GOBP\_PLASMA\_MEMBRANE\_REPAIR |  | 18 | 0.45 | 1.40 | 0.083 | 0.499 | 1.000 | 1269 | tags=33%, list=10%, signal=37% |
| 111 | GOBP\_NUCLEOSIDE\_PHOSPHATE\_BIOSYNTHETIC\_PROCESS |  | 205 | 0.27 | 1.39 | 0.003 | 0.498 | 1.000 | 3024 | tags=34%, list=23%, signal=44% |
| 112 | GOBP\_NUCLEOBASE\_CONTAINING\_SMALL\_MOLECULE\_METABOLIC\_PROCESS |  | 453 | 0.25 | 1.39 | 0.000 | 0.497 | 1.000 | 3024 | tags=33%, list=23%, signal=41% |
| 113 | GOBP\_ESTABLISHMENT\_OF\_TISSUE\_POLARITY |  | 55 | 0.33 | 1.39 | 0.058 | 0.499 | 1.000 | 2693 | tags=36%, list=21%, signal=46% |
| 114 | GOBP\_VIRAL\_BUDDING |  | 19 | 0.44 | 1.39 | 0.092 | 0.506 | 1.000 | 3037 | tags=53%, list=24%, signal=69% |
| 115 | GOBP\_PRODUCTION\_OF\_MOLECULAR\_MEDIATOR\_OF\_IMMUNE\_RESPONSE |  | 207 | 0.26 | 1.39 | 0.012 | 0.508 | 1.000 | 2668 | tags=30%, list=21%, signal=38% |
| 116 | GOBP\_GMP\_METABOLIC\_PROCESS |  | 22 | 0.41 | 1.39 | 0.077 | 0.505 | 1.000 | 2867 | tags=45%, list=22%, signal=58% |
| 117 | GOBP\_BRANCHED\_CHAIN\_AMINO\_ACID\_METABOLIC\_PROCESS |  | 22 | 0.42 | 1.38 | 0.079 | 0.502 | 1.000 | 1925 | tags=32%, list=15%, signal=37% |
| 118 | GOBP\_NEGATIVE\_REGULATION\_OF\_INTERLEUKIN\_10\_PRODUCTION |  | 15 | 0.46 | 1.38 | 0.123 | 0.515 | 1.000 | 2332 | tags=40%, list=18%, signal=49% |
| 119 | GOBP\_PORPHYRIN\_CONTAINING\_COMPOUND\_METABOLIC\_PROCESS |  | 43 | 0.35 | 1.38 | 0.057 | 0.519 | 1.000 | 1575 | tags=26%, list=12%, signal=29% |
| 120 | GOBP\_LYTIC\_VACUOLE\_ORGANIZATION |  | 54 | 0.34 | 1.38 | 0.060 | 0.517 | 1.000 | 3418 | tags=44%, list=26%, signal=60% |
| 121 | GOBP\_GLUTAMINE\_FAMILY\_AMINO\_ACID\_BIOSYNTHETIC\_PROCESS |  | 15 | 0.47 | 1.37 | 0.104 | 0.517 | 1.000 | 2628 | tags=60%, list=20%, signal=75% |
| 122 | GOBP\_PROTEIN\_HYDROXYLATION |  | 23 | 0.40 | 1.37 | 0.111 | 0.527 | 1.000 | 1710 | tags=30%, list=13%, signal=35% |
| 123 | GOBP\_ARGININE\_METABOLIC\_PROCESS |  | 18 | 0.44 | 1.37 | 0.117 | 0.523 | 1.000 | 2628 | tags=50%, list=20%, signal=63% |
| 124 | GOBP\_PURINE\_NUCLEOSIDE\_MONOPHOSPHATE\_METABOLIC\_PROCESS |  | 36 | 0.37 | 1.37 | 0.075 | 0.519 | 1.000 | 1852 | tags=31%, list=14%, signal=36% |
| 125 | GOBP\_CYTOKINETIC\_PROCESS |  | 27 | 0.39 | 1.37 | 0.092 | 0.519 | 1.000 | 2783 | tags=41%, list=22%, signal=52% |
| 126 | GOBP\_NEGATIVE\_REGULATION\_OF\_SIGNALING\_RECEPTOR\_ACTIVITY |  | 36 | 0.36 | 1.37 | 0.072 | 0.520 | 1.000 | 809 | tags=22%, list=6%, signal=24% |
| 127 | GOBP\_VACUOLAR\_TRANSPORT |  | 119 | 0.28 | 1.37 | 0.024 | 0.516 | 1.000 | 3074 | tags=37%, list=24%, signal=48% |
| 128 | GOBP\_UBIQUITIN\_DEPENDENT\_PROTEIN\_CATABOLIC\_PROCESS\_VIA\_THE\_MULTIVESICULAR\_BODY\_SORTING\_PATHWAY |  | 25 | 0.40 | 1.36 | 0.079 | 0.522 | 1.000 | 3037 | tags=48%, list=24%, signal=63% |
| 129 | GOBP\_MACROPHAGE\_ACTIVATION\_INVOLVED\_IN\_IMMUNE\_RESPONSE |  | 15 | 0.45 | 1.36 | 0.102 | 0.521 | 1.000 | 725 | tags=27%, list=6%, signal=28% |
| 130 | GOBP\_REGULATION\_OF\_MITOTIC\_SISTER\_CHROMATID\_SEGREGATION |  | 42 | 0.36 | 1.36 | 0.071 | 0.520 | 1.000 | 3773 | tags=48%, list=29%, signal=67% |
| 131 | GOBP\_ANTIGEN\_RECEPTOR\_MEDIATED\_SIGNALING\_PATHWAY |  | 164 | 0.27 | 1.36 | 0.012 | 0.520 | 1.000 | 3461 | tags=38%, list=27%, signal=52% |
| 132 | GOBP\_MITOCHONDRIAL\_TRANSMEMBRANE\_TRANSPORT |  | 74 | 0.31 | 1.36 | 0.034 | 0.520 | 1.000 | 1872 | tags=24%, list=15%, signal=28% |
| 133 | GOBP\_DNA\_LIGATION |  | 15 | 0.45 | 1.36 | 0.110 | 0.518 | 1.000 | 2898 | tags=53%, list=22%, signal=69% |
| 134 | GOBP\_GLYCOLIPID\_BIOSYNTHETIC\_PROCESS |  | 56 | 0.32 | 1.36 | 0.050 | 0.518 | 1.000 | 2979 | tags=41%, list=23%, signal=53% |
| 135 | GOBP\_LIPOPROTEIN\_BIOSYNTHETIC\_PROCESS |  | 74 | 0.31 | 1.36 | 0.060 | 0.521 | 1.000 | 2801 | tags=38%, list=22%, signal=48% |
| 136 | GOBP\_PEROXISOMAL\_TRANSPORT |  | 21 | 0.42 | 1.35 | 0.117 | 0.526 | 1.000 | 467 | tags=19%, list=4%, signal=20% |
| 137 | GOBP\_REGULATION\_OF\_ESTABLISHMENT\_OF\_PLANAR\_POLARITY |  | 42 | 0.34 | 1.35 | 0.080 | 0.525 | 1.000 | 2693 | tags=38%, list=21%, signal=48% |
| 138 | GOBP\_PURINE\_CONTAINING\_COMPOUND\_METABOLIC\_PROCESS |  | 337 | 0.25 | 1.35 | 0.008 | 0.521 | 1.000 | 3047 | tags=34%, list=24%, signal=44% |
| 139 | GOBP\_CELLULAR\_OXIDANT\_DETOXIFICATION |  | 74 | 0.30 | 1.35 | 0.041 | 0.521 | 1.000 | 2161 | tags=30%, list=17%, signal=36% |
| 140 | GOBP\_CELLULAR\_AMINO\_ACID\_METABOLIC\_PROCESS |  | 231 | 0.26 | 1.35 | 0.020 | 0.525 | 1.000 | 2635 | tags=29%, list=20%, signal=36% |
| 141 | GOBP\_APOPTOTIC\_MITOCHONDRIAL\_CHANGES |  | 87 | 0.30 | 1.35 | 0.038 | 0.527 | 1.000 | 2781 | tags=31%, list=22%, signal=39% |
| 142 | GOBP\_ORGANIC\_ACID\_BIOSYNTHETIC\_PROCESS |  | 249 | 0.25 | 1.34 | 0.011 | 0.526 | 1.000 | 2673 | tags=27%, list=21%, signal=34% |
| 143 | GOBP\_RIBONUCLEOSIDE\_METABOLIC\_PROCESS |  | 28 | 0.39 | 1.34 | 0.088 | 0.535 | 1.000 | 1955 | tags=36%, list=15%, signal=42% |
| 144 | GOBP\_ENDOSOME\_ORGANIZATION |  | 58 | 0.32 | 1.34 | 0.055 | 0.540 | 1.000 | 3037 | tags=33%, list=24%, signal=43% |
| 145 | GOBP\_POSITIVE\_REGULATION\_OF\_MITOCHONDRION\_ORGANIZATION |  | 59 | 0.32 | 1.34 | 0.074 | 0.540 | 1.000 | 2497 | tags=36%, list=19%, signal=44% |
| 146 | GOBP\_NEGATIVE\_REGULATION\_OF\_CYTOKINE\_PRODUCTION\_INVOLVED\_IN\_INFLAMMATORY\_RESPONSE |  | 21 | 0.40 | 1.34 | 0.117 | 0.539 | 1.000 | 3949 | tags=57%, list=31%, signal=82% |
| 147 | GOBP\_ESTABLISHMENT\_OF\_PROTEIN\_LOCALIZATION\_TO\_MEMBRANE |  | 198 | 0.25 | 1.33 | 0.010 | 0.547 | 1.000 | 1883 | tags=22%, list=15%, signal=26% |
| 148 | GOBP\_INNER\_MITOCHONDRIAL\_MEMBRANE\_ORGANIZATION |  | 29 | 0.38 | 1.33 | 0.106 | 0.544 | 1.000 | 2279 | tags=34%, list=18%, signal=42% |
| 149 | GOBP\_ORGANISM\_EMERGENCE\_FROM\_PROTECTIVE\_STRUCTURE |  | 15 | 0.44 | 1.33 | 0.129 | 0.542 | 1.000 | 1302 | tags=27%, list=10%, signal=30% |
| 150 | GOBP\_MULTIVESICULAR\_BODY\_ORGANIZATION |  | 23 | 0.39 | 1.33 | 0.117 | 0.552 | 1.000 | 3037 | tags=48%, list=24%, signal=62% |
| 151 | GOBP\_TRNA\_METABOLIC\_PROCESS |  | 130 | 0.27 | 1.33 | 0.041 | 0.554 | 1.000 | 2009 | tags=25%, list=16%, signal=29% |
| 152 | GOBP\_VACUOLE\_ORGANIZATION |  | 143 | 0.27 | 1.32 | 0.032 | 0.559 | 1.000 | 2962 | tags=34%, list=23%, signal=43% |
| 153 | GOBP\_RESPONSE\_TO\_TOXIC\_SUBSTANCE |  | 198 | 0.25 | 1.32 | 0.021 | 0.557 | 1.000 | 2589 | tags=28%, list=20%, signal=34% |
| 154 | GOBP\_REGULATION\_OF\_REACTIVE\_OXYGEN\_SPECIES\_METABOLIC\_PROCESS |  | 123 | 0.27 | 1.32 | 0.037 | 0.564 | 1.000 | 2580 | tags=28%, list=20%, signal=34% |
| 155 | GOBP\_NEGATIVE\_REGULATION\_OF\_MYOBLAST\_DIFFERENTIATION |  | 18 | 0.41 | 1.32 | 0.139 | 0.566 | 1.000 | 2490 | tags=50%, list=19%, signal=62% |
| 156 | GOBP\_RIBONUCLEOSIDE\_TRIPHOSPHATE\_METABOLIC\_PROCESS |  | 71 | 0.30 | 1.32 | 0.061 | 0.566 | 1.000 | 3024 | tags=39%, list=23%, signal=51% |
| 157 | GOBP\_AXONAL\_TRANSPORT\_OF\_MITOCHONDRION |  | 16 | 0.43 | 1.32 | 0.146 | 0.564 | 1.000 | 1658 | tags=38%, list=13%, signal=43% |
| 158 | GOBP\_PROCESS\_UTILIZING\_AUTOPHAGIC\_MECHANISM |  | 423 | 0.23 | 1.31 | 0.008 | 0.570 | 1.000 | 2140 | tags=24%, list=17%, signal=27% |
| 159 | GOBP\_TOLL\_LIKE\_RECEPTOR\_3\_SIGNALING\_PATHWAY |  | 15 | 0.44 | 1.31 | 0.150 | 0.567 | 1.000 | 740 | tags=27%, list=6%, signal=28% |
| 160 | GOBP\_RESPONSE\_TO\_INCREASED\_OXYGEN\_LEVELS |  | 23 | 0.39 | 1.31 | 0.123 | 0.589 | 1.000 | 2387 | tags=30%, list=19%, signal=37% |
| 161 | GOBP\_NEGATIVE\_REGULATION\_OF\_METAPHASE\_ANAPHASE\_TRANSITION\_OF\_CELL\_CYCLE |  | 38 | 0.35 | 1.30 | 0.098 | 0.595 | 1.000 | 3773 | tags=47%, list=29%, signal=67% |
| 162 | GOBP\_REGULATION\_OF\_MACROPHAGE\_MIGRATION |  | 35 | 0.35 | 1.30 | 0.113 | 0.597 | 1.000 | 1933 | tags=29%, list=15%, signal=34% |
| 163 | GOBP\_MALE\_MEIOSIS\_I |  | 20 | 0.40 | 1.30 | 0.150 | 0.602 | 1.000 | 4184 | tags=50%, list=32%, signal=74% |
| 164 | GOBP\_CELLULAR\_LIPID\_CATABOLIC\_PROCESS |  | 166 | 0.26 | 1.30 | 0.054 | 0.599 | 1.000 | 3327 | tags=36%, list=26%, signal=47% |
| 165 | GOBP\_CELLULAR\_MODIFIED\_AMINO\_ACID\_BIOSYNTHETIC\_PROCESS |  | 36 | 0.34 | 1.30 | 0.120 | 0.596 | 1.000 | 2795 | tags=36%, list=22%, signal=46% |
| 166 | GOBP\_NEUROTRANSMITTER\_METABOLIC\_PROCESS |  | 24 | 0.37 | 1.30 | 0.136 | 0.593 | 1.000 | 2795 | tags=38%, list=22%, signal=48% |
| 167 | GOBP\_STEROID\_CATABOLIC\_PROCESS |  | 26 | 0.37 | 1.30 | 0.145 | 0.595 | 1.000 | 3000 | tags=38%, list=23%, signal=50% |
| 168 | GOBP\_COMPLEMENT\_ACTIVATION |  | 73 | 0.30 | 1.29 | 0.044 | 0.595 | 1.000 | 3449 | tags=44%, list=27%, signal=59% |
| 169 | GOBP\_PIGMENT\_BIOSYNTHETIC\_PROCESS |  | 59 | 0.31 | 1.29 | 0.089 | 0.597 | 1.000 | 3727 | tags=44%, list=29%, signal=62% |
| 170 | GOBP\_MANNOSYLATION |  | 20 | 0.40 | 1.29 | 0.148 | 0.601 | 1.000 | 2663 | tags=30%, list=21%, signal=38% |
| 171 | GOBP\_RESPONSE\_TO\_MAGNESIUM\_ION |  | 15 | 0.43 | 1.29 | 0.127 | 0.598 | 1.000 | 1928 | tags=33%, list=15%, signal=39% |
| 172 | GOBP\_AMMONIUM\_ION\_METABOLIC\_PROCESS |  | 16 | 0.44 | 1.29 | 0.160 | 0.596 | 1.000 | 2455 | tags=44%, list=19%, signal=54% |
| 173 | GOBP\_CELLULAR\_AMINO\_ACID\_CATABOLIC\_PROCESS |  | 91 | 0.28 | 1.29 | 0.079 | 0.595 | 1.000 | 2635 | tags=35%, list=20%, signal=44% |
| 174 | GOBP\_CELLULAR\_ALDEHYDE\_METABOLIC\_PROCESS |  | 52 | 0.32 | 1.29 | 0.080 | 0.593 | 1.000 | 2895 | tags=38%, list=22%, signal=49% |
| 175 | GOBP\_RIBOSE\_PHOSPHATE\_METABOLIC\_PROCESS |  | 321 | 0.23 | 1.29 | 0.000 | 0.592 | 1.000 | 3047 | tags=33%, list=24%, signal=42% |
| 176 | GOBP\_ANTIGEN\_PROCESSING\_AND\_PRESENTATION |  | 97 | 0.28 | 1.29 | 0.059 | 0.590 | 1.000 | 2845 | tags=33%, list=22%, signal=42% |
| 177 | GOBP\_PEPTIDYL\_GLUTAMIC\_ACID\_MODIFICATION |  | 19 | 0.39 | 1.29 | 0.129 | 0.590 | 1.000 | 3620 | tags=58%, list=28%, signal=80% |
| 178 | GOBP\_DOPAMINE\_METABOLIC\_PROCESS |  | 38 | 0.34 | 1.29 | 0.110 | 0.588 | 1.000 | 2192 | tags=29%, list=17%, signal=35% |
| 179 | GOBP\_CARBOHYDRATE\_DERIVATIVE\_CATABOLIC\_PROCESS |  | 135 | 0.26 | 1.28 | 0.038 | 0.597 | 1.000 | 2398 | tags=29%, list=19%, signal=35% |
| 180 | GOBP\_REGULATION\_OF\_AUTOPHAGY |  | 273 | 0.24 | 1.28 | 0.003 | 0.598 | 1.000 | 2134 | tags=25%, list=17%, signal=29% |
| 181 | GOBP\_SULFUR\_COMPOUND\_BIOSYNTHETIC\_PROCESS |  | 120 | 0.26 | 1.28 | 0.054 | 0.597 | 1.000 | 2635 | tags=30%, list=20%, signal=37% |
| 182 | GOBP\_NEGATIVE\_REGULATION\_OF\_NEURAL\_PRECURSOR\_CELL\_PROLIFERATION |  | 18 | 0.41 | 1.28 | 0.141 | 0.594 | 1.000 | 1995 | tags=33%, list=15%, signal=39% |
| 183 | GOBP\_MEMBRANE\_LIPID\_METABOLIC\_PROCESS |  | 160 | 0.26 | 1.28 | 0.063 | 0.596 | 1.000 | 2663 | tags=31%, list=21%, signal=38% |
| 184 | GOBP\_ORGANIC\_ACID\_CATABOLIC\_PROCESS |  | 197 | 0.25 | 1.28 | 0.044 | 0.596 | 1.000 | 2635 | tags=29%, list=20%, signal=36% |
| 185 | GOBP\_BRANCHED\_CHAIN\_AMINO\_ACID\_CATABOLIC\_PROCESS |  | 19 | 0.40 | 1.28 | 0.143 | 0.594 | 1.000 | 3552 | tags=47%, list=28%, signal=65% |
| 186 | GOBP\_PYRIMIDINE\_RIBONUCLEOTIDE\_BIOSYNTHETIC\_PROCESS |  | 18 | 0.41 | 1.27 | 0.161 | 0.604 | 1.000 | 1428 | tags=28%, list=11%, signal=31% |
| 187 | GOBP\_BENZENE\_CONTAINING\_COMPOUND\_METABOLIC\_PROCESS |  | 22 | 0.38 | 1.27 | 0.147 | 0.603 | 1.000 | 3209 | tags=41%, list=25%, signal=54% |
| 188 | GOBP\_NEGATIVE\_REGULATION\_OF\_CHROMOSOME\_ORGANIZATION |  | 78 | 0.29 | 1.27 | 0.097 | 0.604 | 1.000 | 3773 | tags=47%, list=29%, signal=67% |
| 189 | GOBP\_MAINTENANCE\_OF\_PROTEIN\_LOCATION |  | 79 | 0.28 | 1.27 | 0.082 | 0.606 | 1.000 | 1486 | tags=19%, list=12%, signal=21% |
| 190 | GOBP\_REGULATION\_OF\_SUPEROXIDE\_ANION\_GENERATION |  | 21 | 0.38 | 1.27 | 0.150 | 0.604 | 1.000 | 2490 | tags=33%, list=19%, signal=41% |
| 191 | GOBP\_POSITIVE\_REGULATION\_OF\_ACTIVATED\_T\_CELL\_PROLIFERATION |  | 23 | 0.38 | 1.27 | 0.140 | 0.604 | 1.000 | 2472 | tags=35%, list=19%, signal=43% |
| 192 | GOBP\_POSITIVE\_REGULATION\_OF\_PROTEIN\_CONTAINING\_COMPLEX\_DISASSEMBLY |  | 28 | 0.36 | 1.27 | 0.124 | 0.605 | 1.000 | 2532 | tags=39%, list=20%, signal=49% |
| 193 | GOBP\_MAINTENANCE\_OF\_PROTEIN\_LOCATION\_IN\_CELL |  | 57 | 0.31 | 1.27 | 0.100 | 0.603 | 1.000 | 2227 | tags=25%, list=17%, signal=30% |
| 194 | GOBP\_GLYCOPROTEIN\_BIOSYNTHETIC\_PROCESS |  | 220 | 0.24 | 1.27 | 0.032 | 0.601 | 1.000 | 1691 | tags=19%, list=13%, signal=22% |
| 195 | GOBP\_GTP\_METABOLIC\_PROCESS |  | 20 | 0.39 | 1.27 | 0.129 | 0.603 | 1.000 | 1428 | tags=30%, list=11%, signal=34% |
| 196 | GOBP\_NON\_CANONICAL\_WNT\_SIGNALING\_PATHWAY |  | 56 | 0.30 | 1.26 | 0.110 | 0.615 | 1.000 | 2693 | tags=34%, list=21%, signal=43% |
| 197 | GOBP\_POSITIVE\_REGULATION\_OF\_B\_CELL\_ACTIVATION |  | 83 | 0.28 | 1.26 | 0.087 | 0.612 | 1.000 | 3449 | tags=42%, list=27%, signal=57% |
| 198 | GOBP\_POSITIVE\_REGULATION\_OF\_CALCIUM\_ION\_TRANSMEMBRANE\_TRANSPORTER\_ACTIVITY |  | 32 | 0.35 | 1.26 | 0.142 | 0.621 | 1.000 | 3400 | tags=41%, list=26%, signal=55% |
| 199 | GOBP\_POSITIVE\_REGULATION\_OF\_MACROPHAGE\_MIGRATION |  | 22 | 0.38 | 1.26 | 0.163 | 0.620 | 1.000 | 1933 | tags=32%, list=15%, signal=37% |
| 200 | GOBP\_POSITIVE\_REGULATION\_OF\_PROTEOLYSIS\_INVOLVED\_IN\_CELLULAR\_PROTEIN\_CATABOLIC\_PROCESS |  | 108 | 0.27 | 1.26 | 0.081 | 0.621 | 1.000 | 2399 | tags=29%, list=19%, signal=35% |
| 201 | GOBP\_DNA\_REPLICATION\_INITIATION |  | 34 | 0.33 | 1.26 | 0.138 | 0.618 | 1.000 | 1410 | tags=26%, list=11%, signal=30% |
| 202 | GOBP\_PYRIMIDINE\_NUCLEOTIDE\_METABOLIC\_PROCESS |  | 42 | 0.32 | 1.25 | 0.128 | 0.624 | 1.000 | 1428 | tags=24%, list=11%, signal=27% |
| 203 | GOBP\_METAPHASE\_PLATE\_CONGRESSION |  | 55 | 0.31 | 1.25 | 0.139 | 0.623 | 1.000 | 3729 | tags=38%, list=29%, signal=53% |
| 204 | GOBP\_MITOTIC\_METAPHASE\_PLATE\_CONGRESSION |  | 45 | 0.32 | 1.25 | 0.133 | 0.620 | 1.000 | 3729 | tags=40%, list=29%, signal=56% |
| 205 | GOBP\_REGULATION\_OF\_VACUOLE\_ORGANIZATION |  | 42 | 0.33 | 1.25 | 0.162 | 0.618 | 1.000 | 3418 | tags=45%, list=26%, signal=61% |
| 206 | GOBP\_METAPHASE\_ANAPHASE\_TRANSITION\_OF\_CELL\_CYCLE |  | 52 | 0.31 | 1.25 | 0.128 | 0.615 | 1.000 | 3773 | tags=44%, list=29%, signal=62% |
| 207 | GOBP\_MITOCHONDRIAL\_CALCIUM\_ION\_HOMEOSTASIS |  | 19 | 0.39 | 1.25 | 0.179 | 0.613 | 1.000 | 2069 | tags=32%, list=16%, signal=38% |
| 208 | GOBP\_REGULATION\_OF\_MITOCHONDRION\_ORGANIZATION |  | 120 | 0.26 | 1.25 | 0.086 | 0.614 | 1.000 | 2580 | tags=28%, list=20%, signal=34% |
| 209 | GOBP\_RESPIRATORY\_BURST |  | 31 | 0.34 | 1.25 | 0.150 | 0.614 | 1.000 | 2195 | tags=29%, list=17%, signal=35% |
| 210 | GOBP\_REGULATION\_OF\_MITOCHONDRIAL\_GENE\_EXPRESSION |  | 18 | 0.40 | 1.25 | 0.173 | 0.612 | 1.000 | 1559 | tags=33%, list=12%, signal=38% |
| 211 | GOBP\_TERPENOID\_BIOSYNTHETIC\_PROCESS |  | 16 | 0.42 | 1.25 | 0.178 | 0.614 | 1.000 | 3058 | tags=50%, list=24%, signal=65% |
| 212 | GOBP\_PYRIMIDINE\_CONTAINING\_COMPOUND\_BIOSYNTHETIC\_PROCESS |  | 29 | 0.35 | 1.24 | 0.147 | 0.628 | 1.000 | 1428 | tags=24%, list=11%, signal=27% |
| 213 | GOBP\_POSITIVE\_REGULATION\_OF\_PROTEASOMAL\_UBIQUITIN\_DEPENDENT\_PROTEIN\_CATABOLIC\_PROCESS |  | 72 | 0.29 | 1.24 | 0.098 | 0.626 | 1.000 | 2332 | tags=29%, list=18%, signal=35% |
| 214 | GOBP\_PHAGOCYTOSIS\_RECOGNITION |  | 45 | 0.31 | 1.24 | 0.135 | 0.625 | 1.000 | 2798 | tags=36%, list=22%, signal=45% |
| 215 | GOBP\_REGULATION\_OF\_AMYLOID\_FIBRIL\_FORMATION |  | 15 | 0.42 | 1.24 | 0.188 | 0.625 | 1.000 | 3192 | tags=47%, list=25%, signal=62% |
| 216 | GOBP\_PEPTIDE\_CATABOLIC\_PROCESS |  | 23 | 0.37 | 1.24 | 0.175 | 0.632 | 1.000 | 634 | tags=17%, list=5%, signal=18% |
| 217 | GOBP\_SKELETAL\_MUSCLE\_CONTRACTION |  | 32 | 0.34 | 1.24 | 0.144 | 0.630 | 1.000 | 3490 | tags=44%, list=27%, signal=60% |
| 218 | GOBP\_RNA\_5\_END\_PROCESSING |  | 19 | 0.40 | 1.24 | 0.192 | 0.632 | 1.000 | 1337 | tags=32%, list=10%, signal=35% |
| 219 | GOBP\_ALDITOL\_METABOLIC\_PROCESS |  | 16 | 0.41 | 1.24 | 0.205 | 0.630 | 1.000 | 3042 | tags=38%, list=24%, signal=49% |
| 220 | GOBP\_REGULATION\_OF\_SYNAPTIC\_VESICLE\_RECYCLING |  | 15 | 0.42 | 1.24 | 0.186 | 0.628 | 1.000 | 3400 | tags=47%, list=26%, signal=63% |
| 221 | GOBP\_MITOTIC\_SPINDLE\_ASSEMBLY |  | 61 | 0.29 | 1.24 | 0.107 | 0.630 | 1.000 | 3590 | tags=36%, list=28%, signal=50% |
| 222 | GOBP\_REGULATION\_OF\_CHROMOSOME\_SEPARATION |  | 59 | 0.29 | 1.23 | 0.114 | 0.634 | 1.000 | 3773 | tags=42%, list=29%, signal=60% |
| 223 | GOBP\_PROTEIN\_TARGETING\_TO\_MITOCHONDRION |  | 80 | 0.28 | 1.23 | 0.108 | 0.632 | 1.000 | 2390 | tags=28%, list=19%, signal=34% |
| 224 | GOBP\_NUCLEOSIDE\_MONOPHOSPHATE\_METABOLIC\_PROCESS |  | 62 | 0.29 | 1.23 | 0.110 | 0.630 | 1.000 | 1852 | tags=23%, list=14%, signal=26% |
| 225 | GOBP\_MITOCHONDRIAL\_FISSION |  | 29 | 0.35 | 1.23 | 0.183 | 0.628 | 1.000 | 2150 | tags=34%, list=17%, signal=41% |
| 226 | GOBP\_PROTEIN\_TARGETING |  | 244 | 0.23 | 1.23 | 0.025 | 0.629 | 1.000 | 2390 | tags=25%, list=19%, signal=30% |
| 227 | GOBP\_POSITIVE\_REGULATION\_OF\_VACUOLE\_ORGANIZATION |  | 18 | 0.39 | 1.23 | 0.197 | 0.628 | 1.000 | 1766 | tags=33%, list=14%, signal=39% |
| 228 | GOBP\_RESPIRATORY\_SYSTEM\_PROCESS |  | 29 | 0.35 | 1.23 | 0.185 | 0.636 | 1.000 | 1883 | tags=31%, list=15%, signal=36% |
| 229 | GOBP\_CELL\_REDOX\_HOMEOSTASIS |  | 31 | 0.33 | 1.23 | 0.165 | 0.638 | 1.000 | 2161 | tags=29%, list=17%, signal=35% |
| 230 | GOBP\_CALCIUM\_DEPENDENT\_CELL\_CELL\_ADHESION\_VIA\_PLASMA\_MEMBRANE\_CELL\_ADHESION\_MOLECULES |  | 30 | 0.33 | 1.23 | 0.161 | 0.639 | 1.000 | 4632 | tags=63%, list=36%, signal=99% |
| 231 | GOBP\_PHOSPHOLIPID\_CATABOLIC\_PROCESS |  | 35 | 0.33 | 1.23 | 0.162 | 0.636 | 1.000 | 1855 | tags=29%, list=14%, signal=33% |
| 232 | GOBP\_ICOSANOID\_BIOSYNTHETIC\_PROCESS |  | 42 | 0.32 | 1.23 | 0.157 | 0.634 | 1.000 | 610 | tags=17%, list=5%, signal=17% |
| 233 | GOBP\_GROOMING\_BEHAVIOR |  | 15 | 0.41 | 1.22 | 0.192 | 0.635 | 1.000 | 1839 | tags=33%, list=14%, signal=39% |
| 234 | GOBP\_ISOPRENOID\_BIOSYNTHETIC\_PROCESS |  | 27 | 0.34 | 1.22 | 0.185 | 0.635 | 1.000 | 3058 | tags=41%, list=24%, signal=53% |
| 235 | GOBP\_ADAPTIVE\_IMMUNE\_RESPONSE |  | 418 | 0.22 | 1.22 | 0.030 | 0.634 | 1.000 | 2967 | tags=31%, list=23%, signal=39% |
| 236 | GOBP\_LYMPHOCYTE\_MEDIATED\_IMMUNITY |  | 271 | 0.23 | 1.22 | 0.034 | 0.642 | 1.000 | 3449 | tags=37%, list=27%, signal=50% |
| 237 | GOBP\_SMALL\_MOLECULE\_CATABOLIC\_PROCESS |  | 311 | 0.22 | 1.22 | 0.025 | 0.641 | 1.000 | 3218 | tags=33%, list=25%, signal=43% |
| 238 | GOBP\_REGULATION\_OF\_MACROAUTOPHAGY |  | 127 | 0.25 | 1.22 | 0.105 | 0.643 | 1.000 | 1766 | tags=21%, list=14%, signal=24% |
| 239 | GOBP\_PYROPTOSIS |  | 17 | 0.39 | 1.22 | 0.185 | 0.649 | 1.000 | 2427 | tags=41%, list=19%, signal=51% |
| 240 | GOBP\_ALPHA\_AMINO\_ACID\_CATABOLIC\_PROCESS |  | 77 | 0.27 | 1.22 | 0.117 | 0.647 | 1.000 | 2635 | tags=34%, list=20%, signal=42% |
| 241 | GOBP\_FATTY\_ACID\_CATABOLIC\_PROCESS |  | 84 | 0.27 | 1.21 | 0.132 | 0.649 | 1.000 | 1925 | tags=24%, list=15%, signal=28% |
| 242 | GOBP\_IRON\_SULFUR\_CLUSTER\_ASSEMBLY |  | 20 | 0.37 | 1.21 | 0.176 | 0.661 | 1.000 | 4161 | tags=65%, list=32%, signal=96% |
| 243 | GOBP\_NUCLEAR\_MEMBRANE\_REASSEMBLY |  | 19 | 0.38 | 1.21 | 0.214 | 0.659 | 1.000 | 1502 | tags=37%, list=12%, signal=42% |
| 244 | GOBP\_ORGANELLE\_FUSION |  | 114 | 0.26 | 1.21 | 0.125 | 0.663 | 1.000 | 2901 | tags=29%, list=22%, signal=37% |
| 245 | GOBP\_REGULATION\_OF\_MITOCHONDRIAL\_FISSION |  | 23 | 0.36 | 1.21 | 0.201 | 0.665 | 1.000 | 2150 | tags=39%, list=17%, signal=47% |
| 246 | GOBP\_CELLULAR\_RESPONSE\_TO\_INTERFERON\_BETA |  | 18 | 0.38 | 1.20 | 0.221 | 0.673 | 1.000 | 393 | tags=17%, list=3%, signal=17% |
| 247 | GOBP\_INTRINSIC\_APOPTOTIC\_SIGNALING\_PATHWAY\_IN\_RESPONSE\_TO\_OXIDATIVE\_STRESS |  | 41 | 0.31 | 1.20 | 0.190 | 0.676 | 1.000 | 2545 | tags=41%, list=20%, signal=51% |
| 248 | GOBP\_AMINE\_METABOLIC\_PROCESS |  | 99 | 0.25 | 1.20 | 0.124 | 0.685 | 1.000 | 2531 | tags=26%, list=20%, signal=32% |
| 249 | GOBP\_PEPTIDYL\_PROLINE\_MODIFICATION |  | 43 | 0.31 | 1.20 | 0.180 | 0.687 | 1.000 | 2304 | tags=35%, list=18%, signal=42% |
| 250 | GOBP\_ETHER\_METABOLIC\_PROCESS |  | 21 | 0.37 | 1.20 | 0.205 | 0.690 | 1.000 | 1874 | tags=33%, list=15%, signal=39% |
| 251 | GOBP\_MONOCARBOXYLIC\_ACID\_BIOSYNTHETIC\_PROCESS |  | 169 | 0.24 | 1.19 | 0.099 | 0.696 | 1.000 | 2017 | tags=21%, list=16%, signal=24% |
| 252 | GOBP\_ACETYL\_COA\_METABOLIC\_PROCESS |  | 27 | 0.35 | 1.19 | 0.215 | 0.699 | 1.000 | 2433 | tags=41%, list=19%, signal=50% |
| 253 | GOBP\_REGULATION\_OF\_CATECHOLAMINE\_METABOLIC\_PROCESS |  | 19 | 0.37 | 1.19 | 0.228 | 0.699 | 1.000 | 2192 | tags=37%, list=17%, signal=44% |
| 254 | GOBP\_AMINE\_CATABOLIC\_PROCESS |  | 20 | 0.37 | 1.19 | 0.213 | 0.697 | 1.000 | 2502 | tags=40%, list=19%, signal=50% |
| 255 | GOBP\_SIGNAL\_TRANSDUCTION\_INVOLVED\_IN\_REGULATION\_OF\_GENE\_EXPRESSION |  | 19 | 0.37 | 1.19 | 0.212 | 0.696 | 1.000 | 1008 | tags=21%, list=8%, signal=23% |
| 256 | GOBP\_GLYCOPROTEIN\_METABOLIC\_PROCESS |  | 276 | 0.22 | 1.19 | 0.073 | 0.694 | 1.000 | 2648 | tags=26%, list=21%, signal=32% |
| 257 | GOBP\_NEGATIVE\_REGULATION\_OF\_CATION\_CHANNEL\_ACTIVITY |  | 36 | 0.31 | 1.19 | 0.205 | 0.693 | 1.000 | 4133 | tags=44%, list=32%, signal=65% |
| 258 | GOBP\_REGULATION\_OF\_PROTEIN\_KINASE\_A\_SIGNALING |  | 16 | 0.39 | 1.19 | 0.249 | 0.691 | 1.000 | 1963 | tags=31%, list=15%, signal=37% |
| 259 | GOBP\_POSITIVE\_REGULATION\_OF\_ATP\_DEPENDENT\_ACTIVITY |  | 42 | 0.31 | 1.19 | 0.203 | 0.691 | 1.000 | 1863 | tags=29%, list=14%, signal=33% |
| 260 | GOBP\_REGULATION\_OF\_TOLL\_LIKE\_RECEPTOR\_4\_SIGNALING\_PATHWAY |  | 17 | 0.39 | 1.19 | 0.225 | 0.690 | 1.000 | 1728 | tags=35%, list=13%, signal=41% |
| 261 | GOBP\_GLYCOSPHINGOLIPID\_METABOLIC\_PROCESS |  | 51 | 0.29 | 1.19 | 0.170 | 0.690 | 1.000 | 3025 | tags=37%, list=23%, signal=48% |
| 262 | GOBP\_GLUTAMINE\_FAMILY\_AMINO\_ACID\_METABOLIC\_PROCESS |  | 64 | 0.28 | 1.19 | 0.166 | 0.688 | 1.000 | 2628 | tags=34%, list=20%, signal=43% |
| 263 | GOBP\_CELLULAR\_POLYSACCHARIDE\_CATABOLIC\_PROCESS |  | 16 | 0.40 | 1.19 | 0.234 | 0.686 | 1.000 | 3978 | tags=63%, list=31%, signal=90% |
| 264 | GOBP\_NON\_MOTILE\_CILIUM\_ASSEMBLY |  | 43 | 0.30 | 1.19 | 0.174 | 0.684 | 1.000 | 4186 | tags=47%, list=32%, signal=69% |
| 265 | GOBP\_PROTEIN\_MONOUBIQUITINATION |  | 60 | 0.28 | 1.19 | 0.145 | 0.685 | 1.000 | 3790 | tags=40%, list=29%, signal=56% |
| 266 | GOBP\_GOLGI\_ORGANIZATION |  | 114 | 0.25 | 1.19 | 0.125 | 0.683 | 1.000 | 2256 | tags=24%, list=17%, signal=28% |
| 267 | GOBP\_DEOXYRIBOSE\_PHOSPHATE\_CATABOLIC\_PROCESS |  | 23 | 0.34 | 1.19 | 0.227 | 0.681 | 1.000 | 1068 | tags=22%, list=8%, signal=24% |
| 268 | GOBP\_REGULATION\_OF\_NON\_CANONICAL\_WNT\_SIGNALING\_PATHWAY |  | 18 | 0.38 | 1.18 | 0.226 | 0.682 | 1.000 | 2503 | tags=33%, list=19%, signal=41% |
| 269 | GOBP\_NEGATIVE\_REGULATION\_OF\_NUCLEAR\_DIVISION |  | 47 | 0.30 | 1.18 | 0.199 | 0.682 | 1.000 | 3773 | tags=47%, list=29%, signal=66% |
| 270 | GOBP\_CELL\_RECOGNITION |  | 141 | 0.24 | 1.18 | 0.121 | 0.688 | 1.000 | 3464 | tags=35%, list=27%, signal=47% |
| 271 | GOBP\_POSITIVE\_REGULATION\_OF\_PHAGOCYTOSIS |  | 61 | 0.28 | 1.18 | 0.162 | 0.689 | 1.000 | 2764 | tags=34%, list=21%, signal=44% |
| 272 | GOBP\_ENDOMEMBRANE\_SYSTEM\_ORGANIZATION |  | 389 | 0.21 | 1.18 | 0.048 | 0.691 | 1.000 | 2901 | tags=27%, list=22%, signal=34% |
| 273 | GOBP\_PURINE\_NUCLEOSIDE\_MONOPHOSPHATE\_BIOSYNTHETIC\_PROCESS |  | 21 | 0.36 | 1.18 | 0.249 | 0.688 | 1.000 | 1789 | tags=29%, list=14%, signal=33% |
| 274 | GOBP\_POSITIVE\_REGULATION\_OF\_REACTIVE\_OXYGEN\_SPECIES\_BIOSYNTHETIC\_PROCESS |  | 15 | 0.39 | 1.18 | 0.212 | 0.689 | 1.000 | 1535 | tags=40%, list=12%, signal=45% |
| 275 | GOBP\_POSITIVE\_REGULATION\_OF\_CELLULAR\_PROTEIN\_CATABOLIC\_PROCESS |  | 129 | 0.24 | 1.18 | 0.121 | 0.688 | 1.000 | 2399 | tags=28%, list=19%, signal=34% |
| 276 | GOBP\_PYRIMIDINE\_CONTAINING\_COMPOUND\_METABOLIC\_PROCESS |  | 69 | 0.27 | 1.18 | 0.187 | 0.693 | 1.000 | 1917 | tags=25%, list=15%, signal=29% |
| 277 | GOBP\_CELL\_CELL\_RECOGNITION |  | 48 | 0.29 | 1.18 | 0.185 | 0.692 | 1.000 | 520 | tags=10%, list=4%, signal=11% |
| 278 | GOBP\_POSITIVE\_REGULATION\_OF\_CALCIUM\_MEDIATED\_SIGNALING |  | 30 | 0.33 | 1.17 | 0.207 | 0.692 | 1.000 | 2937 | tags=37%, list=23%, signal=47% |
| 279 | GOBP\_NEGATIVE\_REGULATION\_OF\_OXIDATIVE\_STRESS\_INDUCED\_INTRINSIC\_APOPTOTIC\_SIGNALING\_PATHWAY |  | 19 | 0.37 | 1.17 | 0.244 | 0.693 | 1.000 | 2350 | tags=47%, list=18%, signal=58% |
| 280 | GOBP\_INDOLE\_CONTAINING\_COMPOUND\_METABOLIC\_PROCESS |  | 17 | 0.38 | 1.17 | 0.250 | 0.692 | 1.000 | 3209 | tags=47%, list=25%, signal=63% |
| 281 | GOBP\_REGULATION\_OF\_MITOTIC\_SPINDLE\_ASSEMBLY |  | 20 | 0.37 | 1.17 | 0.249 | 0.690 | 1.000 | 2783 | tags=40%, list=22%, signal=51% |
| 282 | GOBP\_PEPTIDYL\_CYSTEINE\_MODIFICATION |  | 31 | 0.32 | 1.17 | 0.211 | 0.694 | 1.000 | 2370 | tags=35%, list=18%, signal=43% |
| 283 | GOBP\_SYNAPTIC\_TRANSMISSION\_DOPAMINERGIC |  | 24 | 0.34 | 1.17 | 0.210 | 0.693 | 1.000 | 2097 | tags=29%, list=16%, signal=35% |
| 284 | GOBP\_ALPHA\_AMINO\_ACID\_METABOLIC\_PROCESS |  | 165 | 0.23 | 1.17 | 0.089 | 0.690 | 1.000 | 2635 | tags=28%, list=20%, signal=35% |
| 285 | GOBP\_CELLULAR\_CARBOHYDRATE\_CATABOLIC\_PROCESS |  | 35 | 0.31 | 1.17 | 0.217 | 0.694 | 1.000 | 4059 | tags=54%, list=31%, signal=79% |
| 286 | GOBP\_CHROMOSOME\_SEPARATION |  | 74 | 0.26 | 1.17 | 0.184 | 0.699 | 1.000 | 3773 | tags=42%, list=29%, signal=59% |
| 287 | GOBP\_PROTEIN\_DEGLYCOSYLATION |  | 23 | 0.35 | 1.16 | 0.236 | 0.712 | 1.000 | 3581 | tags=48%, list=28%, signal=66% |
| 288 | GOBP\_NADPH\_REGENERATION |  | 16 | 0.38 | 1.16 | 0.240 | 0.712 | 1.000 | 976 | tags=25%, list=8%, signal=27% |
| 289 | GOBP\_REACTIVE\_OXYGEN\_SPECIES\_METABOLIC\_PROCESS |  | 191 | 0.23 | 1.16 | 0.140 | 0.711 | 1.000 | 2490 | tags=26%, list=19%, signal=31% |
| 290 | GOBP\_SKELETAL\_MUSCLE\_ADAPTATION |  | 20 | 0.36 | 1.16 | 0.251 | 0.709 | 1.000 | 3803 | tags=60%, list=29%, signal=85% |
| 291 | GOBP\_POSITIVE\_REGULATION\_OF\_OXIDOREDUCTASE\_ACTIVITY |  | 52 | 0.28 | 1.16 | 0.210 | 0.709 | 1.000 | 2186 | tags=27%, list=17%, signal=32% |
| 292 | GOBP\_DETERMINATION\_OF\_ADULT\_LIFESPAN |  | 16 | 0.38 | 1.16 | 0.256 | 0.711 | 1.000 | 739 | tags=19%, list=6%, signal=20% |
| 293 | GOBP\_SUBSTANTIA\_NIGRA\_DEVELOPMENT |  | 38 | 0.31 | 1.16 | 0.192 | 0.710 | 1.000 | 2192 | tags=32%, list=17%, signal=38% |
| 294 | GOBP\_ANTIGEN\_PROCESSING\_AND\_PRESENTATION\_OF\_PEPTIDE\_ANTIGEN |  | 58 | 0.28 | 1.16 | 0.221 | 0.713 | 1.000 | 2845 | tags=34%, list=22%, signal=44% |
| 295 | GOBP\_MONOCARBOXYLIC\_ACID\_METABOLIC\_PROCESS |  | 470 | 0.21 | 1.16 | 0.073 | 0.712 | 1.000 | 2673 | tags=25%, list=21%, signal=30% |
| 296 | GOBP\_MACROPHAGE\_MIGRATION |  | 46 | 0.29 | 1.16 | 0.201 | 0.710 | 1.000 | 2019 | tags=28%, list=16%, signal=33% |
| 297 | GOBP\_PYRIMIDINE\_RIBONUCLEOTIDE\_METABOLIC\_PROCESS |  | 24 | 0.33 | 1.15 | 0.229 | 0.723 | 1.000 | 1852 | tags=29%, list=14%, signal=34% |
| 298 | GOBP\_BILE\_ACID\_METABOLIC\_PROCESS |  | 39 | 0.30 | 1.15 | 0.221 | 0.722 | 1.000 | 1564 | tags=18%, list=12%, signal=20% |
| 299 | GOBP\_REGULATION\_OF\_HUMORAL\_IMMUNE\_RESPONSE |  | 39 | 0.30 | 1.15 | 0.251 | 0.727 | 1.000 | 3435 | tags=41%, list=27%, signal=56% |
| 300 | GOBP\_REGULATION\_OF\_B\_CELL\_ACTIVATION |  | 123 | 0.23 | 1.15 | 0.165 | 0.739 | 1.000 | 3449 | tags=41%, list=27%, signal=55% |
| 301 | GOBP\_MULTICELLULAR\_ORGANISMAL\_MOVEMENT |  | 44 | 0.29 | 1.15 | 0.233 | 0.744 | 1.000 | 3161 | tags=39%, list=25%, signal=51% |
| 302 | GOBP\_LEUKOCYTE\_MEDIATED\_IMMUNITY |  | 357 | 0.20 | 1.14 | 0.064 | 0.751 | 1.000 | 2798 | tags=29%, list=22%, signal=35% |
| 303 | GOBP\_CHROMOSOME\_LOCALIZATION |  | 68 | 0.26 | 1.14 | 0.180 | 0.751 | 1.000 | 3729 | tags=35%, list=29%, signal=49% |
| 304 | GOBP\_MITOCHONDRIAL\_MEMBRANE\_ORGANIZATION |  | 90 | 0.25 | 1.14 | 0.193 | 0.751 | 1.000 | 2279 | tags=23%, list=18%, signal=28% |
| 305 | GOBP\_LIPOPROTEIN\_METABOLIC\_PROCESS |  | 103 | 0.24 | 1.14 | 0.181 | 0.749 | 1.000 | 2801 | tags=32%, list=22%, signal=41% |
| 306 | GOBP\_LYSOSOMAL\_TRANSPORT |  | 97 | 0.24 | 1.14 | 0.167 | 0.753 | 1.000 | 3074 | tags=35%, list=24%, signal=46% |
| 307 | GOBP\_WATER\_SOLUBLE\_VITAMIN\_METABOLIC\_PROCESS |  | 52 | 0.27 | 1.14 | 0.229 | 0.754 | 1.000 | 2155 | tags=27%, list=17%, signal=32% |
| 308 | GOBP\_REGULATION\_OF\_MACROPHAGE\_CHEMOTAXIS |  | 25 | 0.33 | 1.14 | 0.284 | 0.756 | 1.000 | 1094 | tags=20%, list=8%, signal=22% |
| 309 | GOBP\_MACROAUTOPHAGY |  | 241 | 0.21 | 1.14 | 0.135 | 0.755 | 1.000 | 1766 | tags=19%, list=14%, signal=22% |
| 310 | GOBP\_PROTEIN\_AUTOUBIQUITINATION |  | 55 | 0.28 | 1.14 | 0.215 | 0.753 | 1.000 | 506 | tags=15%, list=4%, signal=15% |
| 311 | GOBP\_MAINTENANCE\_OF\_SYNAPSE\_STRUCTURE |  | 15 | 0.38 | 1.14 | 0.301 | 0.753 | 1.000 | 891 | tags=13%, list=7%, signal=14% |
| 312 | GOBP\_PYRIMIDINE\_NUCLEOSIDE\_TRIPHOSPHATE\_METABOLIC\_PROCESS |  | 19 | 0.36 | 1.14 | 0.286 | 0.756 | 1.000 | 1428 | tags=26%, list=11%, signal=30% |
| 313 | GOBP\_PROTEIN\_LOCALIZATION\_TO\_MITOCHONDRION |  | 103 | 0.25 | 1.13 | 0.200 | 0.760 | 1.000 | 2390 | tags=26%, list=19%, signal=32% |
| 314 | GOBP\_ESTABLISHMENT\_OF\_PROTEIN\_LOCALIZATION\_TO\_MITOCHONDRIAL\_MEMBRANE |  | 26 | 0.32 | 1.13 | 0.269 | 0.758 | 1.000 | 2244 | tags=27%, list=17%, signal=33% |
| 315 | GOBP\_PH\_REDUCTION |  | 38 | 0.30 | 1.13 | 0.243 | 0.762 | 1.000 | 3678 | tags=42%, list=29%, signal=59% |
| 316 | GOBP\_NUCLEOTIDE\_SUGAR\_METABOLIC\_PROCESS |  | 29 | 0.32 | 1.13 | 0.280 | 0.764 | 1.000 | 1666 | tags=24%, list=13%, signal=28% |
| 317 | GOBP\_REGULATION\_OF\_CARDIAC\_MUSCLE\_CELL\_ACTION\_POTENTIAL |  | 24 | 0.33 | 1.13 | 0.272 | 0.766 | 1.000 | 2030 | tags=25%, list=16%, signal=30% |
| 318 | GOBP\_BUNDLE\_OF\_HIS\_CELL\_TO\_PURKINJE\_MYOCYTE\_COMMUNICATION |  | 15 | 0.38 | 1.13 | 0.302 | 0.767 | 1.000 | 2387 | tags=33%, list=19%, signal=41% |
| 319 | GOBP\_NEGATIVE\_REGULATION\_OF\_ATP\_METABOLIC\_PROCESS |  | 27 | 0.32 | 1.13 | 0.277 | 0.768 | 1.000 | 3410 | tags=41%, list=26%, signal=55% |
| 320 | GOBP\_NUCLEAR\_MEMBRANE\_ORGANIZATION |  | 37 | 0.30 | 1.13 | 0.264 | 0.768 | 1.000 | 2733 | tags=41%, list=21%, signal=51% |
| 321 | GOBP\_PIGMENT\_METABOLIC\_PROCESS |  | 75 | 0.25 | 1.13 | 0.230 | 0.771 | 1.000 | 1575 | tags=20%, list=12%, signal=23% |
| 322 | GOBP\_GLUCOCORTICOID\_METABOLIC\_PROCESS |  | 23 | 0.33 | 1.12 | 0.272 | 0.777 | 1.000 | 539 | tags=17%, list=4%, signal=18% |
| 323 | GOBP\_AMINO\_SUGAR\_METABOLIC\_PROCESS |  | 36 | 0.30 | 1.12 | 0.270 | 0.778 | 1.000 | 1795 | tags=22%, list=14%, signal=26% |
| 324 | GOBP\_COCHLEA\_MORPHOGENESIS |  | 18 | 0.35 | 1.12 | 0.287 | 0.780 | 1.000 | 3130 | tags=44%, list=24%, signal=59% |
| 325 | GOBP\_P\_BODY\_ASSEMBLY |  | 15 | 0.38 | 1.12 | 0.307 | 0.780 | 1.000 | 1687 | tags=33%, list=13%, signal=38% |
| 326 | GOBP\_NEGATIVE\_REGULATION\_OF\_CALCIUM\_ION\_TRANSPORT\_INTO\_CYTOSOL |  | 16 | 0.37 | 1.12 | 0.300 | 0.785 | 1.000 | 3097 | tags=50%, list=24%, signal=66% |
| 327 | GOBP\_SPERM\_FLAGELLUM\_ASSEMBLY |  | 15 | 0.38 | 1.12 | 0.311 | 0.788 | 1.000 | 3042 | tags=47%, list=24%, signal=61% |
| 328 | GOBP\_VIRAL\_RELEASE\_FROM\_HOST\_CELL |  | 25 | 0.32 | 1.12 | 0.293 | 0.791 | 1.000 | 2783 | tags=40%, list=22%, signal=51% |
| 329 | GOBP\_PROTEIN\_TARGETING\_TO\_MEMBRANE |  | 91 | 0.25 | 1.12 | 0.227 | 0.791 | 1.000 | 1694 | tags=23%, list=13%, signal=26% |
| 330 | GOBP\_ORGANOPHOSPHATE\_BIOSYNTHETIC\_PROCESS |  | 447 | 0.20 | 1.11 | 0.114 | 0.793 | 1.000 | 2941 | tags=27%, list=23%, signal=34% |
| 331 | GOBP\_VESICLE\_TARGETING |  | 36 | 0.30 | 1.11 | 0.294 | 0.792 | 1.000 | 2885 | tags=33%, list=22%, signal=43% |
| 332 | GOBP\_NUCLEOSIDE\_PHOSPHATE\_CATABOLIC\_PROCESS |  | 63 | 0.26 | 1.11 | 0.278 | 0.793 | 1.000 | 1068 | tags=19%, list=8%, signal=21% |
| 333 | GOBP\_REGULATION\_OF\_CELLULAR\_AMINE\_METABOLIC\_PROCESS |  | 30 | 0.31 | 1.11 | 0.283 | 0.791 | 1.000 | 2192 | tags=27%, list=17%, signal=32% |
| 334 | GOBP\_MITOTIC\_SPINDLE\_ORGANIZATION |  | 110 | 0.23 | 1.11 | 0.209 | 0.789 | 1.000 | 3778 | tags=34%, list=29%, signal=47% |
| 335 | GOBP\_REGULATION\_OF\_ATP\_METABOLIC\_PROCESS |  | 77 | 0.25 | 1.11 | 0.243 | 0.790 | 1.000 | 2580 | tags=30%, list=20%, signal=37% |
| 336 | GOBP\_POSITIVE\_REGULATION\_OF\_UBIQUITIN\_DEPENDENT\_PROTEIN\_CATABOLIC\_PROCESS |  | 87 | 0.24 | 1.11 | 0.256 | 0.790 | 1.000 | 2399 | tags=28%, list=19%, signal=34% |
| 337 | GOBP\_RETROGRADE\_TRANSPORT\_ENDOSOME\_TO\_GOLGI |  | 67 | 0.26 | 1.11 | 0.244 | 0.793 | 1.000 | 2188 | tags=24%, list=17%, signal=29% |
| 338 | GOBP\_CILIUM\_ORGANIZATION |  | 250 | 0.21 | 1.11 | 0.175 | 0.792 | 1.000 | 2135 | tags=21%, list=17%, signal=24% |
| 339 | GOBP\_CELLULAR\_AMINO\_ACID\_BIOSYNTHETIC\_PROCESS |  | 64 | 0.26 | 1.11 | 0.278 | 0.794 | 1.000 | 2178 | tags=27%, list=17%, signal=32% |
| 340 | GOBP\_GLYCEROL\_ETHER\_METABOLIC\_PROCESS |  | 16 | 0.36 | 1.11 | 0.312 | 0.798 | 1.000 | 1874 | tags=31%, list=15%, signal=37% |
| 341 | GOBP\_ALCOHOL\_METABOLIC\_PROCESS |  | 302 | 0.20 | 1.11 | 0.153 | 0.797 | 1.000 | 2913 | tags=25%, list=23%, signal=32% |
| 342 | GOBP\_COTRANSLATIONAL\_PROTEIN\_TARGETING\_TO\_MEMBRANE |  | 18 | 0.35 | 1.11 | 0.325 | 0.796 | 1.000 | 1272 | tags=17%, list=10%, signal=18% |
| 343 | GOBP\_CARBOHYDRATE\_METABOLIC\_PROCESS |  | 488 | 0.19 | 1.11 | 0.110 | 0.794 | 1.000 | 2656 | tags=25%, list=21%, signal=30% |
| 344 | GOBP\_STEROL\_BIOSYNTHETIC\_PROCESS |  | 57 | 0.26 | 1.10 | 0.298 | 0.799 | 1.000 | 3146 | tags=35%, list=24%, signal=46% |
| 345 | GOBP\_VITAMIN\_METABOLIC\_PROCESS |  | 92 | 0.24 | 1.10 | 0.246 | 0.802 | 1.000 | 2372 | tags=27%, list=18%, signal=33% |
| 346 | GOBP\_POSITIVE\_REGULATION\_OF\_INTERFERON\_GAMMA\_PRODUCTION |  | 68 | 0.26 | 1.10 | 0.251 | 0.801 | 1.000 | 3579 | tags=41%, list=28%, signal=57% |
| 347 | GOBP\_SPINAL\_CORD\_MOTOR\_NEURON\_DIFFERENTIATION |  | 22 | 0.33 | 1.10 | 0.311 | 0.801 | 1.000 | 3847 | tags=36%, list=30%, signal=52% |
| 348 | GOBP\_MEMBRANE\_LIPID\_BIOSYNTHETIC\_PROCESS |  | 108 | 0.24 | 1.10 | 0.261 | 0.804 | 1.000 | 2979 | tags=32%, list=23%, signal=42% |
| 349 | GOBP\_RESPONSE\_TO\_AMINE |  | 39 | 0.28 | 1.10 | 0.295 | 0.804 | 1.000 | 3913 | tags=46%, list=30%, signal=66% |
| 350 | GOBP\_NUCLEOTIDE\_PHOSPHORYLATION |  | 88 | 0.24 | 1.10 | 0.249 | 0.811 | 1.000 | 876 | tags=15%, list=7%, signal=16% |
| 351 | GOBP\_MICROGLIAL\_CELL\_ACTIVATION |  | 41 | 0.28 | 1.09 | 0.294 | 0.823 | 1.000 | 2098 | tags=27%, list=16%, signal=32% |
| 352 | GOBP\_TRNA\_METHYLATION |  | 28 | 0.31 | 1.09 | 0.320 | 0.820 | 1.000 | 2181 | tags=25%, list=17%, signal=30% |
| 353 | GOBP\_FATTY\_ACID\_BETA\_OXIDATION |  | 67 | 0.25 | 1.09 | 0.279 | 0.819 | 1.000 | 1925 | tags=24%, list=15%, signal=28% |
| 354 | GOBP\_POSITIVE\_REGULATION\_OF\_ISOTYPE\_SWITCHING |  | 22 | 0.33 | 1.09 | 0.331 | 0.817 | 1.000 | 787 | tags=14%, list=6%, signal=14% |
| 355 | GOBP\_ENDOPLASMIC\_RETICULUM\_CALCIUM\_ION\_HOMEOSTASIS |  | 22 | 0.33 | 1.09 | 0.312 | 0.817 | 1.000 | 2781 | tags=27%, list=22%, signal=35% |
| 356 | GOBP\_CHONDROCYTE\_DEVELOPMENT |  | 25 | 0.32 | 1.09 | 0.324 | 0.817 | 1.000 | 2128 | tags=28%, list=16%, signal=33% |
| 357 | GOBP\_DNA\_SYNTHESIS\_INVOLVED\_IN\_DNA\_REPAIR |  | 29 | 0.31 | 1.09 | 0.312 | 0.819 | 1.000 | 2300 | tags=31%, list=18%, signal=38% |
| 358 | GOBP\_CELL\_JUNCTION\_DISASSEMBLY |  | 20 | 0.34 | 1.09 | 0.353 | 0.819 | 1.000 | 3400 | tags=45%, list=26%, signal=61% |
| 359 | GOBP\_REGULATION\_OF\_OXIDOREDUCTASE\_ACTIVITY |  | 83 | 0.25 | 1.09 | 0.280 | 0.817 | 1.000 | 2776 | tags=29%, list=22%, signal=37% |
| 360 | GOBP\_REGULATION\_OF\_LIPOPROTEIN\_PARTICLE\_CLEARANCE |  | 19 | 0.34 | 1.09 | 0.330 | 0.821 | 1.000 | 1855 | tags=37%, list=14%, signal=43% |
| 361 | GOBP\_NEGATIVE\_REGULATION\_OF\_FATTY\_ACID\_METABOLIC\_PROCESS |  | 25 | 0.32 | 1.09 | 0.330 | 0.822 | 1.000 | 2549 | tags=32%, list=20%, signal=40% |
| 362 | GOBP\_NEGATIVE\_REGULATION\_OF\_INTRINSIC\_APOPTOTIC\_SIGNALING\_PATHWAY\_BY\_P53\_CLASS\_MEDIATOR |  | 19 | 0.34 | 1.08 | 0.334 | 0.837 | 1.000 | 2163 | tags=26%, list=17%, signal=32% |
| 363 | GOBP\_ANTIVIRAL\_INNATE\_IMMUNE\_RESPONSE |  | 15 | 0.36 | 1.08 | 0.374 | 0.836 | 1.000 | 1754 | tags=40%, list=14%, signal=46% |
| 364 | GOBP\_SPINDLE\_ASSEMBLY |  | 104 | 0.23 | 1.08 | 0.282 | 0.841 | 1.000 | 1542 | tags=16%, list=12%, signal=18% |
| 365 | GOBP\_IMMUNE\_RESPONSE\_REGULATING\_CELL\_SURFACE\_RECEPTOR\_SIGNALING\_PATHWAY |  | 235 | 0.20 | 1.08 | 0.244 | 0.842 | 1.000 | 3243 | tags=32%, list=25%, signal=42% |
| 366 | GOBP\_CELLULAR\_MODIFIED\_AMINO\_ACID\_METABOLIC\_PROCESS |  | 153 | 0.22 | 1.08 | 0.263 | 0.844 | 1.000 | 2621 | tags=24%, list=20%, signal=30% |
| 367 | GOBP\_FATTY\_ACID\_METABOLIC\_PROCESS |  | 303 | 0.20 | 1.08 | 0.230 | 0.848 | 1.000 | 2017 | tags=20%, list=16%, signal=23% |
| 368 | GOBP\_STEROL\_METABOLIC\_PROCESS |  | 132 | 0.22 | 1.08 | 0.286 | 0.849 | 1.000 | 1888 | tags=18%, list=15%, signal=21% |
| 369 | GOBP\_REGULATION\_OF\_AEROBIC\_RESPIRATION |  | 30 | 0.30 | 1.08 | 0.336 | 0.849 | 1.000 | 2517 | tags=33%, list=20%, signal=41% |
| 370 | GOBP\_ACUTE\_INFLAMMATORY\_RESPONSE\_TO\_ANTIGENIC\_STIMULUS |  | 21 | 0.33 | 1.08 | 0.329 | 0.852 | 1.000 | 2764 | tags=33%, list=21%, signal=42% |
| 371 | GOBP\_POSITIVE\_REGULATION\_OF\_ENDOCYTOSIS |  | 84 | 0.24 | 1.08 | 0.286 | 0.853 | 1.000 | 2560 | tags=36%, list=20%, signal=44% |
| 372 | GOBP\_CARBOHYDRATE\_PHOSPHORYLATION |  | 23 | 0.32 | 1.07 | 0.348 | 0.856 | 1.000 | 256 | tags=13%, list=2%, signal=13% |
| 373 | GOBP\_ZINC\_ION\_HOMEOSTASIS |  | 27 | 0.31 | 1.07 | 0.343 | 0.860 | 1.000 | 2513 | tags=33%, list=19%, signal=41% |
| 374 | GOBP\_SPHINGOMYELIN\_METABOLIC\_PROCESS |  | 15 | 0.37 | 1.07 | 0.374 | 0.861 | 1.000 | 2913 | tags=40%, list=23%, signal=52% |
| 375 | GOBP\_RESPONSE\_TO\_ISCHEMIA |  | 45 | 0.27 | 1.07 | 0.322 | 0.865 | 1.000 | 2186 | tags=24%, list=17%, signal=29% |
| 376 | GOBP\_MAINTENANCE\_OF\_LOCATION\_IN\_CELL |  | 183 | 0.21 | 1.07 | 0.297 | 0.866 | 1.000 | 2293 | tags=21%, list=18%, signal=26% |
| 377 | GOBP\_PEPTIDE\_ANTIGEN\_ASSEMBLY\_WITH\_MHC\_PROTEIN\_COMPLEX |  | 17 | 0.34 | 1.07 | 0.340 | 0.874 | 1.000 | 2147 | tags=35%, list=17%, signal=42% |
| 378 | GOBP\_MEMBRANE\_FUSION |  | 126 | 0.22 | 1.07 | 0.317 | 0.876 | 1.000 | 908 | tags=13%, list=7%, signal=14% |
| 379 | GOBP\_ISOPRENOID\_METABOLIC\_PROCESS |  | 90 | 0.23 | 1.07 | 0.346 | 0.878 | 1.000 | 3301 | tags=31%, list=26%, signal=42% |
| 380 | GOBP\_MONOCARBOXYLIC\_ACID\_CATABOLIC\_PROCESS |  | 101 | 0.22 | 1.06 | 0.330 | 0.880 | 1.000 | 1925 | tags=21%, list=15%, signal=24% |
| 381 | GOBP\_REGULATION\_OF\_OXIDATIVE\_STRESS\_INDUCED\_INTRINSIC\_APOPTOTIC\_SIGNALING\_PATHWAY |  | 27 | 0.31 | 1.06 | 0.336 | 0.878 | 1.000 | 2413 | tags=44%, list=19%, signal=55% |
| 382 | GOBP\_GRANULOCYTE\_ACTIVATION |  | 42 | 0.27 | 1.06 | 0.340 | 0.877 | 1.000 | 2962 | tags=38%, list=23%, signal=49% |
| 383 | GOBP\_POLYSACCHARIDE\_CATABOLIC\_PROCESS |  | 18 | 0.34 | 1.06 | 0.366 | 0.881 | 1.000 | 3978 | tags=56%, list=31%, signal=80% |
| 384 | GOBP\_REGULATION\_OF\_ATP\_DEPENDENT\_ACTIVITY |  | 63 | 0.25 | 1.06 | 0.341 | 0.879 | 1.000 | 2781 | tags=32%, list=22%, signal=40% |
| 385 | GOBP\_POSITIVE\_REGULATION\_OF\_REACTIVE\_OXYGEN\_SPECIES\_METABOLIC\_PROCESS |  | 60 | 0.25 | 1.06 | 0.327 | 0.882 | 1.000 | 2490 | tags=30%, list=19%, signal=37% |
| 386 | GOBP\_CELLULAR\_RESPONSE\_TO\_GAMMA\_RADIATION |  | 28 | 0.30 | 1.06 | 0.347 | 0.882 | 1.000 | 1581 | tags=25%, list=12%, signal=28% |
| 387 | GOBP\_CD8\_POSITIVE\_ALPHA\_BETA\_T\_CELL\_ACTIVATION |  | 21 | 0.32 | 1.06 | 0.367 | 0.883 | 1.000 | 1962 | tags=33%, list=15%, signal=39% |
| 388 | GOBP\_REGULATION\_OF\_GENERATION\_OF\_PRECURSOR\_METABOLITES\_AND\_ENERGY |  | 114 | 0.22 | 1.06 | 0.330 | 0.885 | 1.000 | 2580 | tags=28%, list=20%, signal=35% |
| 389 | GOBP\_INTERLEUKIN\_10\_PRODUCTION |  | 50 | 0.26 | 1.06 | 0.324 | 0.884 | 1.000 | 2821 | tags=32%, list=22%, signal=41% |
| 390 | GOBP\_POSITIVE\_REGULATION\_OF\_T\_CELL\_PROLIFERATION |  | 87 | 0.24 | 1.06 | 0.335 | 0.889 | 1.000 | 2599 | tags=26%, list=20%, signal=33% |
| 391 | GOBP\_NUCLEOSIDE\_DIPHOSPHATE\_METABOLIC\_PROCESS |  | 109 | 0.22 | 1.06 | 0.307 | 0.890 | 1.000 | 2580 | tags=26%, list=20%, signal=32% |
| 392 | GOBP\_POSITIVE\_REGULATION\_OF\_RELEASE\_OF\_CYTOCHROME\_C\_FROM\_MITOCHONDRIA |  | 25 | 0.31 | 1.05 | 0.384 | 0.892 | 1.000 | 2097 | tags=28%, list=16%, signal=33% |
| 393 | GOBP\_REGULATION\_OF\_RUFFLE\_ASSEMBLY |  | 26 | 0.30 | 1.05 | 0.373 | 0.890 | 1.000 | 2437 | tags=31%, list=19%, signal=38% |
| 394 | GOBP\_DIOL\_METABOLIC\_PROCESS |  | 21 | 0.32 | 1.05 | 0.372 | 0.896 | 1.000 | 3405 | tags=48%, list=26%, signal=65% |
| 395 | GOBP\_TERPENOID\_METABOLIC\_PROCESS |  | 73 | 0.24 | 1.05 | 0.358 | 0.909 | 1.000 | 3301 | tags=33%, list=26%, signal=44% |
| 396 | GOBP\_AUTOPHAGY\_OF\_MITOCHONDRION |  | 77 | 0.23 | 1.05 | 0.365 | 0.907 | 1.000 | 2580 | tags=23%, list=20%, signal=29% |
| 397 | GOBP\_RESPONSE\_TO\_INTERFERON\_BETA |  | 26 | 0.30 | 1.05 | 0.387 | 0.916 | 1.000 | 616 | tags=15%, list=5%, signal=16% |
| 398 | GOBP\_ADULT\_LOCOMOTORY\_BEHAVIOR |  | 66 | 0.24 | 1.05 | 0.358 | 0.914 | 1.000 | 1972 | tags=26%, list=15%, signal=30% |
| 399 | GOBP\_EXTRACELLULAR\_TRANSPORT |  | 24 | 0.31 | 1.05 | 0.388 | 0.913 | 1.000 | 790 | tags=17%, list=6%, signal=18% |
| 400 | GOBP\_GLUTAMATE\_METABOLIC\_PROCESS |  | 27 | 0.30 | 1.05 | 0.379 | 0.912 | 1.000 | 2534 | tags=41%, list=20%, signal=51% |
| 401 | GOBP\_TOLL\_LIKE\_RECEPTOR\_4\_SIGNALING\_PATHWAY |  | 32 | 0.28 | 1.04 | 0.387 | 0.913 | 1.000 | 2542 | tags=31%, list=20%, signal=39% |
| 402 | GOBP\_RECEPTOR\_CLUSTERING |  | 47 | 0.26 | 1.04 | 0.348 | 0.912 | 1.000 | 2991 | tags=30%, list=23%, signal=39% |
| 403 | GOBP\_HISTONE\_H4\_K16\_ACETYLATION |  | 24 | 0.31 | 1.04 | 0.379 | 0.913 | 1.000 | 2825 | tags=42%, list=22%, signal=53% |
| 404 | GOBP\_DNA\_UNWINDING\_INVOLVED\_IN\_DNA\_REPLICATION |  | 22 | 0.31 | 1.04 | 0.377 | 0.913 | 1.000 | 1581 | tags=27%, list=12%, signal=31% |
| 405 | GOBP\_TRANSLESION\_SYNTHESIS |  | 17 | 0.33 | 1.04 | 0.372 | 0.912 | 1.000 | 2889 | tags=35%, list=22%, signal=45% |
| 406 | GOBP\_ACTIVATED\_T\_CELL\_PROLIFERATION |  | 41 | 0.27 | 1.04 | 0.404 | 0.926 | 1.000 | 2522 | tags=29%, list=20%, signal=36% |
| 407 | GOBP\_ORGANELLE\_DISASSEMBLY |  | 103 | 0.22 | 1.04 | 0.354 | 0.924 | 1.000 | 2580 | tags=23%, list=20%, signal=29% |
| 408 | GOBP\_PURINE\_NUCLEOBASE\_METABOLIC\_PROCESS |  | 17 | 0.34 | 1.04 | 0.398 | 0.925 | 1.000 | 1518 | tags=24%, list=12%, signal=27% |
| 409 | GOBP\_RENAL\_FILTRATION |  | 22 | 0.31 | 1.04 | 0.386 | 0.925 | 1.000 | 2088 | tags=36%, list=16%, signal=43% |
| 410 | GOBP\_REGULATION\_OF\_MICROTUBULE\_POLYMERIZATION\_OR\_DEPOLYMERIZATION |  | 75 | 0.23 | 1.04 | 0.369 | 0.929 | 1.000 | 1542 | tags=20%, list=12%, signal=23% |
| 411 | GOBP\_POSITIVE\_REGULATION\_OF\_MONOCYTE\_CHEMOTAXIS |  | 17 | 0.33 | 1.04 | 0.410 | 0.929 | 1.000 | 1815 | tags=29%, list=14%, signal=34% |
| 412 | GOBP\_VENTRICULAR\_CARDIAC\_MUSCLE\_CELL\_ACTION\_POTENTIAL |  | 29 | 0.29 | 1.03 | 0.407 | 0.928 | 1.000 | 2078 | tags=17%, list=16%, signal=21% |
| 413 | GOBP\_CARBOHYDRATE\_CATABOLIC\_PROCESS |  | 134 | 0.21 | 1.03 | 0.363 | 0.939 | 1.000 | 2580 | tags=27%, list=20%, signal=33% |
| 414 | GOBP\_ENDOSOME\_TO\_LYSOSOME\_TRANSPORT |  | 46 | 0.26 | 1.03 | 0.395 | 0.939 | 1.000 | 3518 | tags=41%, list=27%, signal=57% |
| 415 | GOBP\_DNA\_BIOSYNTHETIC\_PROCESS |  | 165 | 0.20 | 1.03 | 0.349 | 0.943 | 1.000 | 2111 | tags=23%, list=16%, signal=27% |
| 416 | GOBP\_MICROTUBULE\_DEPOLYMERIZATION |  | 39 | 0.27 | 1.03 | 0.401 | 0.953 | 1.000 | 1502 | tags=21%, list=12%, signal=23% |
| 417 | GOBP\_REGULATION\_OF\_CALCIUM\_MEDIATED\_SIGNALING |  | 64 | 0.24 | 1.02 | 0.394 | 0.958 | 1.000 | 2937 | tags=31%, list=23%, signal=40% |
| 418 | GOBP\_POSITIVE\_REGULATION\_OF\_B\_CELL\_MEDIATED\_IMMUNITY |  | 34 | 0.28 | 1.02 | 0.387 | 0.961 | 1.000 | 889 | tags=15%, list=7%, signal=16% |
| 419 | GOBP\_POSITIVE\_REGULATION\_OF\_MACROPHAGE\_CHEMOTAXIS |  | 16 | 0.33 | 1.02 | 0.396 | 0.961 | 1.000 | 757 | tags=19%, list=6%, signal=20% |
| 420 | GOBP\_PROTEIN\_DEMANNOSYLATION |  | 15 | 0.34 | 1.02 | 0.417 | 0.962 | 1.000 | 3447 | tags=40%, list=27%, signal=55% |
| 421 | GOBP\_ORGANELLE\_MEMBRANE\_FUSION |  | 89 | 0.22 | 1.02 | 0.414 | 0.962 | 1.000 | 824 | tags=12%, list=6%, signal=13% |
| 422 | GOBP\_RESPONSE\_TO\_DIETARY\_EXCESS |  | 22 | 0.30 | 1.02 | 0.409 | 0.963 | 1.000 | 2776 | tags=32%, list=22%, signal=40% |
| 423 | GOBP\_ADAPTIVE\_IMMUNE\_RESPONSE\_BASED\_ON\_SOMATIC\_RECOMBINATION\_OF\_IMMUNE\_RECEPTORS\_BUILT\_FROM\_IMMUNOGLOBULIN\_SUPERFAMILY\_DOMAINS |  | 275 | 0.19 | 1.02 | 0.374 | 0.962 | 1.000 | 2798 | tags=28%, list=22%, signal=35% |
| 424 | GOBP\_PTERIDINE\_CONTAINING\_COMPOUND\_METABOLIC\_PROCESS |  | 29 | 0.28 | 1.02 | 0.413 | 0.960 | 1.000 | 401 | tags=10%, list=3%, signal=11% |
| 425 | GOBP\_POSTREPLICATION\_REPAIR |  | 26 | 0.29 | 1.02 | 0.420 | 0.960 | 1.000 | 3028 | tags=31%, list=23%, signal=40% |
| 426 | GOBP\_MEIOTIC\_CHROMOSOME\_SEPARATION |  | 16 | 0.34 | 1.02 | 0.431 | 0.961 | 1.000 | 3458 | tags=44%, list=27%, signal=60% |
| 427 | GOBP\_VERY\_LONG\_CHAIN\_FATTY\_ACID\_METABOLIC\_PROCESS |  | 28 | 0.29 | 1.02 | 0.424 | 0.959 | 1.000 | 3525 | tags=39%, list=27%, signal=54% |
| 428 | GOBP\_NEGATIVE\_REGULATION\_OF\_SIGNAL\_TRANSDUCTION\_BY\_P53\_CLASS\_MEDIATOR |  | 29 | 0.29 | 1.02 | 0.448 | 0.958 | 1.000 | 2163 | tags=28%, list=17%, signal=33% |
| 429 | GOBP\_ADULT\_BEHAVIOR |  | 117 | 0.21 | 1.02 | 0.401 | 0.959 | 1.000 | 1972 | tags=21%, list=15%, signal=25% |
| 430 | GOBP\_REGULATION\_OF\_MICROTUBULE\_DEPOLYMERIZATION |  | 29 | 0.28 | 1.02 | 0.420 | 0.965 | 1.000 | 1502 | tags=24%, list=12%, signal=27% |
| 431 | GOBP\_ANTIGEN\_PROCESSING\_AND\_PRESENTATION\_OF\_PEPTIDE\_ANTIGEN\_VIA\_MHC\_CLASS\_I |  | 27 | 0.28 | 1.02 | 0.401 | 0.966 | 1.000 | 1532 | tags=22%, list=12%, signal=25% |
| 432 | GOBP\_AMINO\_ACID\_ACTIVATION |  | 36 | 0.27 | 1.02 | 0.397 | 0.965 | 1.000 | 1913 | tags=25%, list=15%, signal=29% |
| 433 | GOBP\_AMP\_METABOLIC\_PROCESS |  | 21 | 0.30 | 1.02 | 0.431 | 0.964 | 1.000 | 1789 | tags=24%, list=14%, signal=28% |
| 434 | GOBP\_POSITIVE\_REGULATION\_OF\_TORC1\_SIGNALING |  | 17 | 0.33 | 1.01 | 0.442 | 0.963 | 1.000 | 2065 | tags=35%, list=16%, signal=42% |
| 435 | GOBP\_SMALL\_MOLECULE\_BIOSYNTHETIC\_PROCESS |  | 467 | 0.18 | 1.01 | 0.400 | 0.962 | 1.000 | 2681 | tags=24%, list=21%, signal=29% |
| 436 | GOBP\_REGULATION\_OF\_TOR\_SIGNALING |  | 72 | 0.23 | 1.01 | 0.406 | 0.960 | 1.000 | 1325 | tags=18%, list=10%, signal=20% |
| 437 | GOBP\_MEMBRANE\_RAFT\_ORGANIZATION |  | 22 | 0.30 | 1.01 | 0.445 | 0.960 | 1.000 | 2616 | tags=27%, list=20%, signal=34% |
| 438 | GOBP\_NEGATIVE\_REGULATION\_OF\_FATTY\_ACID\_BIOSYNTHETIC\_PROCESS |  | 15 | 0.35 | 1.01 | 0.429 | 0.958 | 1.000 | 2317 | tags=27%, list=18%, signal=32% |
| 439 | GOBP\_NEGATIVE\_REGULATION\_OF\_AUTOPHAGY |  | 70 | 0.23 | 1.01 | 0.436 | 0.956 | 1.000 | 3034 | tags=34%, list=24%, signal=45% |
| 440 | GOBP\_NADP\_METABOLIC\_PROCESS |  | 32 | 0.28 | 1.01 | 0.426 | 0.959 | 1.000 | 3759 | tags=47%, list=29%, signal=66% |
| 441 | GOBP\_PHOSPHATIDYLINOSITOL\_BIOSYNTHETIC\_PROCESS |  | 108 | 0.21 | 1.01 | 0.412 | 0.960 | 1.000 | 1675 | tags=18%, list=13%, signal=20% |
| 442 | GOBP\_ACTIVATION\_OF\_IMMUNE\_RESPONSE |  | 284 | 0.19 | 1.01 | 0.415 | 0.961 | 1.000 | 3243 | tags=31%, list=25%, signal=40% |
| 443 | GOBP\_GLYCOSAMINOGLYCAN\_CATABOLIC\_PROCESS |  | 21 | 0.31 | 1.01 | 0.444 | 0.963 | 1.000 | 1506 | tags=24%, list=12%, signal=27% |
| 444 | GOBP\_ORGANIC\_HYDROXY\_COMPOUND\_METABOLIC\_PROCESS |  | 466 | 0.18 | 1.01 | 0.407 | 0.964 | 1.000 | 2913 | tags=24%, list=23%, signal=30% |
| 445 | GOBP\_RESPIRATORY\_GASEOUS\_EXCHANGE\_BY\_RESPIRATORY\_SYSTEM |  | 58 | 0.24 | 1.01 | 0.447 | 0.966 | 1.000 | 2398 | tags=29%, list=19%, signal=36% |
| 446 | GOBP\_REGULATION\_OF\_PROTEASOMAL\_UBIQUITIN\_DEPENDENT\_PROTEIN\_CATABOLIC\_PROCESS |  | 109 | 0.21 | 1.01 | 0.440 | 0.969 | 1.000 | 2332 | tags=23%, list=18%, signal=28% |
| 447 | GOBP\_OLIGOSACCHARIDE\_METABOLIC\_PROCESS |  | 51 | 0.24 | 1.01 | 0.436 | 0.969 | 1.000 | 2979 | tags=33%, list=23%, signal=43% |
| 448 | GOBP\_REGULATION\_OF\_AUTOPHAGY\_OF\_MITOCHONDRION |  | 34 | 0.27 | 1.00 | 0.438 | 0.979 | 1.000 | 2580 | tags=29%, list=20%, signal=37% |
| 449 | GOBP\_CELLULAR\_RESPONSE\_TO\_OXYGEN\_RADICAL |  | 19 | 0.31 | 1.00 | 0.445 | 0.983 | 1.000 | 3113 | tags=37%, list=24%, signal=48% |
| 450 | GOBP\_INTERSTRAND\_CROSS\_LINK\_REPAIR |  | 24 | 0.30 | 1.00 | 0.431 | 0.987 | 1.000 | 2001 | tags=38%, list=16%, signal=44% |
| 451 | GOBP\_PLASMA\_LIPOPROTEIN\_PARTICLE\_CLEARANCE |  | 36 | 0.27 | 1.00 | 0.470 | 0.988 | 1.000 | 1855 | tags=28%, list=14%, signal=32% |
| 452 | GOBP\_AMINOGLYCAN\_CATABOLIC\_PROCESS |  | 27 | 0.29 | 1.00 | 0.418 | 0.988 | 1.000 | 1506 | tags=22%, list=12%, signal=25% |
| 453 | GOBP\_POSITIVE\_REGULATION\_OF\_INFLAMMATORY\_RESPONSE |  | 119 | 0.21 | 1.00 | 0.428 | 0.986 | 1.000 | 2769 | tags=27%, list=21%, signal=34% |
| 454 | GOBP\_NEGATIVE\_REGULATION\_OF\_PROTEIN\_KINASE\_B\_SIGNALING |  | 39 | 0.26 | 1.00 | 0.443 | 0.989 | 1.000 | 2522 | tags=26%, list=20%, signal=32% |
| 455 | GOBP\_NEGATIVE\_REGULATION\_OF\_ORGANELLE\_ORGANIZATION |  | 285 | 0.18 | 1.00 | 0.456 | 0.988 | 1.000 | 2580 | tags=22%, list=20%, signal=27% |
| 456 | GOBP\_NUCLEOSIDE\_CATABOLIC\_PROCESS |  | 21 | 0.30 | 1.00 | 0.476 | 0.990 | 1.000 | 1955 | tags=33%, list=15%, signal=39% |
| 457 | GOBP\_PROTEOGLYCAN\_BIOSYNTHETIC\_PROCESS |  | 44 | 0.25 | 1.00 | 0.486 | 0.991 | 1.000 | 3497 | tags=41%, list=27%, signal=56% |
| 458 | GOBP\_POSITIVE\_REGULATION\_OF\_CALCIUM\_ION\_TRANSMEMBRANE\_TRANSPORT |  | 62 | 0.23 | 1.00 | 0.442 | 0.989 | 1.000 | 1886 | tags=23%, list=15%, signal=26% |
| 459 | GOBP\_PYRUVATE\_METABOLIC\_PROCESS |  | 99 | 0.21 | 1.00 | 0.466 | 0.987 | 1.000 | 2629 | tags=26%, list=20%, signal=33% |
| 460 | GOBP\_PHAGOCYTOSIS |  | 225 | 0.19 | 1.00 | 0.466 | 0.986 | 1.000 | 2803 | tags=27%, list=22%, signal=33% |
| 461 | GOBP\_SULFUR\_COMPOUND\_CATABOLIC\_PROCESS |  | 23 | 0.30 | 1.00 | 0.465 | 0.985 | 1.000 | 1017 | tags=17%, list=8%, signal=19% |
| 462 | GOBP\_MITOTIC\_RECOMBINATION |  | 22 | 0.30 | 1.00 | 0.455 | 0.985 | 1.000 | 2182 | tags=32%, list=17%, signal=38% |
| 463 | GOBP\_CELLULAR\_PIGMENTATION |  | 41 | 0.26 | 1.00 | 0.421 | 0.983 | 1.000 | 3569 | tags=41%, list=28%, signal=57% |
| 464 | GOBP\_NEGATIVE\_REGULATION\_OF\_HUMORAL\_IMMUNE\_RESPONSE |  | 15 | 0.34 | 0.99 | 0.466 | 0.982 | 1.000 | 3435 | tags=53%, list=27%, signal=73% |
| 465 | GOBP\_MYELOID\_CELL\_ACTIVATION\_INVOLVED\_IN\_IMMUNE\_RESPONSE |  | 87 | 0.22 | 0.99 | 0.471 | 0.982 | 1.000 | 2478 | tags=24%, list=19%, signal=30% |
| 466 | GOBP\_CATECHOL\_CONTAINING\_COMPOUND\_METABOLIC\_PROCESS |  | 51 | 0.24 | 0.99 | 0.472 | 0.983 | 1.000 | 2531 | tags=27%, list=20%, signal=34% |
| 467 | GOBP\_ORGANOPHOSPHATE\_CATABOLIC\_PROCESS |  | 117 | 0.21 | 0.99 | 0.462 | 0.982 | 1.000 | 1374 | tags=17%, list=11%, signal=19% |
| 468 | GOBP\_VESICLE\_MEDIATED\_TRANSPORT\_BETWEEN\_ENDOSOMAL\_COMPARTMENTS |  | 37 | 0.27 | 0.99 | 0.429 | 0.980 | 1.000 | 3037 | tags=30%, list=24%, signal=39% |
| 469 | GOBP\_PROTEIN\_KINASE\_A\_SIGNALING |  | 30 | 0.28 | 0.99 | 0.458 | 0.978 | 1.000 | 2492 | tags=30%, list=19%, signal=37% |
| 470 | GOBP\_SMOOTHENED\_SIGNALING\_PATHWAY |  | 103 | 0.21 | 0.99 | 0.442 | 0.977 | 1.000 | 1882 | tags=19%, list=15%, signal=23% |
| 471 | GOBP\_N\_ACETYLGLUCOSAMINE\_METABOLIC\_PROCESS |  | 15 | 0.33 | 0.99 | 0.466 | 0.976 | 1.000 | 1795 | tags=27%, list=14%, signal=31% |
| 472 | GOBP\_PROTEIN\_PALMITOYLATION |  | 16 | 0.33 | 0.99 | 0.462 | 0.975 | 1.000 | 1694 | tags=38%, list=13%, signal=43% |
| 473 | GOBP\_MITOTIC\_SISTER\_CHROMATID\_SEGREGATION |  | 139 | 0.20 | 0.99 | 0.475 | 0.974 | 1.000 | 3773 | tags=34%, list=29%, signal=47% |
| 474 | GOBP\_POSITIVE\_REGULATION\_OF\_T\_HELPER\_CELL\_DIFFERENTIATION |  | 18 | 0.32 | 0.99 | 0.457 | 0.973 | 1.000 | 2760 | tags=33%, list=21%, signal=42% |
| 475 | GOBP\_DOUBLE\_STRAND\_BREAK\_REPAIR\_VIA\_NONHOMOLOGOUS\_END\_JOINING |  | 58 | 0.24 | 0.99 | 0.467 | 0.974 | 1.000 | 1696 | tags=21%, list=13%, signal=24% |
| 476 | GOBP\_NEGATIVE\_REGULATION\_OF\_INTERLEUKIN\_1\_BETA\_PRODUCTION |  | 21 | 0.30 | 0.99 | 0.473 | 0.975 | 1.000 | 2286 | tags=33%, list=18%, signal=40% |
| 477 | GOBP\_PURINE\_CONTAINING\_COMPOUND\_CATABOLIC\_PROCESS |  | 38 | 0.26 | 0.99 | 0.465 | 0.977 | 1.000 | 1955 | tags=29%, list=15%, signal=34% |
| 478 | GOBP\_RESPONSE\_TO\_ZINC\_ION |  | 41 | 0.25 | 0.99 | 0.478 | 0.979 | 1.000 | 3661 | tags=46%, list=28%, signal=64% |
| 479 | GOBP\_POSITIVE\_REGULATION\_OF\_OSTEOCLAST\_DIFFERENTIATION |  | 19 | 0.31 | 0.99 | 0.447 | 0.977 | 1.000 | 2557 | tags=26%, list=20%, signal=33% |
| 480 | GOBP\_ZINC\_ION\_TRANSPORT |  | 16 | 0.33 | 0.99 | 0.468 | 0.986 | 1.000 | 2513 | tags=38%, list=19%, signal=47% |
| 481 | GOBP\_RESPONSE\_TO\_AMPHETAMINE |  | 28 | 0.28 | 0.99 | 0.472 | 0.986 | 1.000 | 1054 | tags=18%, list=8%, signal=19% |
| 482 | GOBP\_SPINAL\_CORD\_DEVELOPMENT |  | 73 | 0.23 | 0.99 | 0.487 | 0.984 | 1.000 | 1973 | tags=19%, list=15%, signal=23% |
| 483 | GOBP\_POSITIVE\_REGULATION\_OF\_PROTEIN\_TARGETING\_TO\_MEMBRANE |  | 25 | 0.28 | 0.98 | 0.466 | 0.986 | 1.000 | 963 | tags=24%, list=7%, signal=26% |
| 484 | GOBP\_TUMOR\_NECROSIS\_FACTOR\_SUPERFAMILY\_CYTOKINE\_PRODUCTION |  | 150 | 0.20 | 0.98 | 0.509 | 0.985 | 1.000 | 2478 | tags=26%, list=19%, signal=32% |
| 485 | GOBP\_PROTEIN\_EXIT\_FROM\_ENDOPLASMIC\_RETICULUM |  | 36 | 0.26 | 0.98 | 0.478 | 0.989 | 1.000 | 2780 | tags=25%, list=22%, signal=32% |
| 486 | GOBP\_REGULATION\_OF\_IMMUNOGLOBULIN\_PRODUCTION |  | 61 | 0.23 | 0.98 | 0.502 | 0.988 | 1.000 | 2540 | tags=25%, list=20%, signal=30% |
| 487 | GOBP\_SARCOPLASMIC\_RETICULUM\_CALCIUM\_ION\_TRANSPORT |  | 32 | 0.27 | 0.98 | 0.491 | 0.991 | 1.000 | 3141 | tags=41%, list=24%, signal=54% |
| 488 | GOBP\_REGULATION\_OF\_PROTON\_TRANSPORT |  | 20 | 0.30 | 0.98 | 0.486 | 0.991 | 1.000 | 1940 | tags=30%, list=15%, signal=35% |
| 489 | GOBP\_PROTEIN\_LOCALIZATION\_TO\_CILIUM |  | 50 | 0.24 | 0.98 | 0.462 | 0.989 | 1.000 | 1934 | tags=22%, list=15%, signal=26% |
| 490 | GOBP\_NEGATIVE\_REGULATION\_OF\_MICROTUBULE\_POLYMERIZATION\_OR\_DEPOLYMERIZATION |  | 38 | 0.26 | 0.98 | 0.469 | 0.988 | 1.000 | 1389 | tags=21%, list=11%, signal=24% |
| 491 | GOBP\_PYRIDINE\_CONTAINING\_COMPOUND\_BIOSYNTHETIC\_PROCESS |  | 19 | 0.31 | 0.98 | 0.475 | 0.990 | 1.000 | 3209 | tags=47%, list=25%, signal=63% |
| 492 | GOBP\_NUCLEOSIDE\_MONOPHOSPHATE\_BIOSYNTHETIC\_PROCESS |  | 38 | 0.26 | 0.98 | 0.444 | 0.988 | 1.000 | 1178 | tags=16%, list=9%, signal=17% |
| 493 | GOBP\_REGULATION\_OF\_PROTEASOMAL\_PROTEIN\_CATABOLIC\_PROCESS |  | 150 | 0.20 | 0.98 | 0.503 | 0.987 | 1.000 | 2332 | tags=21%, list=18%, signal=26% |
| 494 | GOBP\_CYTOPLASMIC\_SEQUESTERING\_OF\_PROTEIN |  | 19 | 0.31 | 0.98 | 0.506 | 0.992 | 1.000 | 793 | tags=21%, list=6%, signal=22% |
| 495 | GOBP\_B\_CELL\_ACTIVATION |  | 240 | 0.18 | 0.98 | 0.549 | 0.996 | 1.000 | 2798 | tags=28%, list=22%, signal=35% |
| 496 | GOBP\_PROTEIN\_TRANSMEMBRANE\_IMPORT\_INTO\_INTRACELLULAR\_ORGANELLE |  | 27 | 0.28 | 0.98 | 0.485 | 0.995 | 1.000 | 1253 | tags=19%, list=10%, signal=20% |
| 497 | GOBP\_VITAMIN\_BIOSYNTHETIC\_PROCESS |  | 20 | 0.30 | 0.98 | 0.507 | 0.993 | 1.000 | 2098 | tags=30%, list=16%, signal=36% |
| 498 | GOBP\_SYNCYTIUM\_FORMATION |  | 49 | 0.24 | 0.98 | 0.491 | 0.992 | 1.000 | 804 | tags=14%, list=6%, signal=15% |
| 499 | GOBP\_ANTIGEN\_PROCESSING\_AND\_PRESENTATION\_OF\_PEPTIDE\_OR\_POLYSACCHARIDE\_ANTIGEN\_VIA\_MHC\_CLASS\_II |  | 33 | 0.27 | 0.97 | 0.492 | 0.991 | 1.000 | 2845 | tags=39%, list=22%, signal=50% |
| 500 | GOBP\_GLUCOSAMINE\_CONTAINING\_COMPOUND\_METABOLIC\_PROCESS |  | 22 | 0.29 | 0.97 | 0.499 | 0.990 | 1.000 | 1795 | tags=23%, list=14%, signal=26% |
| 501 | GOBP\_REGULATION\_OF\_RYANODINE\_SENSITIVE\_CALCIUM\_RELEASE\_CHANNEL\_ACTIVITY |  | 20 | 0.30 | 0.97 | 0.483 | 0.994 | 1.000 | 3710 | tags=45%, list=29%, signal=63% |
| 502 | GOBP\_GOLGI\_TO\_VACUOLE\_TRANSPORT |  | 17 | 0.31 | 0.97 | 0.472 | 0.996 | 1.000 | 1756 | tags=24%, list=14%, signal=27% |
| 503 | GOBP\_PINOCYTOSIS |  | 19 | 0.31 | 0.97 | 0.495 | 0.994 | 1.000 | 2313 | tags=37%, list=18%, signal=45% |
| 504 | GOBP\_PYRIDINE\_NUCLEOTIDE\_BIOSYNTHETIC\_PROCESS |  | 16 | 0.32 | 0.97 | 0.466 | 0.994 | 1.000 | 3209 | tags=50%, list=25%, signal=66% |
| 505 | GOBP\_INTRACILIARY\_TRANSPORT |  | 29 | 0.27 | 0.97 | 0.544 | 0.995 | 1.000 | 2135 | tags=24%, list=17%, signal=29% |
| 506 | GOBP\_PROSTANOID\_METABOLIC\_PROCESS |  | 38 | 0.25 | 0.97 | 0.467 | 0.996 | 1.000 | 1996 | tags=26%, list=15%, signal=31% |
| 507 | GOBP\_PROTEIN\_LOCALIZATION\_TO\_LYSOSOME |  | 38 | 0.25 | 0.97 | 0.498 | 0.998 | 1.000 | 3074 | tags=34%, list=24%, signal=45% |
| 508 | GOBP\_HYPEROSMOTIC\_RESPONSE |  | 24 | 0.29 | 0.97 | 0.509 | 0.997 | 1.000 | 3527 | tags=46%, list=27%, signal=63% |
| 509 | GOBP\_MAINTENANCE\_OF\_LOCATION |  | 272 | 0.18 | 0.97 | 0.564 | 0.998 | 1.000 | 2299 | tags=20%, list=18%, signal=24% |
| 510 | GOBP\_POSITIVE\_REGULATION\_OF\_ORGANELLE\_ASSEMBLY |  | 69 | 0.22 | 0.97 | 0.508 | 0.996 | 1.000 | 1766 | tags=17%, list=14%, signal=20% |
| 511 | GOBP\_REGULATION\_OF\_ACTIVIN\_RECEPTOR\_SIGNALING\_PATHWAY |  | 22 | 0.29 | 0.97 | 0.481 | 0.999 | 1.000 | 2293 | tags=27%, list=18%, signal=33% |
| 512 | GOBP\_REACTIVE\_OXYGEN\_SPECIES\_BIOSYNTHETIC\_PROCESS |  | 48 | 0.24 | 0.97 | 0.481 | 1.000 | 1.000 | 2478 | tags=33%, list=19%, signal=41% |
| 513 | GOBP\_INTRACELLULAR\_LIPID\_TRANSPORT |  | 35 | 0.26 | 0.96 | 0.501 | 1.000 | 1.000 | 918 | tags=14%, list=7%, signal=15% |
| 514 | GOBP\_REGULATION\_OF\_SPINDLE\_ASSEMBLY |  | 26 | 0.28 | 0.96 | 0.491 | 1.000 | 1.000 | 2783 | tags=31%, list=22%, signal=39% |
| 515 | GOBP\_RIBONUCLEOSIDE\_MONOPHOSPHATE\_METABOLIC\_PROCESS |  | 48 | 0.24 | 0.96 | 0.493 | 1.000 | 1.000 | 1852 | tags=21%, list=14%, signal=24% |
| 516 | GOBP\_POSITIVE\_REGULATION\_OF\_MICROTUBULE\_POLYMERIZATION\_OR\_DEPOLYMERIZATION |  | 32 | 0.26 | 0.96 | 0.494 | 1.000 | 1.000 | 1502 | tags=19%, list=12%, signal=21% |
| 517 | GOBP\_POSITIVE\_REGULATION\_OF\_NITRIC\_OXIDE\_SYNTHASE\_ACTIVITY |  | 21 | 0.30 | 0.96 | 0.479 | 1.000 | 1.000 | 2776 | tags=33%, list=22%, signal=42% |
| 518 | GOBP\_POSITIVE\_REGULATION\_OF\_CATION\_CHANNEL\_ACTIVITY |  | 54 | 0.23 | 0.96 | 0.543 | 1.000 | 1.000 | 3148 | tags=33%, list=24%, signal=44% |
| 519 | GOBP\_ENDOSOMAL\_TRANSPORT |  | 173 | 0.19 | 0.96 | 0.567 | 1.000 | 1.000 | 2189 | tags=19%, list=17%, signal=23% |
| 520 | GOBP\_REGULATION\_OF\_VIRAL\_ENTRY\_INTO\_HOST\_CELL |  | 37 | 0.25 | 0.96 | 0.488 | 1.000 | 1.000 | 2521 | tags=24%, list=20%, signal=30% |
| 521 | GOBP\_MALE\_MEIOTIC\_NUCLEAR\_DIVISION |  | 35 | 0.25 | 0.96 | 0.525 | 1.000 | 1.000 | 1812 | tags=17%, list=14%, signal=20% |
| 522 | GOBP\_POSITIVE\_REGULATION\_OF\_SYNCYTIUM\_FORMATION\_BY\_PLASMA\_MEMBRANE\_FUSION |  | 21 | 0.29 | 0.96 | 0.490 | 1.000 | 1.000 | 2352 | tags=29%, list=18%, signal=35% |
| 523 | GOBP\_MYOBLAST\_FUSION |  | 35 | 0.26 | 0.96 | 0.545 | 1.000 | 1.000 | 1752 | tags=23%, list=14%, signal=26% |
| 524 | GOBP\_REGULATION\_OF\_BIOLOGICAL\_PROCESS\_INVOLVED\_IN\_SYMBIOTIC\_INTERACTION |  | 49 | 0.23 | 0.96 | 0.518 | 1.000 | 1.000 | 2230 | tags=22%, list=17%, signal=27% |
| 525 | GOBP\_REGULATION\_OF\_INCLUSION\_BODY\_ASSEMBLY |  | 16 | 0.32 | 0.95 | 0.541 | 1.000 | 1.000 | 2083 | tags=31%, list=16%, signal=37% |
| 526 | GOBP\_CELLULAR\_KETONE\_METABOLIC\_PROCESS |  | 175 | 0.19 | 0.95 | 0.606 | 1.000 | 1.000 | 2565 | tags=26%, list=20%, signal=32% |
| 527 | GOBP\_AUTOPHAGOSOME\_ORGANIZATION |  | 85 | 0.21 | 0.95 | 0.559 | 1.000 | 1.000 | 2100 | tags=22%, list=16%, signal=27% |
| 528 | GOBP\_SPERM\_EGG\_RECOGNITION |  | 31 | 0.27 | 0.95 | 0.529 | 1.000 | 1.000 | 315 | tags=10%, list=2%, signal=10% |
| 529 | GOBP\_NEGATIVE\_REGULATION\_OF\_INTERLEUKIN\_1\_PRODUCTION |  | 26 | 0.27 | 0.95 | 0.516 | 1.000 | 1.000 | 2286 | tags=31%, list=18%, signal=37% |
| 530 | GOBP\_NEGATIVE\_REGULATION\_OF\_TELOMERE\_MAINTENANCE\_VIA\_TELOMERASE |  | 15 | 0.32 | 0.95 | 0.495 | 1.000 | 1.000 | 3286 | tags=47%, list=25%, signal=63% |
| 531 | GOBP\_PYRIDINE\_NUCLEOTIDE\_METABOLIC\_PROCESS |  | 19 | 0.29 | 0.95 | 0.541 | 1.000 | 1.000 | 3209 | tags=47%, list=25%, signal=63% |
| 532 | GOBP\_POSITIVE\_REGULATION\_OF\_ION\_TRANSMEMBRANE\_TRANSPORT |  | 133 | 0.19 | 0.95 | 0.556 | 1.000 | 1.000 | 1886 | tags=18%, list=15%, signal=21% |
| 533 | GOBP\_REGULATION\_OF\_IMMUNE\_EFFECTOR\_PROCESS |  | 302 | 0.17 | 0.95 | 0.650 | 1.000 | 1.000 | 2764 | tags=25%, list=21%, signal=30% |
| 534 | GOBP\_RUFFLE\_ORGANIZATION |  | 49 | 0.23 | 0.95 | 0.546 | 1.000 | 1.000 | 2040 | tags=22%, list=16%, signal=27% |
| 535 | GOBP\_OUTFLOW\_TRACT\_SEPTUM\_MORPHOGENESIS |  | 24 | 0.28 | 0.95 | 0.546 | 1.000 | 1.000 | 3958 | tags=50%, list=31%, signal=72% |
| 536 | GOBP\_SECONDARY\_ALCOHOL\_METABOLIC\_PROCESS |  | 129 | 0.20 | 0.95 | 0.603 | 1.000 | 1.000 | 1750 | tags=16%, list=14%, signal=19% |
| 537 | GOBP\_NUCLEOBASE\_METABOLIC\_PROCESS |  | 35 | 0.26 | 0.94 | 0.521 | 1.000 | 1.000 | 1518 | tags=17%, list=12%, signal=19% |
| 538 | GOBP\_SPHINGOLIPID\_METABOLIC\_PROCESS |  | 125 | 0.20 | 0.94 | 0.602 | 1.000 | 1.000 | 2350 | tags=24%, list=18%, signal=29% |
| 539 | GOBP\_MITOCHONDRION\_LOCALIZATION |  | 42 | 0.24 | 0.94 | 0.549 | 1.000 | 1.000 | 1658 | tags=24%, list=13%, signal=27% |
| 540 | GOBP\_REGULATION\_OF\_EXOSOMAL\_SECRETION |  | 19 | 0.30 | 0.94 | 0.556 | 1.000 | 1.000 | 3398 | tags=42%, list=26%, signal=57% |
| 541 | GOBP\_EXTRACELLULAR\_MATRIX\_ASSEMBLY |  | 41 | 0.24 | 0.94 | 0.526 | 1.000 | 1.000 | 2490 | tags=27%, list=19%, signal=33% |
| 542 | GOBP\_SPINDLE\_ORGANIZATION |  | 163 | 0.19 | 0.94 | 0.610 | 1.000 | 1.000 | 3851 | tags=33%, list=30%, signal=46% |
| 543 | GOBP\_REGULATION\_OF\_RESPIRATORY\_GASEOUS\_EXCHANGE |  | 17 | 0.30 | 0.94 | 0.533 | 1.000 | 1.000 | 1314 | tags=24%, list=10%, signal=26% |
| 544 | GOBP\_ESTABLISHMENT\_OF\_PROTEIN\_LOCALIZATION\_TO\_PLASMA\_MEMBRANE |  | 50 | 0.23 | 0.94 | 0.578 | 1.000 | 1.000 | 2370 | tags=22%, list=18%, signal=27% |
| 545 | GOBP\_NUCLEAR\_TRANSCRIBED\_MRNA\_CATABOLIC\_PROCESS\_EXONUCLEOLYTIC |  | 15 | 0.30 | 0.94 | 0.533 | 1.000 | 1.000 | 2875 | tags=40%, list=22%, signal=51% |
| 546 | GOBP\_REGULATION\_OF\_PROTEIN\_LOCALIZATION\_TO\_PLASMA\_MEMBRANE |  | 81 | 0.21 | 0.94 | 0.601 | 1.000 | 1.000 | 1821 | tags=21%, list=14%, signal=24% |
| 547 | GOBP\_REGULATION\_OF\_MITOCHONDRIAL\_MEMBRANE\_POTENTIAL |  | 60 | 0.22 | 0.94 | 0.585 | 1.000 | 1.000 | 2640 | tags=32%, list=20%, signal=40% |
| 548 | GOBP\_REGULATION\_OF\_CELLULAR\_RESPIRATION |  | 43 | 0.24 | 0.94 | 0.563 | 1.000 | 1.000 | 2517 | tags=28%, list=20%, signal=35% |
| 549 | GOBP\_REGULATION\_OF\_NEUROTRANSMITTER\_TRANSPORT |  | 85 | 0.20 | 0.94 | 0.601 | 1.000 | 1.000 | 1972 | tags=20%, list=15%, signal=23% |
| 550 | GOBP\_RESPONSE\_TO\_METAL\_ION |  | 302 | 0.17 | 0.94 | 0.711 | 1.000 | 1.000 | 3234 | tags=30%, list=25%, signal=39% |
| 551 | GOBP\_REGULATION\_OF\_PHAGOCYTOSIS |  | 86 | 0.20 | 0.93 | 0.568 | 1.000 | 1.000 | 2764 | tags=27%, list=21%, signal=34% |
| 552 | GOBP\_EXTRACELLULAR\_VESICLE\_BIOGENESIS |  | 23 | 0.28 | 0.93 | 0.547 | 1.000 | 1.000 | 3398 | tags=39%, list=26%, signal=53% |
| 553 | GOBP\_RESPONSE\_TO\_ISOQUINOLINE\_ALKALOID |  | 25 | 0.27 | 0.93 | 0.570 | 1.000 | 1.000 | 1462 | tags=16%, list=11%, signal=18% |
| 554 | GOBP\_FATTY\_ACID\_BIOSYNTHETIC\_PROCESS |  | 126 | 0.19 | 0.93 | 0.636 | 1.000 | 1.000 | 2017 | tags=20%, list=16%, signal=23% |
| 555 | GOBP\_LAMELLIPODIUM\_MORPHOGENESIS |  | 17 | 0.31 | 0.93 | 0.559 | 1.000 | 1.000 | 279 | tags=12%, list=2%, signal=12% |
| 556 | GOBP\_CYTOKINESIS |  | 135 | 0.19 | 0.93 | 0.654 | 1.000 | 1.000 | 1528 | tags=18%, list=12%, signal=20% |
| 557 | GOBP\_RRNA\_METHYLATION |  | 20 | 0.29 | 0.93 | 0.560 | 1.000 | 1.000 | 2107 | tags=25%, list=16%, signal=30% |
| 558 | GOBP\_RESPONSE\_TO\_COCAINE |  | 43 | 0.24 | 0.93 | 0.578 | 1.000 | 1.000 | 1251 | tags=16%, list=10%, signal=18% |
| 559 | GOBP\_ORGANIC\_CATION\_TRANSPORT |  | 27 | 0.26 | 0.93 | 0.563 | 1.000 | 1.000 | 785 | tags=15%, list=6%, signal=16% |
| 560 | GOBP\_CYTOSOLIC\_TRANSPORT |  | 134 | 0.19 | 0.93 | 0.669 | 1.000 | 1.000 | 3037 | tags=28%, list=24%, signal=36% |
| 561 | GOBP\_VENTRAL\_SPINAL\_CORD\_DEVELOPMENT |  | 33 | 0.26 | 0.93 | 0.595 | 1.000 | 1.000 | 4227 | tags=39%, list=33%, signal=58% |
| 562 | GOBP\_SNRNA\_TRANSCRIPTION\_BY\_RNA\_POLYMERASE\_II |  | 15 | 0.31 | 0.93 | 0.547 | 1.000 | 1.000 | 3395 | tags=40%, list=26%, signal=54% |
| 563 | GOBP\_REGULATION\_OF\_RECEPTOR\_BINDING |  | 18 | 0.29 | 0.93 | 0.536 | 1.000 | 1.000 | 1136 | tags=22%, list=9%, signal=24% |
| 564 | GOBP\_PROTEIN\_LOCALIZATION\_TO\_VACUOLE |  | 53 | 0.23 | 0.93 | 0.609 | 1.000 | 1.000 | 3074 | tags=34%, list=24%, signal=44% |
| 565 | GOBP\_DNA\_METHYLATION\_DEPENDENT\_HETEROCHROMATIN\_ASSEMBLY |  | 19 | 0.29 | 0.93 | 0.577 | 1.000 | 1.000 | 2836 | tags=32%, list=22%, signal=40% |
| 566 | GOBP\_REGULATION\_OF\_HUMORAL\_IMMUNE\_RESPONSE\_MEDIATED\_BY\_CIRCULATING\_IMMUNOGLOBULIN |  | 17 | 0.30 | 0.92 | 0.564 | 1.000 | 1.000 | 3785 | tags=53%, list=29%, signal=75% |
| 567 | GOBP\_POSITIVE\_REGULATION\_OF\_NATURAL\_KILLER\_CELL\_ACTIVATION |  | 21 | 0.28 | 0.92 | 0.549 | 1.000 | 1.000 | 2599 | tags=33%, list=20%, signal=42% |
| 568 | GOBP\_NEUROMUSCULAR\_PROCESS |  | 120 | 0.19 | 0.92 | 0.662 | 1.000 | 1.000 | 2877 | tags=27%, list=22%, signal=34% |
| 569 | GOBP\_IMMUNE\_RESPONSE\_REGULATING\_SIGNALING\_PATHWAY |  | 357 | 0.17 | 0.92 | 0.758 | 1.000 | 1.000 | 2757 | tags=25%, list=21%, signal=31% |
| 570 | GOBP\_DEFENSE\_RESPONSE\_TO\_SYMBIONT |  | 233 | 0.17 | 0.92 | 0.725 | 1.000 | 1.000 | 2619 | tags=23%, list=20%, signal=29% |
| 571 | GOBP\_O\_GLYCAN\_PROCESSING |  | 30 | 0.26 | 0.92 | 0.589 | 1.000 | 1.000 | 2035 | tags=23%, list=16%, signal=28% |
| 572 | GOBP\_STEROID\_METABOLIC\_PROCESS |  | 273 | 0.17 | 0.92 | 0.722 | 1.000 | 1.000 | 2117 | tags=18%, list=16%, signal=21% |
| 573 | GOBP\_REGULATION\_OF\_MICROTUBULE\_CYTOSKELETON\_ORGANIZATION |  | 133 | 0.19 | 0.92 | 0.678 | 1.000 | 1.000 | 1907 | tags=19%, list=15%, signal=22% |
| 574 | GOBP\_PROTEIN\_LOCALIZATION\_TO\_CELL\_SURFACE |  | 58 | 0.22 | 0.92 | 0.634 | 1.000 | 1.000 | 2877 | tags=31%, list=22%, signal=40% |
| 575 | GOBP\_REGULATION\_OF\_MONOOXYGENASE\_ACTIVITY |  | 47 | 0.23 | 0.92 | 0.601 | 1.000 | 1.000 | 2337 | tags=26%, list=18%, signal=31% |
| 576 | GOBP\_CELL\_COMMUNICATION\_INVOLVED\_IN\_CARDIAC\_CONDUCTION |  | 49 | 0.23 | 0.92 | 0.628 | 1.000 | 1.000 | 1177 | tags=16%, list=9%, signal=18% |
| 577 | GOBP\_LONG\_CHAIN\_FATTY\_ACYL\_COA\_METABOLIC\_PROCESS |  | 19 | 0.28 | 0.92 | 0.583 | 1.000 | 1.000 | 3253 | tags=37%, list=25%, signal=49% |
| 578 | GOBP\_PROTEIN\_LOCALIZATION\_TO\_PHAGOPHORE\_ASSEMBLY\_SITE |  | 15 | 0.30 | 0.92 | 0.564 | 1.000 | 1.000 | 1612 | tags=27%, list=12%, signal=30% |
| 579 | GOBP\_REGULATION\_OF\_CHOLESTEROL\_METABOLIC\_PROCESS |  | 29 | 0.25 | 0.92 | 0.577 | 1.000 | 1.000 | 2776 | tags=31%, list=22%, signal=39% |
| 580 | GOBP\_ESTABLISHMENT\_OF\_MITOCHONDRION\_LOCALIZATION |  | 27 | 0.26 | 0.92 | 0.586 | 1.000 | 1.000 | 1658 | tags=26%, list=13%, signal=30% |
| 581 | GOBP\_CELLULAR\_RESPONSE\_TO\_VITAMIN |  | 30 | 0.26 | 0.91 | 0.587 | 1.000 | 1.000 | 2670 | tags=30%, list=21%, signal=38% |
| 582 | GOBP\_TOXIN\_METABOLIC\_PROCESS |  | 16 | 0.30 | 0.91 | 0.567 | 1.000 | 1.000 | 330 | tags=13%, list=3%, signal=13% |
| 583 | GOBP\_PROTEIN\_DESTABILIZATION |  | 43 | 0.23 | 0.91 | 0.619 | 1.000 | 1.000 | 2008 | tags=26%, list=16%, signal=30% |
| 584 | GOBP\_REGULATION\_OF\_VASCULAR\_ENDOTHELIAL\_GROWTH\_FACTOR\_RECEPTOR\_SIGNALING\_PATHWAY |  | 23 | 0.27 | 0.91 | 0.595 | 1.000 | 1.000 | 969 | tags=17%, list=8%, signal=19% |
| 585 | GOBP\_REGULATION\_OF\_COMPLEMENT\_ACTIVATION |  | 19 | 0.29 | 0.91 | 0.577 | 1.000 | 1.000 | 3435 | tags=42%, list=27%, signal=57% |
| 586 | GOBP\_POSITIVE\_REGULATION\_OF\_IMMUNOGLOBULIN\_PRODUCTION |  | 43 | 0.24 | 0.91 | 0.648 | 1.000 | 1.000 | 1199 | tags=14%, list=9%, signal=15% |
| 587 | GOBP\_AUTOPHAGY\_OF\_NUCLEUS |  | 15 | 0.31 | 0.91 | 0.559 | 1.000 | 1.000 | 1612 | tags=20%, list=12%, signal=23% |
| 588 | GOBP\_NUCLEAR\_ENVELOPE\_ORGANIZATION |  | 48 | 0.23 | 0.91 | 0.631 | 1.000 | 1.000 | 2733 | tags=33%, list=21%, signal=42% |
| 589 | GOBP\_IMMUNOGLOBULIN\_PRODUCTION\_INVOLVED\_IN\_IMMUNOGLOBULIN\_MEDIATED\_IMMUNE\_RESPONSE |  | 59 | 0.21 | 0.91 | 0.622 | 1.000 | 1.000 | 2948 | tags=29%, list=23%, signal=37% |
| 590 | GOBP\_FATTY\_ACYL\_COA\_METABOLIC\_PROCESS |  | 27 | 0.26 | 0.90 | 0.600 | 1.000 | 1.000 | 3253 | tags=33%, list=25%, signal=44% |
| 591 | GOBP\_MICROTUBULE\_CYTOSKELETON\_ORGANIZATION\_INVOLVED\_IN\_MITOSIS |  | 132 | 0.19 | 0.90 | 0.707 | 1.000 | 1.000 | 3696 | tags=32%, list=29%, signal=44% |
| 592 | GOBP\_LEUKOCYTE\_MEDIATED\_CYTOTOXICITY |  | 114 | 0.19 | 0.90 | 0.690 | 1.000 | 1.000 | 3572 | tags=36%, list=28%, signal=49% |
| 593 | GOBP\_CELLULAR\_CARBOHYDRATE\_BIOSYNTHETIC\_PROCESS |  | 65 | 0.21 | 0.90 | 0.644 | 1.000 | 1.000 | 1587 | tags=18%, list=12%, signal=21% |
| 594 | GOBP\_REGULATION\_OF\_PROTEIN\_LOCALIZATION\_TO\_MEMBRANE |  | 139 | 0.18 | 0.90 | 0.751 | 1.000 | 1.000 | 1677 | tags=18%, list=13%, signal=20% |
| 595 | GOBP\_LEUKOTRIENE\_METABOLIC\_PROCESS |  | 26 | 0.26 | 0.90 | 0.627 | 1.000 | 1.000 | 610 | tags=12%, list=5%, signal=12% |
| 596 | GOBP\_ACTIN\_NUCLEATION |  | 40 | 0.23 | 0.90 | 0.613 | 1.000 | 1.000 | 2252 | tags=28%, list=17%, signal=33% |
| 597 | GOBP\_REGULATION\_OF\_CARDIAC\_MUSCLE\_CONTRACTION\_BY\_CALCIUM\_ION\_SIGNALING |  | 21 | 0.27 | 0.90 | 0.630 | 1.000 | 1.000 | 1214 | tags=19%, list=9%, signal=21% |
| 598 | GOBP\_RESPONSE\_TO\_OXYGEN\_RADICAL |  | 23 | 0.27 | 0.90 | 0.618 | 1.000 | 1.000 | 3113 | tags=35%, list=24%, signal=46% |
| 599 | GOBP\_REGULATION\_OF\_CELL\_FATE\_COMMITMENT |  | 19 | 0.28 | 0.90 | 0.600 | 1.000 | 1.000 | 2064 | tags=26%, list=16%, signal=31% |
| 600 | GOBP\_POSITIVE\_REGULATION\_OF\_STEM\_CELL\_DIFFERENTIATION |  | 15 | 0.30 | 0.90 | 0.590 | 1.000 | 1.000 | 2089 | tags=27%, list=16%, signal=32% |
| 601 | GOBP\_PROTEIN\_TARGETING\_TO\_LYSOSOME |  | 21 | 0.27 | 0.90 | 0.615 | 1.000 | 1.000 | 3481 | tags=43%, list=27%, signal=59% |
| 602 | GOBP\_METHIONINE\_METABOLIC\_PROCESS |  | 15 | 0.30 | 0.90 | 0.626 | 1.000 | 1.000 | 643 | tags=13%, list=5%, signal=14% |
| 603 | GOBP\_REGULATION\_OF\_REACTIVE\_OXYGEN\_SPECIES\_BIOSYNTHETIC\_PROCESS |  | 35 | 0.25 | 0.89 | 0.634 | 1.000 | 1.000 | 1800 | tags=26%, list=14%, signal=30% |
| 604 | GOBP\_NEGATIVE\_REGULATION\_OF\_PEPTIDYL\_TYROSINE\_PHOSPHORYLATION |  | 44 | 0.23 | 0.89 | 0.628 | 1.000 | 1.000 | 2503 | tags=30%, list=19%, signal=37% |
| 605 | GOBP\_NEGATIVE\_REGULATION\_OF\_PROTEIN\_TYROSINE\_KINASE\_ACTIVITY |  | 24 | 0.26 | 0.89 | 0.635 | 1.000 | 1.000 | 2275 | tags=29%, list=18%, signal=35% |
| 606 | GOBP\_REGULATION\_OF\_MICROTUBULE\_POLYMERIZATION |  | 47 | 0.22 | 0.89 | 0.667 | 1.000 | 1.000 | 1389 | tags=17%, list=11%, signal=19% |
| 607 | GOBP\_REGULATION\_OF\_ANIMAL\_ORGAN\_MORPHOGENESIS |  | 99 | 0.19 | 0.89 | 0.709 | 1.000 | 1.000 | 2745 | tags=29%, list=21%, signal=37% |
| 608 | GOBP\_RENAL\_SYSTEM\_PROCESS\_INVOLVED\_IN\_REGULATION\_OF\_BLOOD\_VOLUME |  | 15 | 0.29 | 0.89 | 0.598 | 1.000 | 1.000 | 539 | tags=20%, list=4%, signal=21% |
| 609 | GOBP\_REPRODUCTIVE\_BEHAVIOR |  | 23 | 0.27 | 0.89 | 0.631 | 1.000 | 1.000 | 1958 | tags=22%, list=15%, signal=26% |
| 610 | GOBP\_REGULATION\_OF\_PROTEIN\_TARGETING\_TO\_MEMBRANE |  | 30 | 0.25 | 0.89 | 0.625 | 1.000 | 1.000 | 963 | tags=20%, list=7%, signal=22% |
| 611 | GOBP\_REGULATION\_OF\_CILIUM\_DEPENDENT\_CELL\_MOTILITY |  | 16 | 0.29 | 0.89 | 0.646 | 1.000 | 1.000 | 3527 | tags=44%, list=27%, signal=60% |
| 612 | GOBP\_RUFFLE\_ASSEMBLY |  | 37 | 0.24 | 0.89 | 0.648 | 1.000 | 1.000 | 2040 | tags=24%, list=16%, signal=29% |
| 613 | GOBP\_NEGATIVE\_REGULATION\_OF\_POTASSIUM\_ION\_TRANSMEMBRANE\_TRANSPORT |  | 20 | 0.27 | 0.89 | 0.633 | 1.000 | 1.000 | 4181 | tags=45%, list=32%, signal=66% |
| 614 | GOBP\_NEGATIVE\_REGULATION\_OF\_PROTEASOMAL\_PROTEIN\_CATABOLIC\_PROCESS |  | 39 | 0.23 | 0.88 | 0.663 | 1.000 | 1.000 | 1406 | tags=15%, list=11%, signal=17% |
| 615 | GOBP\_TERTIARY\_ALCOHOL\_METABOLIC\_PROCESS |  | 20 | 0.27 | 0.88 | 0.641 | 1.000 | 1.000 | 3138 | tags=40%, list=24%, signal=53% |
| 616 | GOBP\_DIOL\_BIOSYNTHETIC\_PROCESS |  | 15 | 0.30 | 0.88 | 0.616 | 1.000 | 1.000 | 3405 | tags=47%, list=26%, signal=63% |
| 617 | GOBP\_VENTRICULAR\_CARDIAC\_MUSCLE\_CELL\_MEMBRANE\_REPOLARIZATION |  | 21 | 0.27 | 0.88 | 0.639 | 1.000 | 1.000 | 2078 | tags=19%, list=16%, signal=23% |
| 618 | GOBP\_PYRIMIDINE\_DEOXYRIBONUCLEOTIDE\_METABOLIC\_PROCESS |  | 21 | 0.27 | 0.88 | 0.652 | 1.000 | 1.000 | 4044 | tags=52%, list=31%, signal=76% |
| 619 | GOBP\_PROTEIN\_HOMOOLIGOMERIZATION |  | 141 | 0.18 | 0.88 | 0.799 | 1.000 | 1.000 | 3163 | tags=28%, list=25%, signal=37% |
| 620 | GOBP\_POSITIVE\_REGULATION\_OF\_LEUKOCYTE\_PROLIFERATION |  | 132 | 0.18 | 0.88 | 0.785 | 1.000 | 1.000 | 2599 | tags=23%, list=20%, signal=29% |
| 621 | GOBP\_RETINOL\_METABOLIC\_PROCESS |  | 39 | 0.23 | 0.88 | 0.672 | 1.000 | 1.000 | 308 | tags=8%, list=2%, signal=8% |
| 622 | GOBP\_NEURAL\_NUCLEUS\_DEVELOPMENT |  | 54 | 0.21 | 0.88 | 0.738 | 1.000 | 1.000 | 2192 | tags=22%, list=17%, signal=27% |
| 623 | GOBP\_CELLULAR\_TRANSITION\_METAL\_ION\_HOMEOSTASIS |  | 81 | 0.20 | 0.88 | 0.743 | 1.000 | 1.000 | 2568 | tags=26%, list=20%, signal=32% |
| 624 | GOBP\_AMYLOID\_FIBRIL\_FORMATION |  | 31 | 0.24 | 0.88 | 0.652 | 1.000 | 1.000 | 3192 | tags=39%, list=25%, signal=51% |
| 625 | GOBP\_TRANSPOSITION |  | 24 | 0.26 | 0.88 | 0.644 | 1.000 | 1.000 | 1917 | tags=25%, list=15%, signal=29% |
| 626 | GOBP\_BASEMENT\_MEMBRANE\_ORGANIZATION |  | 28 | 0.25 | 0.88 | 0.662 | 1.000 | 1.000 | 3687 | tags=46%, list=29%, signal=65% |
| 627 | GOBP\_POSITIVE\_REGULATION\_OF\_IMMUNE\_RESPONSE |  | 448 | 0.16 | 0.88 | 0.921 | 1.000 | 1.000 | 2764 | tags=24%, list=21%, signal=30% |
| 628 | GOBP\_RESPONSE\_TO\_UV\_B |  | 15 | 0.29 | 0.88 | 0.643 | 1.000 | 1.000 | 997 | tags=20%, list=8%, signal=22% |
| 629 | GOBP\_FC\_RECEPTOR\_SIGNALING\_PATHWAY |  | 47 | 0.22 | 0.88 | 0.724 | 1.000 | 1.000 | 2237 | tags=26%, list=17%, signal=31% |
| 630 | GOBP\_VESICLE\_ORGANIZATION |  | 236 | 0.16 | 0.88 | 0.837 | 1.000 | 1.000 | 2901 | tags=23%, list=22%, signal=30% |
| 631 | GOBP\_POSITIVE\_REGULATION\_OF\_STEROL\_TRANSPORT |  | 32 | 0.24 | 0.88 | 0.677 | 1.000 | 1.000 | 1728 | tags=19%, list=13%, signal=22% |
| 632 | GOBP\_PYRIDINE\_CONTAINING\_COMPOUND\_METABOLIC\_PROCESS |  | 25 | 0.25 | 0.87 | 0.647 | 1.000 | 1.000 | 3324 | tags=44%, list=26%, signal=59% |
| 633 | GOBP\_NEGATIVE\_REGULATION\_OF\_JNK\_CASCADE |  | 31 | 0.24 | 0.87 | 0.670 | 1.000 | 1.000 | 2549 | tags=32%, list=20%, signal=40% |
| 634 | GOBP\_DEFENSE\_RESPONSE\_TO\_GRAM\_NEGATIVE\_BACTERIUM |  | 56 | 0.21 | 0.87 | 0.697 | 1.000 | 1.000 | 3291 | tags=34%, list=26%, signal=45% |
| 635 | GOBP\_SECRETION\_BY\_TISSUE |  | 34 | 0.24 | 0.87 | 0.698 | 1.000 | 1.000 | 1959 | tags=21%, list=15%, signal=24% |
| 636 | GOBP\_NADH\_METABOLIC\_PROCESS |  | 26 | 0.25 | 0.87 | 0.653 | 1.000 | 1.000 | 759 | tags=12%, list=6%, signal=12% |
| 637 | GOBP\_SECONDARY\_PALATE\_DEVELOPMENT |  | 23 | 0.26 | 0.87 | 0.652 | 1.000 | 1.000 | 1111 | tags=13%, list=9%, signal=14% |
| 638 | GOBP\_POSITIVE\_REGULATION\_OF\_T\_CELL\_MIGRATION |  | 26 | 0.25 | 0.87 | 0.645 | 1.000 | 1.000 | 1995 | tags=31%, list=15%, signal=36% |
| 639 | GOBP\_NEGATIVE\_REGULATION\_OF\_LEUKOCYTE\_MEDIATED\_IMMUNITY |  | 61 | 0.20 | 0.87 | 0.714 | 1.000 | 1.000 | 2018 | tags=21%, list=16%, signal=25% |
| 640 | GOBP\_LIPID\_OXIDATION |  | 97 | 0.19 | 0.87 | 0.792 | 1.000 | 1.000 | 1453 | tags=16%, list=11%, signal=18% |
| 641 | GOBP\_RESPONSE\_TO\_CADMIUM\_ION |  | 53 | 0.21 | 0.87 | 0.707 | 1.000 | 1.000 | 2984 | tags=34%, list=23%, signal=44% |
| 642 | GOBP\_NAD\_METABOLIC\_PROCESS |  | 26 | 0.25 | 0.87 | 0.650 | 1.000 | 1.000 | 4002 | tags=38%, list=31%, signal=56% |
| 643 | GOBP\_POSITIVE\_REGULATION\_OF\_TRANSPORTER\_ACTIVITY |  | 95 | 0.19 | 0.87 | 0.754 | 1.000 | 1.000 | 3148 | tags=29%, list=24%, signal=39% |
| 644 | GOBP\_REGULATION\_OF\_MITOCHONDRIAL\_MEMBRANE\_PERMEABILITY\_INVOLVED\_IN\_APOPTOTIC\_PROCESS |  | 32 | 0.24 | 0.87 | 0.686 | 1.000 | 1.000 | 1610 | tags=19%, list=12%, signal=21% |
| 645 | GOBP\_INCLUSION\_BODY\_ASSEMBLY |  | 22 | 0.27 | 0.87 | 0.677 | 1.000 | 1.000 | 2083 | tags=27%, list=16%, signal=32% |
| 646 | GOBP\_REGULATION\_OF\_HAIR\_FOLLICLE\_DEVELOPMENT |  | 18 | 0.28 | 0.87 | 0.654 | 1.000 | 1.000 | 2360 | tags=22%, list=18%, signal=27% |
| 647 | GOBP\_HUMORAL\_IMMUNE\_RESPONSE |  | 226 | 0.17 | 0.86 | 0.907 | 1.000 | 1.000 | 3449 | tags=34%, list=27%, signal=46% |
| 648 | GOBP\_MITOCHONDRION\_MORPHOGENESIS |  | 19 | 0.27 | 0.86 | 0.663 | 1.000 | 1.000 | 932 | tags=16%, list=7%, signal=17% |
| 649 | GOBP\_REGULATION\_OF\_MYELOID\_CELL\_APOPTOTIC\_PROCESS |  | 25 | 0.25 | 0.86 | 0.683 | 1.000 | 1.000 | 3502 | tags=40%, list=27%, signal=55% |
| 650 | GOBP\_AMINOGLYCAN\_METABOLIC\_PROCESS |  | 103 | 0.19 | 0.86 | 0.767 | 1.000 | 1.000 | 3564 | tags=36%, list=28%, signal=49% |
| 651 | GOBP\_REGULATION\_OF\_PROTEIN\_LOCALIZATION\_TO\_CELL\_PERIPHERY |  | 99 | 0.19 | 0.86 | 0.778 | 1.000 | 1.000 | 2592 | tags=24%, list=20%, signal=30% |
| 652 | GOBP\_EXOCYTIC\_PROCESS |  | 57 | 0.20 | 0.86 | 0.733 | 1.000 | 1.000 | 889 | tags=12%, list=7%, signal=13% |
| 653 | GOBP\_PROSTANOID\_BIOSYNTHETIC\_PROCESS |  | 25 | 0.25 | 0.86 | 0.682 | 1.000 | 1.000 | 605 | tags=16%, list=5%, signal=17% |
| 654 | GOBP\_NEGATIVE\_REGULATION\_OF\_PROTEIN\_LOCALIZATION\_TO\_CELL\_PERIPHERY |  | 20 | 0.27 | 0.86 | 0.692 | 1.000 | 1.000 | 2592 | tags=35%, list=20%, signal=44% |
| 655 | GOBP\_TRNA\_MODIFICATION |  | 57 | 0.21 | 0.86 | 0.729 | 1.000 | 1.000 | 1605 | tags=18%, list=12%, signal=20% |
| 656 | GOBP\_ACTIVATION\_OF\_CYSTEINE\_TYPE\_ENDOPEPTIDASE\_ACTIVITY\_INVOLVED\_IN\_APOPTOTIC\_PROCESS |  | 73 | 0.20 | 0.86 | 0.785 | 1.000 | 1.000 | 1838 | tags=18%, list=14%, signal=21% |
| 657 | GOBP\_DETECTION\_OF\_MECHANICAL\_STIMULUS\_INVOLVED\_IN\_SENSORY\_PERCEPTION |  | 25 | 0.25 | 0.86 | 0.697 | 1.000 | 1.000 | 3015 | tags=28%, list=23%, signal=36% |
| 658 | GOBP\_POSITIVE\_REGULATION\_OF\_MONONUCLEAR\_CELL\_MIGRATION |  | 58 | 0.20 | 0.86 | 0.777 | 1.000 | 1.000 | 2098 | tags=24%, list=16%, signal=29% |
| 659 | GOBP\_HEPARAN\_SULFATE\_PROTEOGLYCAN\_METABOLIC\_PROCESS |  | 25 | 0.25 | 0.86 | 0.692 | 1.000 | 1.000 | 3497 | tags=48%, list=27%, signal=66% |
| 660 | GOBP\_POSITIVE\_REGULATION\_OF\_NEURON\_PROJECTION\_DEVELOPMENT |  | 127 | 0.18 | 0.86 | 0.826 | 1.000 | 1.000 | 3029 | tags=29%, list=23%, signal=38% |
| 661 | GOBP\_REGULATION\_OF\_CHROMOSOME\_SEGREGATION |  | 70 | 0.20 | 0.86 | 0.772 | 1.000 | 1.000 | 3773 | tags=36%, list=29%, signal=50% |
| 662 | GOBP\_FOLIC\_ACID\_CONTAINING\_COMPOUND\_METABOLIC\_PROCESS |  | 23 | 0.26 | 0.86 | 0.703 | 1.000 | 1.000 | 2284 | tags=22%, list=18%, signal=26% |
| 663 | GOBP\_POSITIVE\_REGULATION\_OF\_MONOOXYGENASE\_ACTIVITY |  | 31 | 0.24 | 0.86 | 0.696 | 1.000 | 1.000 | 2186 | tags=26%, list=17%, signal=31% |
| 664 | GOBP\_CELLULAR\_RESPONSE\_TO\_LIPOPROTEIN\_PARTICLE\_STIMULUS |  | 30 | 0.24 | 0.86 | 0.703 | 1.000 | 1.000 | 1752 | tags=23%, list=14%, signal=27% |
| 665 | GOBP\_TOXIN\_TRANSPORT |  | 33 | 0.23 | 0.85 | 0.708 | 1.000 | 1.000 | 4435 | tags=48%, list=34%, signal=74% |
| 666 | GOBP\_AMYLOID\_BETA\_FORMATION |  | 41 | 0.22 | 0.85 | 0.700 | 1.000 | 1.000 | 3383 | tags=39%, list=26%, signal=53% |
| 667 | GOBP\_DORSAL\_VENTRAL\_PATTERN\_FORMATION |  | 66 | 0.20 | 0.85 | 0.756 | 1.000 | 1.000 | 2003 | tags=20%, list=16%, signal=23% |
| 668 | GOBP\_CARBOHYDRATE\_DERIVATIVE\_TRANSPORT |  | 60 | 0.20 | 0.85 | 0.713 | 1.000 | 1.000 | 2624 | tags=27%, list=20%, signal=33% |
| 669 | GOBP\_GLIAL\_CELL\_APOPTOTIC\_PROCESS |  | 15 | 0.30 | 0.85 | 0.632 | 1.000 | 1.000 | 3728 | tags=47%, list=29%, signal=66% |
| 670 | GOBP\_CYTOLYSIS |  | 24 | 0.25 | 0.85 | 0.687 | 1.000 | 1.000 | 2490 | tags=25%, list=19%, signal=31% |
| 671 | GOBP\_COLLAGEN\_FIBRIL\_ORGANIZATION |  | 53 | 0.20 | 0.85 | 0.753 | 1.000 | 1.000 | 1710 | tags=17%, list=13%, signal=19% |
| 672 | GOBP\_PROTEIN\_HOMOTETRAMERIZATION |  | 52 | 0.21 | 0.85 | 0.728 | 1.000 | 1.000 | 1783 | tags=17%, list=14%, signal=20% |
| 673 | GOBP\_APOPTOTIC\_CELL\_CLEARANCE |  | 42 | 0.22 | 0.85 | 0.774 | 1.000 | 1.000 | 2884 | tags=33%, list=22%, signal=43% |
| 674 | GOBP\_INTERFERON\_GAMMA\_PRODUCTION |  | 97 | 0.18 | 0.85 | 0.786 | 1.000 | 1.000 | 3599 | tags=35%, list=28%, signal=48% |
| 675 | GOBP\_REGULATION\_OF\_OXIDATIVE\_PHOSPHORYLATION |  | 21 | 0.26 | 0.85 | 0.675 | 1.000 | 1.000 | 2517 | tags=33%, list=20%, signal=41% |
| 676 | GOBP\_POSITIVE\_REGULATION\_OF\_CELL\_ACTIVATION |  | 327 | 0.16 | 0.85 | 0.938 | 1.000 | 1.000 | 2861 | tags=26%, list=22%, signal=32% |
| 677 | GOBP\_REGULATION\_OF\_RECEPTOR\_RECYCLING |  | 19 | 0.27 | 0.85 | 0.659 | 1.000 | 1.000 | 3020 | tags=42%, list=23%, signal=55% |
| 678 | GOBP\_SLEEP |  | 25 | 0.24 | 0.84 | 0.712 | 1.000 | 1.000 | 4133 | tags=48%, list=32%, signal=70% |
| 679 | GOBP\_CHOLESTEROL\_STORAGE |  | 17 | 0.27 | 0.84 | 0.696 | 1.000 | 1.000 | 1728 | tags=24%, list=13%, signal=27% |
| 680 | GOBP\_RRNA\_MODIFICATION |  | 27 | 0.23 | 0.84 | 0.726 | 1.000 | 1.000 | 2107 | tags=26%, list=16%, signal=31% |
| 681 | GOBP\_FEMALE\_MEIOTIC\_NUCLEAR\_DIVISION |  | 23 | 0.25 | 0.84 | 0.683 | 1.000 | 1.000 | 1027 | tags=13%, list=8%, signal=14% |
| 682 | GOBP\_PROTEIN\_TETRAMERIZATION |  | 81 | 0.19 | 0.84 | 0.847 | 1.000 | 1.000 | 1978 | tags=20%, list=15%, signal=23% |
| 683 | GOBP\_DNA\_DEALKYLATION |  | 23 | 0.25 | 0.84 | 0.728 | 1.000 | 1.000 | 2695 | tags=35%, list=21%, signal=44% |
| 684 | GOBP\_REGULATION\_OF\_LONG\_TERM\_SYNAPTIC\_POTENTIATION |  | 38 | 0.22 | 0.84 | 0.772 | 1.000 | 1.000 | 765 | tags=11%, list=6%, signal=11% |
| 685 | GOBP\_SOMATIC\_DIVERSIFICATION\_OF\_IMMUNOGLOBULINS |  | 59 | 0.20 | 0.84 | 0.792 | 1.000 | 1.000 | 2095 | tags=17%, list=16%, signal=20% |
| 686 | GOBP\_SCF\_DEPENDENT\_PROTEASOMAL\_UBIQUITIN\_DEPENDENT\_PROTEIN\_CATABOLIC\_PROCESS |  | 30 | 0.23 | 0.84 | 0.730 | 1.000 | 1.000 | 1736 | tags=20%, list=13%, signal=23% |
| 687 | GOBP\_REGULATION\_OF\_BRANCHING\_INVOLVED\_IN\_URETERIC\_BUD\_MORPHOGENESIS |  | 18 | 0.27 | 0.84 | 0.707 | 1.000 | 1.000 | 2089 | tags=28%, list=16%, signal=33% |
| 688 | GOBP\_NEGATIVE\_REGULATION\_OF\_PROTEIN\_LOCALIZATION\_TO\_MEMBRANE |  | 26 | 0.24 | 0.83 | 0.716 | 1.000 | 1.000 | 2592 | tags=31%, list=20%, signal=38% |
| 689 | GOBP\_REGULATION\_OF\_CYSTEINE\_TYPE\_ENDOPEPTIDASE\_ACTIVITY\_INVOLVED\_IN\_APOPTOTIC\_SIGNALING\_PATHWAY |  | 17 | 0.26 | 0.83 | 0.693 | 1.000 | 1.000 | 4183 | tags=47%, list=32%, signal=70% |
| 690 | GOBP\_LACTATION |  | 38 | 0.22 | 0.83 | 0.770 | 1.000 | 1.000 | 3043 | tags=29%, list=24%, signal=38% |
| 691 | GOBP\_RESPONSE\_TO\_GROWTH\_HORMONE |  | 33 | 0.23 | 0.83 | 0.759 | 1.000 | 1.000 | 2466 | tags=27%, list=19%, signal=34% |
| 692 | GOBP\_REGULATION\_OF\_CELLULAR\_PH |  | 62 | 0.19 | 0.83 | 0.799 | 1.000 | 1.000 | 2148 | tags=21%, list=17%, signal=25% |
| 693 | GOBP\_SYNAPTIC\_VESICLE\_MEMBRANE\_ORGANIZATION |  | 19 | 0.26 | 0.83 | 0.700 | 1.000 | 1.000 | 650 | tags=11%, list=5%, signal=11% |
| 694 | GOBP\_RESPONSE\_TO\_HYPEROXIA |  | 18 | 0.26 | 0.83 | 0.686 | 1.000 | 1.000 | 2387 | tags=22%, list=19%, signal=27% |
| 695 | GOBP\_FAT\_SOLUBLE\_VITAMIN\_METABOLIC\_PROCESS |  | 38 | 0.21 | 0.83 | 0.757 | 1.000 | 1.000 | 2372 | tags=26%, list=18%, signal=32% |
| 696 | GOBP\_REGULATION\_OF\_MONOCYTE\_CHEMOTAXIS |  | 24 | 0.25 | 0.83 | 0.734 | 1.000 | 1.000 | 1815 | tags=25%, list=14%, signal=29% |
| 697 | GOBP\_STRIATED\_MUSCLE\_CELL\_DEVELOPMENT |  | 59 | 0.20 | 0.83 | 0.818 | 1.000 | 1.000 | 3490 | tags=41%, list=27%, signal=56% |
| 698 | GOBP\_SEQUESTERING\_OF\_CALCIUM\_ION |  | 108 | 0.18 | 0.83 | 0.852 | 1.000 | 1.000 | 2293 | tags=20%, list=18%, signal=25% |
| 699 | GOBP\_NEGATIVE\_REGULATION\_OF\_ENDOPLASMIC\_RETICULUM\_STRESS\_INDUCED\_INTRINSIC\_APOPTOTIC\_SIGNALING\_PATHWAY |  | 15 | 0.27 | 0.83 | 0.689 | 1.000 | 1.000 | 1972 | tags=20%, list=15%, signal=24% |
| 700 | GOBP\_PROTEOGLYCAN\_METABOLIC\_PROCESS |  | 65 | 0.19 | 0.83 | 0.795 | 1.000 | 1.000 | 3564 | tags=38%, list=28%, signal=53% |
| 701 | GOBP\_REGULATION\_OF\_NITRIC\_OXIDE\_SYNTHASE\_ACTIVITY |  | 35 | 0.22 | 0.83 | 0.748 | 1.000 | 1.000 | 2337 | tags=26%, list=18%, signal=31% |
| 702 | GOBP\_SUPEROXIDE\_METABOLIC\_PROCESS |  | 56 | 0.20 | 0.83 | 0.806 | 1.000 | 1.000 | 2490 | tags=25%, list=19%, signal=31% |
| 703 | GOBP\_B\_CELL\_APOPTOTIC\_PROCESS |  | 20 | 0.25 | 0.83 | 0.706 | 1.000 | 1.000 | 2692 | tags=40%, list=21%, signal=50% |
| 704 | GOBP\_NEGATIVE\_REGULATION\_OF\_ACTIN\_FILAMENT\_BUNDLE\_ASSEMBLY |  | 24 | 0.24 | 0.83 | 0.712 | 1.000 | 1.000 | 1972 | tags=25%, list=15%, signal=29% |
| 705 | GOBP\_MAINTENANCE\_OF\_PROTEIN\_LOCATION\_IN\_NUCLEUS |  | 20 | 0.26 | 0.83 | 0.719 | 1.000 | 1.000 | 1406 | tags=20%, list=11%, signal=22% |
| 706 | GOBP\_REGULATION\_OF\_DNA\_DEPENDENT\_DNA\_REPLICATION |  | 41 | 0.21 | 0.83 | 0.767 | 1.000 | 1.000 | 1825 | tags=22%, list=14%, signal=25% |
| 707 | GOBP\_NEGATIVE\_REGULATION\_OF\_ERBB\_SIGNALING\_PATHWAY |  | 24 | 0.24 | 0.83 | 0.729 | 1.000 | 1.000 | 2715 | tags=33%, list=21%, signal=42% |
| 708 | GOBP\_MUSCLE\_CELL\_CELLULAR\_HOMEOSTASIS |  | 18 | 0.26 | 0.83 | 0.743 | 1.000 | 1.000 | 2413 | tags=33%, list=19%, signal=41% |
| 709 | GOBP\_HETEROTYPIC\_CELL\_CELL\_ADHESION |  | 55 | 0.20 | 0.83 | 0.794 | 1.000 | 1.000 | 2274 | tags=25%, list=18%, signal=31% |
| 710 | GOBP\_TRABECULA\_FORMATION |  | 24 | 0.24 | 0.83 | 0.714 | 1.000 | 1.000 | 2503 | tags=25%, list=19%, signal=31% |
| 711 | GOBP\_MONOSACCHARIDE\_CATABOLIC\_PROCESS |  | 33 | 0.23 | 0.83 | 0.772 | 1.000 | 1.000 | 2580 | tags=27%, list=20%, signal=34% |
| 712 | GOBP\_INTRACELLULAR\_STEROL\_TRANSPORT |  | 21 | 0.25 | 0.82 | 0.760 | 1.000 | 1.000 | 918 | tags=14%, list=7%, signal=15% |
| 713 | GOBP\_REGULATION\_OF\_SYNAPTIC\_VESICLE\_EXOCYTOSIS |  | 46 | 0.21 | 0.82 | 0.775 | 1.000 | 1.000 | 1847 | tags=17%, list=14%, signal=20% |
| 714 | GOBP\_LEUKOCYTE\_MIGRATION\_INVOLVED\_IN\_INFLAMMATORY\_RESPONSE |  | 18 | 0.26 | 0.82 | 0.730 | 1.000 | 1.000 | 2343 | tags=28%, list=18%, signal=34% |
| 715 | GOBP\_REGULATION\_OF\_SYNCYTIUM\_FORMATION\_BY\_PLASMA\_MEMBRANE\_FUSION |  | 25 | 0.24 | 0.82 | 0.738 | 1.000 | 1.000 | 2352 | tags=28%, list=18%, signal=34% |
| 716 | GOBP\_INTERLEUKIN\_1\_PRODUCTION |  | 89 | 0.18 | 0.82 | 0.843 | 1.000 | 1.000 | 2444 | tags=25%, list=19%, signal=30% |
| 717 | GOBP\_REGULATION\_OF\_SPINDLE\_ORGANIZATION |  | 39 | 0.21 | 0.82 | 0.760 | 1.000 | 1.000 | 2783 | tags=23%, list=22%, signal=29% |
| 718 | GOBP\_PURINE\_NUCLEOTIDE\_CATABOLIC\_PROCESS |  | 34 | 0.22 | 0.82 | 0.744 | 1.000 | 1.000 | 1365 | tags=21%, list=11%, signal=23% |
| 719 | GOBP\_VESICLE\_BUDDING\_FROM\_MEMBRANE |  | 55 | 0.20 | 0.82 | 0.810 | 1.000 | 1.000 | 4017 | tags=42%, list=31%, signal=60% |
| 720 | GOBP\_NUCLEOSOME\_ASSEMBLY |  | 88 | 0.18 | 0.82 | 0.838 | 1.000 | 1.000 | 2931 | tags=26%, list=23%, signal=34% |
| 721 | GOBP\_ACTIVATION\_OF\_CYSTEINE\_TYPE\_ENDOPEPTIDASE\_ACTIVITY |  | 16 | 0.27 | 0.82 | 0.734 | 1.000 | 1.000 | 1636 | tags=25%, list=13%, signal=29% |
| 722 | GOBP\_PRODUCTION\_OF\_MOLECULAR\_MEDIATOR\_INVOLVED\_IN\_INFLAMMATORY\_RESPONSE |  | 72 | 0.19 | 0.82 | 0.840 | 1.000 | 1.000 | 2352 | tags=22%, list=18%, signal=27% |
| 723 | GOBP\_MITOCHONDRIAL\_OUTER\_MEMBRANE\_PERMEABILIZATION |  | 27 | 0.24 | 0.82 | 0.732 | 1.000 | 1.000 | 1610 | tags=19%, list=12%, signal=21% |
| 724 | GOBP\_REGULATION\_OF\_VASCULOGENESIS |  | 16 | 0.27 | 0.82 | 0.695 | 1.000 | 1.000 | 505 | tags=13%, list=4%, signal=13% |
| 725 | GOBP\_POSITIVE\_REGULATION\_OF\_ALPHA\_BETA\_T\_CELL\_PROLIFERATION |  | 22 | 0.24 | 0.81 | 0.767 | 1.000 | 1.000 | 2919 | tags=27%, list=23%, signal=35% |
| 726 | GOBP\_REGULATION\_OF\_EARLY\_ENDOSOME\_TO\_LATE\_ENDOSOME\_TRANSPORT |  | 15 | 0.27 | 0.81 | 0.733 | 1.000 | 1.000 | 3817 | tags=40%, list=30%, signal=57% |
| 727 | GOBP\_HOMOLOGOUS\_RECOMBINATION |  | 40 | 0.21 | 0.81 | 0.808 | 1.000 | 1.000 | 1696 | tags=18%, list=13%, signal=20% |
| 728 | GOBP\_RESPONSE\_TO\_ALKALOID |  | 88 | 0.18 | 0.81 | 0.886 | 1.000 | 1.000 | 1847 | tags=17%, list=14%, signal=20% |
| 729 | GOBP\_RESPONSE\_TO\_SALT\_STRESS |  | 26 | 0.24 | 0.81 | 0.768 | 1.000 | 1.000 | 3527 | tags=42%, list=27%, signal=58% |
| 730 | GOBP\_TRIGLYCERIDE\_METABOLIC\_PROCESS |  | 71 | 0.19 | 0.81 | 0.852 | 1.000 | 1.000 | 3042 | tags=27%, list=24%, signal=35% |
| 731 | GOBP\_REGULATION\_OF\_CYTOPLASMIC\_TRANSPORT |  | 24 | 0.24 | 0.81 | 0.741 | 1.000 | 1.000 | 3817 | tags=46%, list=30%, signal=65% |
| 732 | GOBP\_REGULATION\_OF\_CILIUM\_MOVEMENT |  | 21 | 0.25 | 0.81 | 0.731 | 1.000 | 1.000 | 3527 | tags=38%, list=27%, signal=52% |
| 733 | GOBP\_CEREBRAL\_CORTEX\_NEURON\_DIFFERENTIATION |  | 20 | 0.25 | 0.81 | 0.751 | 1.000 | 1.000 | 2278 | tags=25%, list=18%, signal=30% |
| 734 | GOBP\_NEGATIVE\_REGULATION\_OF\_ANTIGEN\_RECEPTOR\_MEDIATED\_SIGNALING\_PATHWAY |  | 28 | 0.23 | 0.81 | 0.773 | 1.000 | 1.000 | 3949 | tags=43%, list=31%, signal=62% |
| 735 | GOBP\_AXONAL\_FASCICULATION |  | 20 | 0.25 | 0.81 | 0.743 | 1.000 | 1.000 | 3704 | tags=55%, list=29%, signal=77% |
| 736 | GOBP\_PHAGOSOME\_MATURATION |  | 19 | 0.25 | 0.81 | 0.751 | 1.000 | 1.000 | 2803 | tags=32%, list=22%, signal=40% |
| 737 | GOBP\_COPII\_COATED\_VESICLE\_BUDDING |  | 28 | 0.23 | 0.81 | 0.763 | 1.000 | 1.000 | 4017 | tags=43%, list=31%, signal=62% |
| 738 | GOBP\_RNA\_METHYLATION |  | 59 | 0.19 | 0.81 | 0.830 | 1.000 | 1.000 | 2181 | tags=19%, list=17%, signal=22% |
| 739 | GOBP\_REGULATION\_OF\_PROTEIN\_EXIT\_FROM\_ENDOPLASMIC\_RETICULUM |  | 18 | 0.26 | 0.81 | 0.723 | 1.000 | 1.000 | 2780 | tags=28%, list=22%, signal=35% |
| 740 | GOBP\_AMYLOID\_BETA\_METABOLIC\_PROCESS |  | 51 | 0.20 | 0.80 | 0.808 | 1.000 | 1.000 | 2776 | tags=27%, list=22%, signal=35% |
| 741 | GOBP\_NEGATIVE\_REGULATION\_OF\_RESPONSE\_TO\_OXIDATIVE\_STRESS |  | 16 | 0.26 | 0.80 | 0.733 | 1.000 | 1.000 | 2547 | tags=38%, list=20%, signal=47% |
| 742 | GOBP\_RETINAL\_GANGLION\_CELL\_AXON\_GUIDANCE |  | 19 | 0.25 | 0.80 | 0.749 | 1.000 | 1.000 | 2373 | tags=32%, list=18%, signal=39% |
| 743 | GOBP\_CELLULAR\_RESPONSE\_TO\_NUTRIENT |  | 39 | 0.21 | 0.80 | 0.812 | 1.000 | 1.000 | 2670 | tags=28%, list=21%, signal=35% |
| 744 | GOBP\_STEROID\_BIOSYNTHETIC\_PROCESS |  | 152 | 0.16 | 0.80 | 0.916 | 1.000 | 1.000 | 2538 | tags=21%, list=20%, signal=26% |
| 745 | GOBP\_NEGATIVE\_REGULATION\_OF\_T\_CELL\_MEDIATED\_IMMUNITY |  | 24 | 0.23 | 0.80 | 0.762 | 1.000 | 1.000 | 3949 | tags=29%, list=31%, signal=42% |
| 746 | GOBP\_REGULATION\_OF\_STEROL\_TRANSPORT |  | 50 | 0.20 | 0.80 | 0.863 | 1.000 | 1.000 | 1855 | tags=18%, list=14%, signal=21% |
| 747 | GOBP\_TRANSCYTOSIS |  | 18 | 0.25 | 0.80 | 0.745 | 1.000 | 1.000 | 2008 | tags=22%, list=16%, signal=26% |
| 748 | GOBP\_NEUROTRANSMITTER\_SECRETION |  | 116 | 0.17 | 0.80 | 0.908 | 1.000 | 1.000 | 1972 | tags=18%, list=15%, signal=21% |
| 749 | GOBP\_REGULATION\_OF\_B\_CELL\_PROLIFERATION |  | 53 | 0.19 | 0.80 | 0.858 | 1.000 | 1.000 | 2692 | tags=30%, list=21%, signal=38% |
| 750 | GOBP\_REGULATION\_OF\_ORGANELLE\_ASSEMBLY |  | 182 | 0.15 | 0.80 | 0.953 | 1.000 | 1.000 | 2140 | tags=18%, list=17%, signal=21% |
| 751 | GOBP\_REGULATION\_OF\_NATURAL\_KILLER\_CELL\_ACTIVATION |  | 30 | 0.22 | 0.80 | 0.783 | 1.000 | 1.000 | 2599 | tags=30%, list=20%, signal=37% |
| 752 | GOBP\_B\_CELL\_PROLIFERATION |  | 82 | 0.18 | 0.80 | 0.905 | 1.000 | 1.000 | 2757 | tags=28%, list=21%, signal=35% |
| 753 | GOBP\_PIGMENT\_GRANULE\_ORGANIZATION |  | 21 | 0.24 | 0.80 | 0.765 | 1.000 | 1.000 | 3569 | tags=48%, list=28%, signal=66% |
| 754 | GOBP\_REGULATION\_OF\_B\_CELL\_MEDIATED\_IMMUNITY |  | 52 | 0.19 | 0.79 | 0.830 | 1.000 | 1.000 | 3434 | tags=33%, list=27%, signal=44% |
| 755 | GOBP\_NEGATIVE\_REGULATION\_OF\_ION\_TRANSMEMBRANE\_TRANSPORTER\_ACTIVITY |  | 52 | 0.19 | 0.79 | 0.840 | 1.000 | 1.000 | 4181 | tags=42%, list=32%, signal=62% |
| 756 | GOBP\_REGULATION\_OF\_MITOCHONDRIAL\_MEMBRANE\_PERMEABILITY |  | 46 | 0.20 | 0.79 | 0.816 | 1.000 | 1.000 | 1610 | tags=15%, list=12%, signal=17% |
| 757 | GOBP\_POSITIVE\_REGULATION\_OF\_LONG\_TERM\_SYNAPTIC\_POTENTIATION |  | 17 | 0.25 | 0.79 | 0.766 | 1.000 | 1.000 | 4475 | tags=41%, list=35%, signal=63% |
| 758 | GOBP\_CHONDROITIN\_SULFATE\_PROTEOGLYCAN\_METABOLIC\_PROCESS |  | 27 | 0.23 | 0.79 | 0.779 | 1.000 | 1.000 | 3564 | tags=37%, list=28%, signal=51% |
| 759 | GOBP\_ORGANELLE\_FISSION |  | 370 | 0.14 | 0.79 | 0.985 | 1.000 | 1.000 | 2535 | tags=20%, list=20%, signal=25% |
| 760 | GOBP\_ASPARTATE\_FAMILY\_AMINO\_ACID\_BIOSYNTHETIC\_PROCESS |  | 19 | 0.25 | 0.79 | 0.758 | 1.000 | 1.000 | 2004 | tags=21%, list=16%, signal=25% |
| 761 | GOBP\_SYNAPSE\_ASSEMBLY |  | 132 | 0.16 | 0.79 | 0.939 | 1.000 | 1.000 | 3400 | tags=27%, list=26%, signal=37% |
| 762 | GOBP\_PROTEIN\_LOCALIZATION\_TO\_GOLGI\_APPARATUS |  | 23 | 0.23 | 0.79 | 0.802 | 1.000 | 1.000 | 1691 | tags=17%, list=13%, signal=20% |
| 763 | GOBP\_ACTIN\_MEDIATED\_CELL\_CONTRACTION |  | 89 | 0.17 | 0.79 | 0.910 | 1.000 | 1.000 | 3161 | tags=26%, list=25%, signal=34% |
| 764 | GOBP\_PROTEIN\_LOCALIZATION\_TO\_CELL\_PERIPHERY |  | 269 | 0.15 | 0.79 | 0.982 | 1.000 | 1.000 | 2592 | tags=22%, list=20%, signal=26% |
| 765 | GOBP\_REGULATION\_OF\_AMYLOID\_BETA\_CLEARANCE |  | 17 | 0.26 | 0.79 | 0.776 | 1.000 | 1.000 | 3047 | tags=35%, list=24%, signal=46% |
| 766 | GOBP\_REGULATION\_OF\_PLASMA\_LIPOPROTEIN\_PARTICLE\_LEVELS |  | 64 | 0.18 | 0.78 | 0.903 | 1.000 | 1.000 | 1855 | tags=19%, list=14%, signal=22% |
| 767 | GOBP\_SPERMATID\_DIFFERENTIATION |  | 108 | 0.17 | 0.78 | 0.922 | 1.000 | 1.000 | 2391 | tags=20%, list=19%, signal=25% |
| 768 | GOBP\_COMPLEMENT\_ACTIVATION\_ALTERNATIVE\_PATHWAY |  | 16 | 0.26 | 0.78 | 0.797 | 1.000 | 1.000 | 4387 | tags=56%, list=34%, signal=85% |
| 769 | GOBP\_REGULATION\_OF\_CD8\_POSITIVE\_ALPHA\_BETA\_T\_CELL\_ACTIVATION |  | 16 | 0.26 | 0.78 | 0.766 | 1.000 | 1.000 | 1089 | tags=19%, list=8%, signal=20% |
| 770 | GOBP\_LYMPHOCYTE\_COSTIMULATION |  | 41 | 0.20 | 0.78 | 0.863 | 1.000 | 1.000 | 3572 | tags=34%, list=28%, signal=47% |
| 771 | GOBP\_NEUTRAL\_LIPID\_METABOLIC\_PROCESS |  | 93 | 0.17 | 0.78 | 0.942 | 1.000 | 1.000 | 2776 | tags=26%, list=22%, signal=33% |
| 772 | GOBP\_CELLULAR\_RESPONSE\_TO\_PROSTAGLANDIN\_STIMULUS |  | 18 | 0.25 | 0.78 | 0.793 | 1.000 | 1.000 | 2503 | tags=33%, list=19%, signal=41% |
| 773 | GOBP\_CELLULAR\_RESPONSE\_TO\_MECHANICAL\_STIMULUS |  | 65 | 0.18 | 0.78 | 0.894 | 1.000 | 1.000 | 924 | tags=11%, list=7%, signal=12% |
| 774 | GOBP\_ALCOHOL\_BIOSYNTHETIC\_PROCESS |  | 123 | 0.16 | 0.78 | 0.944 | 1.000 | 1.000 | 2779 | tags=24%, list=22%, signal=30% |
| 775 | GOBP\_POLYOL\_BIOSYNTHETIC\_PROCESS |  | 53 | 0.19 | 0.77 | 0.865 | 1.000 | 1.000 | 865 | tags=11%, list=7%, signal=12% |
| 776 | GOBP\_NEGATIVE\_REGULATION\_OF\_PHAGOCYTOSIS |  | 18 | 0.24 | 0.77 | 0.809 | 1.000 | 1.000 | 3599 | tags=39%, list=28%, signal=54% |
| 777 | GOBP\_PYRIMIDINE\_NUCLEOBASE\_METABOLIC\_PROCESS |  | 19 | 0.24 | 0.77 | 0.794 | 1.000 | 1.000 | 509 | tags=11%, list=4%, signal=11% |
| 778 | GOBP\_REGULATION\_OF\_ANTIGEN\_RECEPTOR\_MEDIATED\_SIGNALING\_PATHWAY |  | 51 | 0.19 | 0.77 | 0.897 | 1.000 | 1.000 | 3434 | tags=33%, list=27%, signal=45% |
| 779 | GOBP\_PROTEIN\_K48\_LINKED\_UBIQUITINATION |  | 54 | 0.19 | 0.77 | 0.870 | 1.000 | 1.000 | 993 | tags=13%, list=8%, signal=14% |
| 780 | GOBP\_ORGANIC\_HYDROXY\_COMPOUND\_BIOSYNTHETIC\_PROCESS |  | 214 | 0.15 | 0.77 | 0.984 | 1.000 | 1.000 | 2602 | tags=21%, list=20%, signal=25% |
| 781 | GOBP\_POSITIVE\_REGULATION\_OF\_NEUROTRANSMITTER\_SECRETION |  | 15 | 0.27 | 0.77 | 0.778 | 1.000 | 1.000 | 1800 | tags=27%, list=14%, signal=31% |
| 782 | GOBP\_XENOBIOTIC\_METABOLIC\_PROCESS |  | 83 | 0.17 | 0.77 | 0.921 | 1.000 | 1.000 | 1649 | tags=14%, list=13%, signal=16% |
| 783 | GOBP\_INNER\_EAR\_MORPHOGENESIS |  | 76 | 0.17 | 0.77 | 0.921 | 1.000 | 1.000 | 2448 | tags=21%, list=19%, signal=26% |
| 784 | GOBP\_NEGATIVE\_REGULATION\_OF\_UBIQUITIN\_PROTEIN\_TRANSFERASE\_ACTIVITY |  | 19 | 0.25 | 0.77 | 0.804 | 1.000 | 1.000 | 1516 | tags=26%, list=12%, signal=30% |
| 785 | GOBP\_POSITIVE\_REGULATION\_OF\_INSULIN\_SECRETION |  | 58 | 0.19 | 0.77 | 0.885 | 1.000 | 1.000 | 2416 | tags=22%, list=19%, signal=27% |
| 786 | GOBP\_RENAL\_ABSORPTION |  | 19 | 0.24 | 0.77 | 0.820 | 1.000 | 1.000 | 1996 | tags=26%, list=15%, signal=31% |
| 787 | GOBP\_POSITIVE\_REGULATION\_OF\_CD4\_POSITIVE\_ALPHA\_BETA\_T\_CELL\_ACTIVATION |  | 34 | 0.20 | 0.77 | 0.867 | 1.000 | 1.000 | 3789 | tags=38%, list=29%, signal=54% |
| 788 | GOBP\_TRIGLYCERIDE\_CATABOLIC\_PROCESS |  | 22 | 0.23 | 0.77 | 0.800 | 1.000 | 1.000 | 457 | tags=9%, list=4%, signal=9% |
| 789 | GOBP\_RESPONSE\_TO\_PROGESTERONE |  | 36 | 0.21 | 0.77 | 0.834 | 1.000 | 1.000 | 2490 | tags=25%, list=19%, signal=31% |
| 790 | GOBP\_NEGATIVE\_REGULATION\_OF\_ATP\_DEPENDENT\_ACTIVITY |  | 18 | 0.25 | 0.77 | 0.778 | 1.000 | 1.000 | 2510 | tags=28%, list=19%, signal=34% |
| 791 | GOBP\_POSITIVE\_REGULATION\_OF\_PROTEIN\_SECRETION |  | 113 | 0.16 | 0.77 | 0.928 | 1.000 | 1.000 | 2972 | tags=27%, list=23%, signal=34% |
| 792 | GOBP\_MACROPHAGE\_CYTOKINE\_PRODUCTION |  | 17 | 0.25 | 0.77 | 0.791 | 1.000 | 1.000 | 2490 | tags=24%, list=19%, signal=29% |
| 793 | GOBP\_HYPOTHALAMUS\_DEVELOPMENT |  | 19 | 0.24 | 0.76 | 0.823 | 1.000 | 1.000 | 3704 | tags=53%, list=29%, signal=74% |
| 794 | GOBP\_PROTEIN\_LOCALIZATION\_TO\_CONDENSED\_CHROMOSOME |  | 15 | 0.25 | 0.76 | 0.790 | 1.000 | 1.000 | 3744 | tags=40%, list=29%, signal=56% |
| 795 | GOBP\_REGULATION\_OF\_NEUROTRANSMITTER\_LEVELS |  | 179 | 0.15 | 0.76 | 0.959 | 1.000 | 1.000 | 2995 | tags=24%, list=23%, signal=31% |
| 796 | GOBP\_POSITIVE\_REGULATION\_OF\_CD4\_POSITIVE\_ALPHA\_BETA\_T\_CELL\_DIFFERENTIATION |  | 28 | 0.22 | 0.76 | 0.842 | 1.000 | 1.000 | 2760 | tags=29%, list=21%, signal=36% |
| 797 | GOBP\_NATURAL\_KILLER\_CELL\_DIFFERENTIATION |  | 21 | 0.23 | 0.76 | 0.798 | 1.000 | 1.000 | 1942 | tags=24%, list=15%, signal=28% |
| 798 | GOBP\_SECONDARY\_METABOLIC\_PROCESS |  | 51 | 0.19 | 0.76 | 0.875 | 1.000 | 1.000 | 3666 | tags=35%, list=28%, signal=49% |
| 799 | GOBP\_SNRNA\_TRANSCRIPTION |  | 18 | 0.23 | 0.76 | 0.823 | 1.000 | 1.000 | 2753 | tags=28%, list=21%, signal=35% |
| 800 | GOBP\_POSITIVE\_REGULATION\_OF\_SYNAPSE\_ASSEMBLY |  | 44 | 0.19 | 0.76 | 0.898 | 1.000 | 1.000 | 3677 | tags=27%, list=29%, signal=38% |
| 801 | GOBP\_SISTER\_CHROMATID\_SEGREGATION |  | 160 | 0.15 | 0.76 | 0.985 | 1.000 | 1.000 | 3773 | tags=33%, list=29%, signal=45% |
| 802 | GOBP\_RIBONUCLEOSIDE\_CATABOLIC\_PROCESS |  | 16 | 0.25 | 0.76 | 0.833 | 1.000 | 1.000 | 1955 | tags=31%, list=15%, signal=37% |
| 803 | GOBP\_CARDIAC\_MYOFIBRIL\_ASSEMBLY |  | 18 | 0.24 | 0.76 | 0.803 | 1.000 | 1.000 | 3490 | tags=39%, list=27%, signal=53% |
| 804 | GOBP\_SIGNALING\_RECEPTOR\_LIGAND\_PRECURSOR\_PROCESSING |  | 31 | 0.21 | 0.76 | 0.844 | 1.000 | 1.000 | 2457 | tags=26%, list=19%, signal=32% |
| 805 | GOBP\_CATECHOLAMINE\_UPTAKE |  | 16 | 0.25 | 0.76 | 0.825 | 1.000 | 1.000 | 1972 | tags=25%, list=15%, signal=29% |
| 806 | GOBP\_MEIOSIS\_I\_CELL\_CYCLE\_PROCESS |  | 88 | 0.16 | 0.75 | 0.949 | 1.000 | 1.000 | 3184 | tags=25%, list=25%, signal=33% |
| 807 | GOBP\_CALCIUM\_MEDIATED\_SIGNALING\_USING\_INTRACELLULAR\_CALCIUM\_SOURCE |  | 22 | 0.22 | 0.75 | 0.849 | 1.000 | 1.000 | 2966 | tags=32%, list=23%, signal=41% |
| 808 | GOBP\_REGULATION\_OF\_MORPHOGENESIS\_OF\_A\_BRANCHING\_STRUCTURE |  | 45 | 0.19 | 0.75 | 0.866 | 1.000 | 1.000 | 2700 | tags=29%, list=21%, signal=36% |
| 809 | GOBP\_NEGATIVE\_REGULATION\_OF\_INTERLEUKIN\_6\_PRODUCTION |  | 40 | 0.20 | 0.75 | 0.867 | 1.000 | 1.000 | 3600 | tags=40%, list=28%, signal=55% |
| 810 | GOBP\_RETROGRADE\_AXONAL\_TRANSPORT |  | 17 | 0.24 | 0.75 | 0.820 | 1.000 | 1.000 | 2877 | tags=41%, list=22%, signal=53% |
| 811 | GOBP\_NEGATIVE\_REGULATION\_OF\_POSTTRANSCRIPTIONAL\_GENE\_SILENCING |  | 16 | 0.25 | 0.75 | 0.812 | 1.000 | 1.000 | 2549 | tags=31%, list=20%, signal=39% |
| 812 | GOBP\_CARDIAC\_MUSCLE\_TISSUE\_MORPHOGENESIS |  | 53 | 0.18 | 0.75 | 0.906 | 1.000 | 1.000 | 4299 | tags=49%, list=33%, signal=73% |
| 813 | GOBP\_MUSCLE\_ORGAN\_MORPHOGENESIS |  | 70 | 0.17 | 0.75 | 0.940 | 1.000 | 1.000 | 2816 | tags=27%, list=22%, signal=35% |
| 814 | GOBP\_NEGATIVE\_REGULATION\_OF\_VIRAL\_GENOME\_REPLICATION |  | 49 | 0.19 | 0.75 | 0.884 | 1.000 | 1.000 | 3006 | tags=33%, list=23%, signal=42% |
| 815 | GOBP\_RESPONSE\_TO\_MITOCHONDRIAL\_DEPOLARISATION |  | 19 | 0.23 | 0.75 | 0.841 | 1.000 | 1.000 | 3810 | tags=42%, list=30%, signal=60% |
| 816 | GOBP\_REGULATION\_OF\_PROTEIN\_LOCALIZATION\_TO\_CELL\_SURFACE |  | 36 | 0.20 | 0.75 | 0.888 | 1.000 | 1.000 | 2877 | tags=31%, list=22%, signal=39% |
| 817 | GOBP\_NEUROMUSCULAR\_PROCESS\_CONTROLLING\_BALANCE |  | 35 | 0.20 | 0.75 | 0.884 | 1.000 | 1.000 | 2684 | tags=23%, list=21%, signal=29% |
| 818 | GOBP\_CELL\_CELL\_SIGNALING\_INVOLVED\_IN\_CARDIAC\_CONDUCTION |  | 27 | 0.22 | 0.74 | 0.851 | 1.000 | 1.000 | 828 | tags=11%, list=6%, signal=12% |
| 819 | GOBP\_INNATE\_IMMUNE\_RESPONSE\_ACTIVATING\_SIGNAL\_TRANSDUCTION |  | 24 | 0.22 | 0.74 | 0.863 | 1.000 | 1.000 | 4084 | tags=42%, list=32%, signal=61% |
| 820 | GOBP\_REVERSE\_CHOLESTEROL\_TRANSPORT |  | 17 | 0.24 | 0.74 | 0.829 | 1.000 | 1.000 | 2083 | tags=29%, list=16%, signal=35% |
| 821 | GOBP\_PATHWAY\_RESTRICTED\_SMAD\_PROTEIN\_PHOSPHORYLATION |  | 60 | 0.18 | 0.74 | 0.942 | 1.000 | 1.000 | 2490 | tags=18%, list=19%, signal=23% |
| 822 | GOBP\_REGULATION\_OF\_NEUROTRANSMITTER\_UPTAKE |  | 17 | 0.24 | 0.74 | 0.818 | 1.000 | 1.000 | 1972 | tags=24%, list=15%, signal=28% |
| 823 | GOBP\_POSITIVE\_REGULATION\_OF\_HETEROTYPIC\_CELL\_CELL\_ADHESION |  | 15 | 0.25 | 0.74 | 0.826 | 1.000 | 1.000 | 2274 | tags=27%, list=18%, signal=32% |
| 824 | GOBP\_MEMBRANE\_DOCKING |  | 69 | 0.17 | 0.74 | 0.936 | 1.000 | 1.000 | 676 | tags=9%, list=5%, signal=9% |
| 825 | GOBP\_DNA\_STRAND\_ELONGATION\_INVOLVED\_IN\_DNA\_REPLICATION |  | 15 | 0.25 | 0.74 | 0.793 | 1.000 | 1.000 | 2898 | tags=33%, list=22%, signal=43% |
| 826 | GOBP\_POSITIVE\_REGULATION\_OF\_PROTEIN\_LOCALIZATION\_TO\_CELL\_SURFACE |  | 17 | 0.24 | 0.74 | 0.839 | 1.000 | 1.000 | 2098 | tags=29%, list=16%, signal=35% |
| 827 | GOBP\_REGULATION\_OF\_CARDIAC\_MUSCLE\_CONTRACTION\_BY\_REGULATION\_OF\_THE\_RELEASE\_OF\_SEQUESTERED\_CALCIUM\_ION |  | 17 | 0.24 | 0.74 | 0.844 | 1.000 | 1.000 | 1214 | tags=18%, list=9%, signal=19% |
| 828 | GOBP\_OLIGOSACCHARIDE\_BIOSYNTHETIC\_PROCESS |  | 24 | 0.22 | 0.74 | 0.841 | 1.000 | 1.000 | 2883 | tags=33%, list=22%, signal=43% |
| 829 | GOBP\_RESPONSE\_TO\_PROSTAGLANDIN |  | 28 | 0.21 | 0.74 | 0.873 | 1.000 | 1.000 | 2503 | tags=29%, list=19%, signal=35% |
| 830 | GOBP\_SKELETAL\_MUSCLE\_TISSUE\_REGENERATION |  | 29 | 0.21 | 0.74 | 0.871 | 1.000 | 1.000 | 804 | tags=14%, list=6%, signal=15% |
| 831 | GOBP\_POSITIVE\_REGULATION\_OF\_INTERLEUKIN\_12\_PRODUCTION |  | 38 | 0.19 | 0.74 | 0.892 | 1.000 | 1.000 | 725 | tags=8%, list=6%, signal=8% |
| 832 | GOBP\_VESICLE\_DOCKING\_INVOLVED\_IN\_EXOCYTOSIS |  | 28 | 0.21 | 0.74 | 0.885 | 1.000 | 1.000 | 650 | tags=11%, list=5%, signal=11% |
| 833 | GOBP\_NEGATIVE\_REGULATION\_OF\_DNA\_TEMPLATED\_TRANSCRIPTION\_ELONGATION |  | 17 | 0.24 | 0.74 | 0.848 | 1.000 | 1.000 | 1135 | tags=18%, list=9%, signal=19% |
| 834 | GOBP\_RELEASE\_OF\_SEQUESTERED\_CALCIUM\_ION\_INTO\_CYTOSOL\_BY\_ENDOPLASMIC\_RETICULUM |  | 28 | 0.21 | 0.73 | 0.899 | 1.000 | 1.000 | 3931 | tags=46%, list=30%, signal=67% |
| 835 | GOBP\_ANTIBACTERIAL\_HUMORAL\_RESPONSE |  | 47 | 0.18 | 0.73 | 0.911 | 1.000 | 1.000 | 3377 | tags=38%, list=26%, signal=52% |
| 836 | GOBP\_SULFATION |  | 19 | 0.23 | 0.73 | 0.840 | 1.000 | 1.000 | 3321 | tags=42%, list=26%, signal=57% |
| 837 | GOBP\_BONE\_GROWTH |  | 28 | 0.21 | 0.73 | 0.876 | 1.000 | 1.000 | 451 | tags=11%, list=3%, signal=11% |
| 838 | GOBP\_NEGATIVE\_REGULATION\_OF\_AMYLOID\_PRECURSOR\_PROTEIN\_CATABOLIC\_PROCESS |  | 21 | 0.22 | 0.73 | 0.867 | 1.000 | 1.000 | 2776 | tags=33%, list=22%, signal=42% |
| 839 | GOBP\_SYNAPTIC\_VESICLE\_EXOCYTOSIS |  | 82 | 0.16 | 0.73 | 0.962 | 1.000 | 1.000 | 1847 | tags=16%, list=14%, signal=18% |
| 840 | GOBP\_MITOCHONDRIAL\_RNA\_PROCESSING |  | 16 | 0.24 | 0.73 | 0.860 | 1.000 | 1.000 | 2473 | tags=31%, list=19%, signal=39% |
| 841 | GOBP\_XENOBIOTIC\_CATABOLIC\_PROCESS |  | 22 | 0.22 | 0.73 | 0.862 | 1.000 | 1.000 | 1025 | tags=14%, list=8%, signal=15% |
| 842 | GOBP\_MEMBRANE\_BIOGENESIS |  | 53 | 0.18 | 0.72 | 0.914 | 1.000 | 1.000 | 1502 | tags=17%, list=12%, signal=19% |
| 843 | GOBP\_REGULATION\_OF\_RELEASE\_OF\_SEQUESTERED\_CALCIUM\_ION\_INTO\_CYTOSOL |  | 64 | 0.17 | 0.72 | 0.940 | 1.000 | 1.000 | 3789 | tags=38%, list=29%, signal=53% |
| 844 | GOBP\_LEUKOCYTE\_DEGRANULATION |  | 72 | 0.16 | 0.72 | 0.958 | 1.000 | 1.000 | 2692 | tags=25%, list=21%, signal=31% |
| 845 | GOBP\_REGULATION\_OF\_ENDOPLASMIC\_RETICULUM\_STRESS\_INDUCED\_INTRINSIC\_APOPTOTIC\_SIGNALING\_PATHWAY |  | 25 | 0.21 | 0.72 | 0.884 | 1.000 | 1.000 | 1972 | tags=20%, list=15%, signal=24% |
| 846 | GOBP\_VITAMIN\_D\_METABOLIC\_PROCESS |  | 18 | 0.23 | 0.72 | 0.847 | 1.000 | 1.000 | 2098 | tags=22%, list=16%, signal=27% |
| 847 | GOBP\_CELL\_COMMUNICATION\_BY\_ELECTRICAL\_COUPLING\_INVOLVED\_IN\_CARDIAC\_CONDUCTION |  | 23 | 0.22 | 0.72 | 0.873 | 1.000 | 1.000 | 1177 | tags=17%, list=9%, signal=19% |
| 848 | GOBP\_ESTABLISHMENT\_OF\_PROTEIN\_LOCALIZATION\_TO\_VACUOLE |  | 38 | 0.19 | 0.72 | 0.931 | 1.000 | 1.000 | 3074 | tags=34%, list=24%, signal=45% |
| 849 | GOBP\_POSITIVE\_REGULATION\_OF\_CELL\_ADHESION\_MEDIATED\_BY\_INTEGRIN |  | 19 | 0.23 | 0.72 | 0.859 | 1.000 | 1.000 | 4171 | tags=53%, list=32%, signal=78% |
| 850 | GOBP\_POSITIVE\_REGULATION\_OF\_PATHWAY\_RESTRICTED\_SMAD\_PROTEIN\_PHOSPHORYLATION |  | 47 | 0.18 | 0.72 | 0.931 | 1.000 | 1.000 | 928 | tags=9%, list=7%, signal=9% |
| 851 | GOBP\_BLASTOCYST\_GROWTH |  | 15 | 0.24 | 0.72 | 0.848 | 1.000 | 1.000 | 4494 | tags=53%, list=35%, signal=82% |
| 852 | GOBP\_VESICLE\_DOCKING |  | 46 | 0.18 | 0.72 | 0.933 | 1.000 | 1.000 | 650 | tags=9%, list=5%, signal=9% |
| 853 | GOBP\_RESPONSE\_TO\_FOOD |  | 30 | 0.20 | 0.71 | 0.902 | 1.000 | 1.000 | 2365 | tags=20%, list=18%, signal=24% |
| 854 | GOBP\_FATTY\_ACID\_DERIVATIVE\_METABOLIC\_PROCESS |  | 51 | 0.17 | 0.71 | 0.952 | 1.000 | 1.000 | 2017 | tags=20%, list=16%, signal=23% |
| 855 | GOBP\_TOLL\_LIKE\_RECEPTOR\_2\_SIGNALING\_PATHWAY |  | 15 | 0.24 | 0.71 | 0.868 | 1.000 | 1.000 | 501 | tags=13%, list=4%, signal=14% |
| 856 | GOBP\_NEURONAL\_ACTION\_POTENTIAL |  | 34 | 0.19 | 0.71 | 0.913 | 1.000 | 1.000 | 1827 | tags=18%, list=14%, signal=21% |
| 857 | GOBP\_CAMERA\_TYPE\_EYE\_PHOTORECEPTOR\_CELL\_DIFFERENTIATION |  | 19 | 0.23 | 0.71 | 0.850 | 1.000 | 1.000 | 3705 | tags=37%, list=29%, signal=52% |
| 858 | GOBP\_NEGATIVE\_REGULATION\_OF\_TRANSPORTER\_ACTIVITY |  | 58 | 0.17 | 0.71 | 0.947 | 1.000 | 1.000 | 4181 | tags=41%, list=32%, signal=61% |
| 859 | GOBP\_REGULATION\_OF\_CARDIAC\_MUSCLE\_CELL\_MEMBRANE\_REPOLARIZATION |  | 21 | 0.22 | 0.71 | 0.874 | 1.000 | 1.000 | 451 | tags=10%, list=3%, signal=10% |
| 860 | GOBP\_POSITIVE\_REGULATION\_OF\_VIRAL\_LIFE\_CYCLE |  | 19 | 0.23 | 0.71 | 0.899 | 1.000 | 1.000 | 777 | tags=11%, list=6%, signal=11% |
| 861 | GOBP\_REGULATION\_OF\_ICOSANOID\_SECRETION |  | 22 | 0.21 | 0.71 | 0.871 | 1.000 | 1.000 | 2656 | tags=32%, list=21%, signal=40% |
| 862 | GOBP\_VESICLE\_TARGETING\_TO\_FROM\_OR\_WITHIN\_GOLGI |  | 19 | 0.22 | 0.71 | 0.870 | 1.000 | 1.000 | 3719 | tags=42%, list=29%, signal=59% |
| 863 | GOBP\_GANGLION\_DEVELOPMENT |  | 15 | 0.24 | 0.71 | 0.864 | 1.000 | 1.000 | 2531 | tags=33%, list=20%, signal=41% |
| 864 | GOBP\_VISUAL\_BEHAVIOR |  | 39 | 0.18 | 0.71 | 0.926 | 1.000 | 1.000 | 3403 | tags=33%, list=26%, signal=45% |
| 865 | GOBP\_NEURON\_FATE\_SPECIFICATION |  | 21 | 0.21 | 0.71 | 0.878 | 1.000 | 1.000 | 2861 | tags=29%, list=22%, signal=37% |
| 866 | GOBP\_DNA\_REPLICATION\_DEPENDENT\_CHROMATIN\_ORGANIZATION |  | 26 | 0.20 | 0.71 | 0.881 | 1.000 | 1.000 | 3221 | tags=31%, list=25%, signal=41% |
| 867 | GOBP\_NUCLEAR\_CHROMOSOME\_SEGREGATION |  | 207 | 0.14 | 0.71 | 1.000 | 1.000 | 1.000 | 2492 | tags=19%, list=19%, signal=23% |
| 868 | GOBP\_UBIQUITIN\_DEPENDENT\_ERAD\_PATHWAY |  | 61 | 0.17 | 0.70 | 0.949 | 1.000 | 1.000 | 2587 | tags=21%, list=20%, signal=27% |
| 869 | GOBP\_LIPID\_DROPLET\_ORGANIZATION |  | 17 | 0.23 | 0.70 | 0.860 | 1.000 | 1.000 | 46 | tags=6%, list=0%, signal=6% |
| 870 | GOBP\_POSITIVE\_REGULATION\_OF\_CHOLESTEROL\_EFFLUX |  | 24 | 0.20 | 0.70 | 0.917 | 1.000 | 1.000 | 1728 | tags=17%, list=13%, signal=19% |
| 871 | GOBP\_NEURAL\_TUBE\_PATTERNING |  | 26 | 0.21 | 0.70 | 0.895 | 1.000 | 1.000 | 1251 | tags=15%, list=10%, signal=17% |
| 872 | GOBP\_DORSAL\_VENTRAL\_NEURAL\_TUBE\_PATTERNING |  | 16 | 0.23 | 0.70 | 0.890 | 1.000 | 1.000 | 771 | tags=13%, list=6%, signal=13% |
| 873 | GOBP\_REGULATION\_OF\_INSULIN\_LIKE\_GROWTH\_FACTOR\_RECEPTOR\_SIGNALING\_PATHWAY |  | 19 | 0.22 | 0.69 | 0.898 | 1.000 | 1.000 | 2721 | tags=32%, list=21%, signal=40% |
| 874 | GOBP\_NUCLEOSIDE\_MONOPHOSPHATE\_CATABOLIC\_PROCESS |  | 15 | 0.23 | 0.69 | 0.878 | 1.000 | 1.000 | 719 | tags=13%, list=6%, signal=14% |
| 875 | GOBP\_BODY\_FLUID\_SECRETION |  | 78 | 0.16 | 0.69 | 0.962 | 1.000 | 1.000 | 3043 | tags=24%, list=24%, signal=32% |
| 876 | GOBP\_GOLGI\_TO\_PLASMA\_MEMBRANE\_TRANSPORT |  | 48 | 0.17 | 0.69 | 0.970 | 1.000 | 1.000 | 2370 | tags=19%, list=18%, signal=23% |
| 877 | GOBP\_CELLULAR\_RESPONSE\_TO\_ALKALOID |  | 31 | 0.19 | 0.69 | 0.909 | 1.000 | 1.000 | 3202 | tags=29%, list=25%, signal=39% |
| 878 | GOBP\_POSITIVE\_REGULATION\_OF\_HUMORAL\_IMMUNE\_RESPONSE |  | 19 | 0.22 | 0.69 | 0.874 | 1.000 | 1.000 | 3154 | tags=32%, list=24%, signal=42% |
| 879 | GOBP\_FATTY\_ACYL\_COA\_BIOSYNTHETIC\_PROCESS |  | 21 | 0.22 | 0.69 | 0.896 | 1.000 | 1.000 | 2520 | tags=24%, list=20%, signal=30% |
| 880 | GOBP\_MAINTENANCE\_OF\_CELL\_POLARITY |  | 16 | 0.23 | 0.69 | 0.882 | 1.000 | 1.000 | 4221 | tags=44%, list=33%, signal=65% |
| 881 | GOBP\_REGULATION\_OF\_LIPOPROTEIN\_LIPASE\_ACTIVITY |  | 16 | 0.23 | 0.69 | 0.886 | 1.000 | 1.000 | 1855 | tags=25%, list=14%, signal=29% |
| 882 | GOBP\_SPLICEOSOMAL\_SNRNP\_ASSEMBLY |  | 33 | 0.19 | 0.69 | 0.941 | 1.000 | 1.000 | 1671 | tags=18%, list=13%, signal=21% |
| 883 | GOBP\_NEGATIVE\_REGULATION\_OF\_LEUKOCYTE\_CHEMOTAXIS |  | 20 | 0.21 | 0.69 | 0.874 | 1.000 | 1.000 | 1646 | tags=20%, list=13%, signal=23% |
| 884 | GOBP\_REGULATION\_OF\_SYNAPSE\_ASSEMBLY |  | 74 | 0.15 | 0.69 | 0.969 | 1.000 | 1.000 | 2483 | tags=20%, list=19%, signal=25% |
| 885 | GOBP\_REGULATION\_OF\_MEGAKARYOCYTE\_DIFFERENTIATION |  | 27 | 0.19 | 0.69 | 0.917 | 1.000 | 1.000 | 872 | tags=11%, list=7%, signal=12% |
| 886 | GOBP\_AXIS\_ELONGATION |  | 21 | 0.21 | 0.68 | 0.913 | 1.000 | 1.000 | 2588 | tags=33%, list=20%, signal=42% |
| 887 | GOBP\_ARACHIDONIC\_ACID\_SECRETION |  | 24 | 0.20 | 0.68 | 0.903 | 1.000 | 1.000 | 1597 | tags=17%, list=12%, signal=19% |
| 888 | GOBP\_ENDOPLASMIC\_RETICULUM\_TO\_CYTOSOL\_TRANSPORT |  | 19 | 0.21 | 0.68 | 0.890 | 1.000 | 1.000 | 3855 | tags=32%, list=30%, signal=45% |
| 889 | GOBP\_NEGATIVE\_REGULATION\_OF\_ION\_TRANSMEMBRANE\_TRANSPORT |  | 68 | 0.16 | 0.68 | 0.972 | 1.000 | 1.000 | 4181 | tags=43%, list=32%, signal=63% |
| 890 | GOBP\_ERAD\_PATHWAY |  | 78 | 0.15 | 0.68 | 0.980 | 1.000 | 1.000 | 2367 | tags=19%, list=18%, signal=23% |
| 891 | GOBP\_GANGLIOSIDE\_METABOLIC\_PROCESS |  | 21 | 0.20 | 0.68 | 0.896 | 1.000 | 1.000 | 3678 | tags=43%, list=29%, signal=60% |
| 892 | GOBP\_REGULATION\_OF\_COLLATERAL\_SPROUTING |  | 18 | 0.22 | 0.68 | 0.885 | 1.000 | 1.000 | 3381 | tags=33%, list=26%, signal=45% |
| 893 | GOBP\_REGULATION\_OF\_CALCIUM\_ION\_TRANSMEMBRANE\_TRANSPORTER\_ACTIVITY |  | 70 | 0.16 | 0.68 | 0.971 | 1.000 | 1.000 | 3400 | tags=30%, list=26%, signal=41% |
| 894 | GOBP\_ACTION\_POTENTIAL |  | 124 | 0.14 | 0.67 | 0.994 | 1.000 | 1.000 | 2187 | tags=16%, list=17%, signal=19% |
| 895 | GOBP\_RESPONSE\_TO\_INTERFERON\_ALPHA |  | 20 | 0.21 | 0.67 | 0.918 | 1.000 | 1.000 | 2115 | tags=25%, list=16%, signal=30% |
| 896 | GOBP\_RESPONSE\_TO\_DEXAMETHASONE |  | 36 | 0.18 | 0.67 | 0.947 | 1.000 | 1.000 | 2466 | tags=28%, list=19%, signal=34% |
| 897 | GOBP\_PROTEIN\_TARGETING\_TO\_VACUOLE |  | 31 | 0.19 | 0.67 | 0.951 | 1.000 | 1.000 | 3074 | tags=32%, list=24%, signal=42% |
| 898 | GOBP\_DOPAMINE\_TRANSPORT |  | 40 | 0.17 | 0.67 | 0.962 | 1.000 | 1.000 | 2097 | tags=20%, list=16%, signal=24% |
| 899 | GOBP\_REGULATION\_OF\_DOUBLE\_STRAND\_BREAK\_REPAIR\_VIA\_NONHOMOLOGOUS\_END\_JOINING |  | 23 | 0.20 | 0.67 | 0.941 | 1.000 | 1.000 | 1662 | tags=17%, list=13%, signal=20% |
| 900 | GOBP\_ESTABLISHMENT\_OF\_PROTEIN\_LOCALIZATION\_TO\_TELOMERE |  | 16 | 0.22 | 0.66 | 0.903 | 1.000 | 1.000 | 3853 | tags=44%, list=30%, signal=62% |
| 901 | GOBP\_NEUTROPHIL\_ACTIVATION\_INVOLVED\_IN\_IMMUNE\_RESPONSE |  | 20 | 0.20 | 0.66 | 0.931 | 1.000 | 1.000 | 2752 | tags=25%, list=21%, signal=32% |
| 902 | GOBP\_PROTEIN\_LOCALIZATION\_TO\_CHROMOSOME |  | 77 | 0.15 | 0.66 | 0.987 | 1.000 | 1.000 | 3853 | tags=38%, list=30%, signal=53% |
| 903 | GOBP\_SINGLE\_FERTILIZATION |  | 102 | 0.14 | 0.66 | 1.000 | 1.000 | 1.000 | 3890 | tags=32%, list=30%, signal=46% |
| 904 | GOBP\_T\_CELL\_RECEPTOR\_SIGNALING\_PATHWAY |  | 111 | 0.14 | 0.66 | 0.997 | 1.000 | 1.000 | 3158 | tags=25%, list=24%, signal=33% |
| 905 | GOBP\_REGULATION\_OF\_T\_CELL\_CHEMOTAXIS |  | 15 | 0.22 | 0.66 | 0.924 | 1.000 | 1.000 | 1995 | tags=27%, list=15%, signal=32% |
| 906 | GOBP\_ACTIVATION\_OF\_NF\_KAPPAB\_INDUCING\_KINASE\_ACTIVITY |  | 15 | 0.23 | 0.66 | 0.906 | 1.000 | 1.000 | 1701 | tags=20%, list=13%, signal=23% |
| 907 | GOBP\_SULFUR\_AMINO\_ACID\_METABOLIC\_PROCESS |  | 31 | 0.18 | 0.66 | 0.959 | 1.000 | 1.000 | 3362 | tags=29%, list=26%, signal=39% |
| 908 | GOBP\_MEIOTIC\_CHROMOSOME\_SEGREGATION |  | 61 | 0.16 | 0.66 | 0.973 | 1.000 | 1.000 | 3574 | tags=30%, list=28%, signal=41% |
| 909 | GOBP\_REGULATION\_OF\_RESPONSE\_TO\_EXTRACELLULAR\_STIMULUS |  | 22 | 0.20 | 0.66 | 0.930 | 1.000 | 1.000 | 366 | tags=9%, list=3%, signal=9% |
| 910 | GOBP\_BINDING\_OF\_SPERM\_TO\_ZONA\_PELLUCIDA |  | 27 | 0.19 | 0.66 | 0.923 | 1.000 | 1.000 | 1139 | tags=11%, list=9%, signal=12% |
| 911 | GOBP\_POSITIVE\_REGULATION\_OF\_RELEASE\_OF\_SEQUESTERED\_CALCIUM\_ION\_INTO\_CYTOSOL |  | 36 | 0.18 | 0.66 | 0.949 | 1.000 | 1.000 | 1800 | tags=19%, list=14%, signal=23% |
| 912 | GOBP\_REGULATION\_OF\_ACTIN\_FILAMENT\_BASED\_MOVEMENT |  | 37 | 0.17 | 0.66 | 0.969 | 1.000 | 1.000 | 1029 | tags=11%, list=8%, signal=12% |
| 913 | GOBP\_REGULATION\_OF\_PRESYNAPSE\_ORGANIZATION |  | 22 | 0.20 | 0.66 | 0.940 | 1.000 | 1.000 | 2483 | tags=27%, list=19%, signal=34% |
| 914 | GOBP\_NEGATIVE\_REGULATION\_OF\_NUCLEOCYTOPLASMIC\_TRANSPORT |  | 21 | 0.20 | 0.66 | 0.945 | 1.000 | 1.000 | 3687 | tags=43%, list=29%, signal=60% |
| 915 | GOBP\_LONG\_CHAIN\_FATTY\_ACYL\_COA\_BIOSYNTHETIC\_PROCESS |  | 16 | 0.22 | 0.66 | 0.899 | 1.000 | 1.000 | 2520 | tags=25%, list=20%, signal=31% |
| 916 | GOBP\_CELLULAR\_RESPONSE\_TO\_LOW\_DENSITY\_LIPOPROTEIN\_PARTICLE\_STIMULUS |  | 17 | 0.22 | 0.65 | 0.917 | 1.000 | 1.000 | 1752 | tags=24%, list=14%, signal=27% |
| 917 | GOBP\_ANION\_HOMEOSTASIS |  | 35 | 0.18 | 0.65 | 0.966 | 1.000 | 1.000 | 2868 | tags=26%, list=22%, signal=33% |
| 918 | GOBP\_REGULATION\_OF\_MULTICELLULAR\_ORGANISM\_GROWTH |  | 58 | 0.16 | 0.65 | 0.976 | 1.000 | 1.000 | 3462 | tags=33%, list=27%, signal=45% |
| 919 | GOBP\_ASTROCYTE\_DEVELOPMENT |  | 37 | 0.17 | 0.65 | 0.956 | 1.000 | 1.000 | 2962 | tags=30%, list=23%, signal=38% |
| 920 | GOBP\_REGULATION\_OF\_SYSTEMIC\_ARTERIAL\_BLOOD\_PRESSURE\_BY\_RENIN\_ANGIOTENSIN |  | 23 | 0.19 | 0.65 | 0.951 | 1.000 | 1.000 | 1259 | tags=13%, list=10%, signal=14% |
| 921 | GOBP\_MOTILE\_CILIUM\_ASSEMBLY |  | 30 | 0.17 | 0.65 | 0.955 | 1.000 | 1.000 | 4190 | tags=40%, list=32%, signal=59% |
| 922 | GOBP\_POSITIVE\_REGULATION\_OF\_OLIGODENDROCYTE\_DIFFERENTIATION |  | 18 | 0.20 | 0.65 | 0.946 | 1.000 | 1.000 | 1894 | tags=17%, list=15%, signal=20% |
| 923 | GOBP\_ATRIAL\_CARDIAC\_MUSCLE\_CELL\_TO\_AV\_NODE\_CELL\_SIGNALING |  | 17 | 0.21 | 0.64 | 0.934 | 1.000 | 1.000 | 828 | tags=12%, list=6%, signal=13% |
| 924 | GOBP\_INFLAMMATORY\_CELL\_APOPTOTIC\_PROCESS |  | 20 | 0.20 | 0.64 | 0.946 | 1.000 | 1.000 | 3502 | tags=40%, list=27%, signal=55% |
| 925 | GOBP\_GROWTH\_PLATE\_CARTILAGE\_DEVELOPMENT |  | 17 | 0.21 | 0.64 | 0.936 | 1.000 | 1.000 | 451 | tags=12%, list=3%, signal=12% |
| 926 | GOBP\_POSITIVE\_REGULATION\_OF\_NEUROTRANSMITTER\_TRANSPORT |  | 20 | 0.20 | 0.64 | 0.952 | 1.000 | 1.000 | 1972 | tags=25%, list=15%, signal=29% |
| 927 | GOBP\_VESICLE\_MEDIATED\_TRANSPORT\_TO\_THE\_PLASMA\_MEMBRANE |  | 109 | 0.14 | 0.64 | 0.997 | 1.000 | 1.000 | 2601 | tags=18%, list=20%, signal=23% |
| 928 | GOBP\_SENSORY\_PERCEPTION\_OF\_PAIN |  | 90 | 0.14 | 0.64 | 0.997 | 1.000 | 1.000 | 1847 | tags=14%, list=14%, signal=17% |
| 929 | GOBP\_POLYAMINE\_METABOLIC\_PROCESS |  | 16 | 0.21 | 0.64 | 0.949 | 1.000 | 1.000 | 848 | tags=13%, list=7%, signal=13% |
| 930 | GOBP\_CYTOKINE\_PRODUCTION\_INVOLVED\_IN\_INFLAMMATORY\_RESPONSE |  | 47 | 0.16 | 0.63 | 0.989 | 1.000 | 1.000 | 3776 | tags=38%, list=29%, signal=54% |
| 931 | GOBP\_REGULATION\_OF\_TRANSPOSITION |  | 19 | 0.20 | 0.63 | 0.971 | 1.000 | 1.000 | 1917 | tags=21%, list=15%, signal=25% |
| 932 | GOBP\_AMINO\_ACID\_BETAINE\_METABOLIC\_PROCESS |  | 15 | 0.21 | 0.63 | 0.920 | 1.000 | 1.000 | 10179 | tags=100%, list=79%, signal=473% |
| 933 | GOBP\_ENDOCYTIC\_RECYCLING |  | 61 | 0.15 | 0.63 | 0.992 | 1.000 | 1.000 | 3745 | tags=31%, list=29%, signal=44% |
| 934 | GOBP\_REGULATION\_OF\_VIRAL\_TRANSCRIPTION |  | 18 | 0.21 | 0.63 | 0.938 | 1.000 | 1.000 | 2311 | tags=28%, list=18%, signal=34% |
| 935 | GOBP\_STRIATED\_MUSCLE\_CONTRACTION |  | 150 | 0.13 | 0.63 | 1.000 | 1.000 | 1.000 | 3161 | tags=25%, list=25%, signal=32% |
| 936 | GOBP\_CHONDROITIN\_SULFATE\_PROTEOGLYCAN\_BIOSYNTHETIC\_PROCESS |  | 17 | 0.21 | 0.63 | 0.941 | 1.000 | 1.000 | 2987 | tags=29%, list=23%, signal=38% |
| 937 | GOBP\_PROTEIN\_LOCALIZATION\_TO\_MICROTUBULE |  | 15 | 0.21 | 0.62 | 0.935 | 1.000 | 1.000 | 3242 | tags=33%, list=25%, signal=44% |
| 938 | GOBP\_ESTABLISHMENT\_OF\_PROTEIN\_LOCALIZATION\_TO\_CHROMOSOME |  | 24 | 0.18 | 0.62 | 0.966 | 1.000 | 1.000 | 3853 | tags=46%, list=30%, signal=65% |
| 939 | GOBP\_FOREBRAIN\_NEURON\_DEVELOPMENT |  | 19 | 0.20 | 0.62 | 0.949 | 1.000 | 1.000 | 3104 | tags=32%, list=24%, signal=42% |
| 940 | GOBP\_REGULATION\_OF\_HEART\_RATE\_BY\_CARDIAC\_CONDUCTION |  | 33 | 0.17 | 0.62 | 0.974 | 1.000 | 1.000 | 2387 | tags=18%, list=19%, signal=22% |
| 941 | GOBP\_NEGATIVE\_REGULATION\_OF\_CALCIUM\_ION\_TRANSMEMBRANE\_TRANSPORT |  | 32 | 0.17 | 0.62 | 0.988 | 1.000 | 1.000 | 3939 | tags=41%, list=31%, signal=58% |
| 942 | GOBP\_CELL\_COMMUNICATION\_BY\_ELECTRICAL\_COUPLING |  | 28 | 0.17 | 0.62 | 0.961 | 1.000 | 1.000 | 1177 | tags=14%, list=9%, signal=16% |
| 943 | GOBP\_DENDRITIC\_CELL\_CHEMOTAXIS |  | 25 | 0.18 | 0.62 | 0.957 | 1.000 | 1.000 | 3083 | tags=28%, list=24%, signal=37% |
| 944 | GOBP\_VENTRICULAR\_CARDIAC\_MUSCLE\_TISSUE\_MORPHOGENESIS |  | 45 | 0.16 | 0.61 | 0.980 | 1.000 | 1.000 | 4299 | tags=47%, list=33%, signal=70% |
| 945 | GOBP\_CELLULAR\_RESPONSE\_TO\_DEXAMETHASONE\_STIMULUS |  | 22 | 0.18 | 0.61 | 0.963 | 1.000 | 1.000 | 2466 | tags=27%, list=19%, signal=34% |
| 946 | GOBP\_REGULATION\_OF\_GOLGI\_ORGANIZATION |  | 16 | 0.20 | 0.61 | 0.962 | 1.000 | 1.000 | 3896 | tags=44%, list=30%, signal=63% |
| 947 | GOBP\_POSITIVE\_REGULATION\_OF\_ALPHA\_BETA\_T\_CELL\_DIFFERENTIATION |  | 45 | 0.15 | 0.61 | 0.981 | 1.000 | 1.000 | 2861 | tags=24%, list=22%, signal=31% |
| 948 | GOBP\_NEGATIVE\_REGULATION\_OF\_PHOSPHOPROTEIN\_PHOSPHATASE\_ACTIVITY |  | 20 | 0.19 | 0.61 | 0.964 | 1.000 | 1.000 | 3239 | tags=35%, list=25%, signal=47% |
| 949 | GOBP\_SULFUR\_AMINO\_ACID\_BIOSYNTHETIC\_PROCESS |  | 15 | 0.20 | 0.61 | 0.947 | 1.000 | 1.000 | 3324 | tags=27%, list=26%, signal=36% |
| 950 | GOBP\_REGULATION\_OF\_NMDA\_RECEPTOR\_ACTIVITY |  | 20 | 0.19 | 0.61 | 0.955 | 1.000 | 1.000 | 2097 | tags=20%, list=16%, signal=24% |
| 951 | GOBP\_POST\_GOLGI\_VESICLE\_MEDIATED\_TRANSPORT |  | 87 | 0.14 | 0.61 | 1.000 | 1.000 | 1.000 | 2687 | tags=20%, list=21%, signal=25% |
| 952 | GOBP\_POSTSYNAPTIC\_MEMBRANE\_ORGANIZATION |  | 28 | 0.17 | 0.61 | 0.970 | 1.000 | 1.000 | 3242 | tags=29%, list=25%, signal=38% |
| 953 | GOBP\_CELL\_VOLUME\_HOMEOSTASIS |  | 25 | 0.18 | 0.60 | 0.954 | 1.000 | 1.000 | 3225 | tags=32%, list=25%, signal=43% |
| 954 | GOBP\_NEGATIVE\_REGULATION\_OF\_PHOSPHATASE\_ACTIVITY |  | 27 | 0.17 | 0.59 | 0.972 | 1.000 | 1.000 | 3239 | tags=30%, list=25%, signal=39% |
| 955 | GOBP\_NEUROMUSCULAR\_JUNCTION\_DEVELOPMENT |  | 37 | 0.16 | 0.59 | 0.988 | 1.000 | 1.000 | 4108 | tags=35%, list=32%, signal=51% |
| 956 | GOBP\_REGULATION\_OF\_VIRAL\_INDUCED\_CYTOPLASMIC\_PATTERN\_RECOGNITION\_RECEPTOR\_SIGNALING\_PATHWAY |  | 21 | 0.18 | 0.59 | 0.966 | 1.000 | 1.000 | 10562 | tags=100%, list=82%, signal=551% |
| 957 | GOBP\_BONE\_MATURATION |  | 23 | 0.18 | 0.59 | 0.971 | 1.000 | 1.000 | 3916 | tags=48%, list=30%, signal=69% |
| 958 | GOBP\_CELL\_PROLIFERATION\_IN\_FOREBRAIN |  | 15 | 0.20 | 0.59 | 0.960 | 1.000 | 1.000 | 7374 | tags=93%, list=57%, signal=218% |
| 959 | GOBP\_PODOSOME\_ASSEMBLY |  | 16 | 0.19 | 0.58 | 0.968 | 1.000 | 1.000 | 2938 | tags=25%, list=23%, signal=32% |
| 960 | GOBP\_RESPONSE\_TO\_STEROL\_DEPLETION |  | 16 | 0.19 | 0.58 | 0.969 | 1.000 | 1.000 | 205 | tags=6%, list=2%, signal=6% |
| 961 | GOBP\_CELLULAR\_RESPONSE\_TO\_STEROL\_DEPLETION |  | 15 | 0.20 | 0.58 | 0.968 | 1.000 | 1.000 | 205 | tags=7%, list=2%, signal=7% |
| 962 | GOBP\_RESPONSE\_TO\_COPPER\_ION |  | 33 | 0.15 | 0.58 | 0.983 | 1.000 | 1.000 | 3225 | tags=30%, list=25%, signal=40% |
| 963 | GOBP\_BLASTODERM\_SEGMENTATION |  | 18 | 0.18 | 0.58 | 0.955 | 1.000 | 1.000 | 3704 | tags=39%, list=29%, signal=54% |
| 964 | GOBP\_REGULATION\_OF\_LONG\_TERM\_NEURONAL\_SYNAPTIC\_PLASTICITY |  | 20 | 0.18 | 0.57 | 0.972 | 1.000 | 1.000 | 2650 | tags=25%, list=21%, signal=31% |
| 965 | GOBP\_TELOMERASE\_RNA\_LOCALIZATION |  | 18 | 0.18 | 0.57 | 0.984 | 1.000 | 1.000 | 3464 | tags=39%, list=27%, signal=53% |
| 966 | GOBP\_POSITIVE\_REGULATION\_OF\_DENDRITIC\_SPINE\_MORPHOGENESIS |  | 15 | 0.19 | 0.56 | 0.982 | 1.000 | 1.000 | 10450 | tags=100%, list=81%, signal=526% |
| 967 | GOBP\_SUBPALLIUM\_DEVELOPMENT |  | 17 | 0.18 | 0.56 | 0.969 | 1.000 | 1.000 | 1404 | tags=12%, list=11%, signal=13% |
| 968 | GOBP\_REGULATION\_OF\_RIG\_I\_SIGNALING\_PATHWAY |  | 18 | 0.18 | 0.56 | 0.972 | 1.000 | 1.000 | 10562 | tags=100%, list=82%, signal=551% |
| 969 | GOBP\_POSITIVE\_REGULATION\_OF\_LEUKOCYTE\_APOPTOTIC\_PROCESS |  | 21 | 0.17 | 0.56 | 0.976 | 1.000 | 1.000 | 2692 | tags=24%, list=21%, signal=30% |
| 970 | GOBP\_NEGATIVE\_REGULATION\_OF\_REACTIVE\_OXYGEN\_SPECIES\_METABOLIC\_PROCESS |  | 38 | 0.14 | 0.56 | 0.997 | 1.000 | 1.000 | 2097 | tags=16%, list=16%, signal=19% |
| 971 | GOBP\_PYRIMIDINE\_NUCLEOSIDE\_MONOPHOSPHATE\_METABOLIC\_PROCESS |  | 18 | 0.18 | 0.56 | 0.981 | 1.000 | 1.000 | 1852 | tags=17%, list=14%, signal=19% |
| 972 | GOBP\_ETHANOL\_METABOLIC\_PROCESS |  | 16 | 0.18 | 0.55 | 0.980 | 1.000 | 1.000 | 2881 | tags=25%, list=22%, signal=32% |
| 973 | GOBP\_REGULATION\_OF\_RETINOIC\_ACID\_RECEPTOR\_SIGNALING\_PATHWAY |  | 15 | 0.18 | 0.55 | 0.966 | 1.000 | 1.000 | 3842 | tags=40%, list=30%, signal=57% |
| 974 | GOBP\_MITOCHONDRIAL\_DEPOLARIZATION |  | 18 | 0.18 | 0.55 | 0.986 | 1.000 | 1.000 | 1765 | tags=17%, list=14%, signal=19% |
| 975 | GOBP\_GOLGI\_TO\_PLASMA\_MEMBRANE\_PROTEIN\_TRANSPORT |  | 35 | 0.14 | 0.55 | 0.995 | 1.000 | 1.000 | 2370 | tags=17%, list=18%, signal=21% |
| 976 | GOBP\_AXON\_ENSHEATHMENT\_IN\_CENTRAL\_NERVOUS\_SYSTEM |  | 21 | 0.16 | 0.55 | 0.979 | 1.000 | 1.000 | 2083 | tags=19%, list=16%, signal=23% |
| 977 | GOBP\_NEGATIVE\_REGULATION\_OF\_OXIDOREDUCTASE\_ACTIVITY |  | 21 | 0.17 | 0.54 | 0.977 | 1.000 | 1.000 | 4172 | tags=43%, list=32%, signal=63% |
| 978 | GOBP\_FC\_GAMMA\_RECEPTOR\_SIGNALING\_PATHWAY |  | 27 | 0.15 | 0.54 | 0.981 | 1.000 | 1.000 | 2237 | tags=19%, list=17%, signal=22% |
| 979 | GOBP\_BILE\_ACID\_AND\_BILE\_SALT\_TRANSPORT |  | 22 | 0.16 | 0.53 | 0.987 | 1.000 | 1.000 | 2051 | tags=18%, list=16%, signal=22% |
| 980 | GOBP\_EXPLORATION\_BEHAVIOR |  | 21 | 0.16 | 0.53 | 0.983 | 1.000 | 1.000 | 1121 | tags=10%, list=9%, signal=10% |
| 981 | GOBP\_PROTEIN\_CONTAINING\_COMPLEX\_REMODELING |  | 31 | 0.15 | 0.53 | 0.990 | 1.000 | 1.000 | 1855 | tags=16%, list=14%, signal=19% |
| 982 | GOBP\_PROTEIN\_CATABOLIC\_PROCESS\_IN\_THE\_VACUOLE |  | 18 | 0.16 | 0.51 | 0.988 | 1.000 | 1.000 | 3735 | tags=39%, list=29%, signal=55% |
| 983 | GOBP\_NEUROTRANSMITTER\_REUPTAKE |  | 34 | 0.13 | 0.51 | 0.991 | 1.000 | 1.000 | 1972 | tags=15%, list=15%, signal=17% |
| 984 | GOBP\_REGULATION\_OF\_T\_CELL\_RECEPTOR\_SIGNALING\_PATHWAY |  | 39 | 0.13 | 0.50 | 0.997 | 1.000 | 1.000 | 3949 | tags=36%, list=31%, signal=52% |
| 985 | GOBP\_KINETOCHORE\_ORGANIZATION |  | 19 | 0.16 | 0.50 | 0.993 | 1.000 | 1.000 | 4110 | tags=42%, list=32%, signal=62% |
| 986 | GOBP\_COLLATERAL\_SPROUTING |  | 23 | 0.15 | 0.50 | 0.986 | 1.000 | 1.000 | 3381 | tags=26%, list=26%, signal=35% |
| 987 | GOBP\_REGULATION\_OF\_SMAD\_PROTEIN\_SIGNAL\_TRANSDUCTION |  | 27 | 0.13 | 0.48 | 0.998 | 1.000 | 1.000 | 11174 | tags=100%, list=87%, signal=745% |
| 988 | GOBP\_CYTOPLASMIC\_PATTERN\_RECOGNITION\_RECEPTOR\_SIGNALING\_PATHWAY\_IN\_RESPONSE\_TO\_VIRUS |  | 31 | 0.13 | 0.45 | 1.000 | 1.000 | 1.000 | 5303 | tags=58%, list=41%, signal=98% |
| 989 | GOBP\_MITOPHAGY |  | 30 | 0.12 | 0.45 | 1.000 | 1.000 | 1.000 | 3939 | tags=30%, list=31%, signal=43% |
| 990 | GOBP\_POSITIVE\_REGULATION\_OF\_DENDRITIC\_SPINE\_DEVELOPMENT |  | 28 | 0.12 | 0.43 | 1.000 | 1.000 | 1.000 | 11313 | tags=100%, list=88%, signal=811% |
| 991 | GOBP\_MAINTENANCE\_OF\_GASTROINTESTINAL\_EPITHELIUM |  | 18 | 0.13 | 0.41 | 1.000 | 1.000 | 1.000 | 11199 | tags=100%, list=87%, signal=757% |
Table: Gene sets enriched in phenotype **OA (10 samples) **[plain text format]****
